# Insight Into the Metabolic Adaptations of Electrically Pulse-Stimulated Human Myotubes Using Global Analysis of the Transcriptome and Proteome

**DOI:** 10.3389/fphys.2022.928195

**Published:** 2022-07-06

**Authors:** Abel M. Mengeste, Nataša Nikolić, Andrea Dalmao Fernandez, Yuan Z. Feng, Tuula A. Nyman, Sander Kersten, Fred Haugen, Eili Tranheim Kase, Vigdis Aas, Arild C. Rustan, G. Hege Thoresen

**Affiliations:** ^1^ Section for Pharmacology and Pharmaceutical Biosciences, Department of Pharmacy, University of Oslo, Oslo, Norway; ^2^ Department of Immunology, Institute of Clinical Medicine, University of Oslo and Oslo University Hospital, Oslo, Norway; ^3^ Division of Human Nutrition and Health, Wageningen University, Wageningen, Netherlands; ^4^ Department of Work Psychology and Physiology, STAMI—The National Institute of Occupational Health, Oslo, Norway; ^5^ Department of Life Sciences and Health, Faculty of Health Sciences, OsloMet—Oslo Metropolitan University, Oslo, Norway; ^6^ Department of Pharmacology, Institute of Clinical Medicine, University of Oslo, Oslo, Norway

**Keywords:** exercise, skeletal muscle, energy metabolism, myokines, omics

## Abstract

Electrical pulse stimulation (EPS) has proven to be a useful tool to interrogate cell-specific responses to muscle contraction. In the present study, we aimed to uncover networks of signaling pathways and regulatory molecules responsible for the metabolic effects of exercise in human skeletal muscle cells exposed to chronic EPS. Differentiated myotubes from young male subjects were exposed to EPS protocol 1 (i.e. 2 ms, 10 V, and 0.1 Hz for 24 h), whereas myotubes from middle-aged women and men were exposed to protocol 2 (i.e. 2 ms, 30 V, and 1 Hz for 48 h). Fuel handling as well as the transcriptome, cellular proteome, and secreted proteins of EPS-treated myotubes from young male subjects were analyzed using a combination of high-throughput RNA sequencing, high-resolution liquid chromatography-tandem mass spectrometry, oxidation assay, and immunoblotting. The data showed that oxidative metabolism was enhanced in EPS-exposed myotubes from young male subjects. Moreover, a total of 81 differentially regulated proteins and 952 differentially expressed genes (DEGs) were observed in these cells after EPS protocol 1. We also found 61 overlapping genes while comparing the DEGs to mRNA expression in myotubes from the middle-aged group exposed to protocol 2, assessed by microarray. Gene ontology (GO) analysis indicated that significantly regulated proteins and genes were enriched in biological processes related to glycolytic pathways, positive regulation of fatty acid oxidation, and oxidative phosphorylation, as well as muscle contraction, autophagy/mitophagy, and oxidative stress. Additionally, proteomic identification of secreted proteins revealed extracellular levels of 137 proteins were changed in myotubes from young male subjects exposed to EPS protocol 1. Selected putative myokines were measured using ELISA or multiplex assay to validate the results. Collectively, our data provides new insight into the transcriptome, proteome and secreted proteins alterations following *in vitro* exercise and is a valuable resource for understanding the molecular mechanisms and regulatory molecules mediating the beneficial metabolic effects of exercise.

## 1 Introduction

Regular exercise and physical activity are recognized as a cornerstone in the prevention and first-line treatment of several chronic diseases including obesity, type 2 diabetes (T2D), and cardiovascular diseases (Colberg et al., 2010; Egan and Zierath, 2013; Pedersen and Saltin, 2015; Anderson and Durstine, 2019). Although the exact underlying mechanisms for the beneficial effects of exercise remain to be elucidated, adaptations of skeletal muscle are considered to be essential (Booth and Thomason, 1991; Flück, 2006).

Skeletal muscle is a highly plastic tissue, as it has the ability to modify the structure and function in response to external stimuli imposed on it (Flück and Hoppeler, 2003). Repeated bouts of muscle contraction, along with regular endurance or aerobic exercise training, are potent stimuli for skeletal muscle adaptations (Egan and Zierath, 2013). This stimulation of skeletal muscle by exercise bouts is integrated by a multitude of complex signaling networks, where the physiological response varies by the frequency, intensity, and duration of the exercise (Hoppeler et al., 2011) as well as intrinsic factors such as age and body composition (Egan and Zierath, 2013). From a metabolic point of view, exercise-mediated adaptations in skeletal muscle are reflected by an increase in mitochondrial biogenesis (Holloszy and Coyle, 1984; Roberts and Markby, 2021), improved oxidative capacity of glucose and fatty acids as well as enhanced insulin-stimulated glucose uptake (Turcotte and Fisher, 2008). Moreover, it is increasingly evident that skeletal muscle can produce and secret cytokines and other peptides (referred to as myokines) in response to physical exercise and muscle contraction (Norheim et al., 2011; Pedersen and Febbraio, 2012). To date, hundreds of myokines have been identified including various interleukins (IL-6, IL-8 and IL-15), leukemia inhibitory factor (LIF), irisin and follistatin-like protein-1 (FSTL-1) (Das et al., 2020). These proteins in turn can exert either autocrine, paracrine, and/or endocrine effects, suggesting that the beneficial effects of exercise could at least in part be mediated by crosstalk between muscle and other organs.

To further elucidate the complexity of exercise response, *in vitro* exercise models of electrical pulse stimulation (EPS) have been widely utilized in cultured myotubes to mimic muscle contraction (Aas et al., 2002; Lambernd et al., 2012; Nikolić et al., 2012; Feng et al., 2015; Løvsletten et al., 2020). The usefulness of EPS to study molecular processes that are occurring during exercise and its suitability for identification of exercise-induced myokines have been reported in studies using both murine C2C12 myotubes and human primary myotubes (Lambernd et al., 2012; Nikolić et al., 2012; Scheler et al., 2013; Evers-van Gogh et al., 2015). Although this model lacks the physiological changes evoked during whole-body exercise, including alterations of microenvironment and circulating hormones, as well as increased blood flow, the application of this model in human muscle cells could be important to investigate specific aspects of contraction-mediated signaling pathways (Nikolić et al., 2017). Additionally, this model provides the possibility to combine different stimulation parameters (i.e. frequency, output voltage, and duration of pulse trains), enabling the study of skeletal muscle adaptations to “different exercise modalities” (Nikolić et al., 2017). Although direct comparison of *in vitro* protocols of exercise to different types of *in vivo* exercise training is difficult, we were able to show that high-frequency, acute EPS (i.e., up to 60 min) simulated some features of a single bout of exercise, whereas low-frequency, chronic EPS (i.e., 24-48 h) led to responses known to be mediated by regular exercise *in vivo* (Nikolić et al., 2012).

Despite the profound progress made over the last decade to fill critical gaps in our understanding of exercise-induced metabolic adaptations, more work is needed to integrate the molecular and cellular networks involved in the responses to skeletal muscle contraction. In this study, we aimed to investigate mechanisms involved in EPS-mediated metabolic adaptations in human skeletal muscle cells. The global gene and protein expression in cell lysates and secreted proteins were also studied to further uncover networks of signaling pathways and regulatory molecules responsible for the metabolic effects of this *in vitro* exercise model.

## 2 Materials and Methods

### 2.1 Materials

Dulbecco’s Modified Eagle’s Medium (DMEM)-Glutamax™ low glucose, Dubecco’s Phosphate Buffered Salin (DPBS; without Ca^2+^ and Mg^2+^), heat-inactivated fetal calf serum (FCS), penicillin-streptomycin (10,000 IE/ml), amphotericin B, human epidermal growth factor, and gentamycin were purchased from Gibco Invitrogen (Gibco, Life Technologies, Paisley, United Kingdom). Insulin (Actrapid^®^ Penfill^®^ 100 IE/ml) was from NovoNodisk (Bagsvaerd, Denmark). Tris-HCl, sodium dodecyl sulfate (SDS), Bio-Rad Protein Assay Dye Reagent Concentrate, Bio-Rad Precision Plus Protein™ Dual Color standard, bromophenol blue, Trans-Blot^®^ Turbo™ Mini-size Transfer nitrocellulose membranes, Trans-Blot^®^ Turbo™ Mini-size Transfer Stacks, Mini-Protean^®^ TGX™ gels (4%–20%), Tween 20 and Tris/glycine/SDS buffer were from Bio-Rad (Copenhagen, Denmark). Antibodies against total and phosphorylated AMP-activated protein kinase (AMPK) α at Thr172 (#2531 and #2532, respectively) were from Cell Signalling Technology^®^ Inc (Beverly, MA, United States). Intercept^®^ (Tris-buffered salin, TBS) blocking buffer (#927-60001), donkey anti-rabbit IR Dye 680RD (#926-68073) and donkey anti-rabbit IR Dye 800CW (#926-32213) were from LI-COR Biosciences (Lincoln, NE, United States). QIAshredder and RNeasy Mini Kit were from QIAGEN (Venlo, Netherlands). D-[^14^C]glucose (3.0 mCi/mmol), 2-[^3^H(G)]deoxy-d-glucose (10 Ci/mmol) and [1–^14^C]oleic acid (OA, 59.0 mCi/mmol) were from PerkinElmer NEN^®^ (Boston, MA, United States). Ultima Gold™ XR, Pico Prias 6 ml PE vials, 96-well Isoplate^®^, UniFilter^®^-96 GF/B microplates, and TopSeal^®^-A transparent film were obtained from PerkinElmer (Shelton, CT, United States). 6-well Corning^®^ CellBIND^®^ tissue culture plates were from Corning (Schiphol-Rijk, Netherlands). Human recombinant LIF was purchased from Chemicon (Temecula, CA, United States). Bio-Plex^®^ Handheld Magnetic Washer, Bio-Plex^®^ MAGPIX™ plate reader were from Bio-Rad Laboratories Inc (Hercules, CA, United States).

### 2.2 Methods

#### 2.2.1 Donor Characteristics and Cell Culturing of Human Skeletal Muscle Cells

The cells were isolated from biopsy samples of the *musculus (m.) vastus lateralis* from seven young male subjects as previously described (Lund et al., 2018). These donors were in the age of 23.4 ± 0.9 years with a mean body mass index (BMI) 23.8 ± 0.8 kg/m^2^, and were used in EPS protocol 1 (described below). For EPS protocol 2 (described below), biopsies were taken from *m. vastus lateralis* or *m. obliquus internus abdominis* from 12 women and six men, age 50.4 ± 1.7 years with BMI 29.7 ± 2.3 kg/m^2^. Of these donors, cells established from four middle-aged women (49.8 ± 2.2 years) with a mean BMI of 24.3 ± 1.7 kg/m^2^ were used in the microarray analysis experiments following EPS protocol 2 (described below). Written informed consent was given from all donors prior to biopsy sampling, and the procedure was carried out in accordance with the ethical standards of the Regional Committee for Medical and Health Research Ethics (REK) South East, Oslo, Norway (reference numbers: 2011/2207, S-04133, S-09078d 2009/166) and REK North, Tromsø, Norway (reference number: 2011/882). For isolation of human satellite cells, muscle biopsies were washed with DPBS (without Ca^2+^ and Mg^2+^), minced, and dissociated for 60 min by three successive treatments of 0.1% trypsin-EDTA. Dissociated cells were pooled and 10% FCS was added as a protease inhibitor before centrifuged at 1800 rpm for 7 min. The cells were resuspended in PromoCell medium supplemented with PromoCell Supplement Mix, penicillin (25 IU), streptomycin (25 μg/ml), and amphotericin B (1.25 μg/ml) and then cultured in a 25 cm^2^ NUNC^TM^ Cell Culture Treated Flasks. Medium was changed regularly every 2–3 days until 80–90% confluence was achieved. Cells were precultured once or twice depending on the number of cells before final seeding using DMEM-Glutamax (5.5 mM glucose) with supplements during proliferation and differentiation to establish multinucleated myotubes, as previously described (Mengeste et al., 2021).

#### 2.2.2 Electrical Pulse Stimulation (EPS) of Muscle Cells

Skeletal muscle cells were seeded at a density of 100,000 cells/well on 6-well CelBIND^®^ microplates, proliferated and differentiated into myotubes. Two different chronic, low-frequency EPS protocols were used. In EPS protocol 1, the myotubes were differentiated for 6 days, washed three times with PBS, and the media were changed to serum-free differentiation media before a single, bipolar pulses of 2 ms, with voltage 10 V and frequency 0.1 Hz were applied continuously for 24 h using a C-Dish™ carbon electrode connected to a C-Pace EP multi-channel culture pacer (IonOptix, Dublin, Ireland). In EPS protocol 2, the cells were differentiated for 5 days and exposed to EPS with 2 ms pulse duration, 30 V intensity, and frequency 1 Hz for the last 48 h of differentiation as previously described (Nikolić et al., 2019). In both protocols, control myotubes cultured on 6-well CelBIND^®^ microplates were fitted with a C-Dish™ carbon electrode without connecting the C-Dish to the pulse generator.

#### 2.2.3 Glucose and Fatty Acid Metabolism

Substrate metabolism in differentiated primary human myotubes was assessed using the radioactive substrates [^14^C]glucose and [^14^C]oleic acid. Briefly, myotubes were cultured on 6-well plates, and EPS protocol 1 (2 ms, 10 V and 0.1 Hz) was applied for the last 24 h of the differentiation period. The myotubes were then exposed to 1 ml/well DPBS supplemented with HEPES (10 mM), NaHCO_3_ (44 mM), BSA (10 μM) and D-[^14^C(U)]glucose (0.5 μCi/ml, 200 μM) for 3 h. Alternatively, the cells were incubated with [1–^14^C]oleic acid (0.5 μCi/ml, 100 μM) in DPBS supplemented with HEPES (10 mM), NaHCO_3_ (44 mM), BSA (6.8 μM) and l-carnitine (1 mM) in a CO_2_ incubator (5%), humidified and kept at 37°C. Following incubation, 200 μl of the radioactive media from each well were transferred to a 96-well plate, and the cells were washed twice with PBS before lysed in 500 μl of NaOH (0.1 M). The plates were then stored at −20°C until further analyzed. The amount of ^14^CO_2_ produced from cellular respiration was measured by the addition of perchloric acid (1 M) to the frozen media and trapping of CO_2_ was performed for 4 h as described previously (Wensaas et al., 2007). The trapped CO_2_ and the cell-associated (CA) radioactivity were then determined using a 2450 MicroBeta^2^ liquid scintillation counter (PerkinElmer). For analysis of the data, GraphPad Prism 8.0.1 for Windows (GraphPad Software Inc., San Diego, CA, United States) was used. All data were related to the total cellular protein content and reported as mean ± SEM. The value *n* represents number of experimental replicates. Significance of difference between the groups was evaluated by unpaired Student’s *t*-test, where a *p-*value < 0.05 was considered significant.

#### 2.2.4 Immunoblotting

Total protein from cultured myotubes treated with EPS protocol 1 were prepared using Laemmli buffer (0.5 M Tris-HCl, 10% SDS, 20% glycerol, 10% β-mercaptoethanol, and 5% bromophenol blue). The proteins were electrophoretically separated on 4%–20% Mini-Protean TGX™ gels with Tris/glycine buffer (pH 8.3) followed by blotting to nitrocellulose membranes. The membranes were then incubated with primary antibodies at 4°C overnight. After several washes with Tris-buffered saline containing 0.1% Tween-20, the appropriate fluorescent secondary antibodies were added and incubated for 1 h at room temperature. Specific bands were visualized by infrared scanning by Odyssey CLx imaging system (LI-COR Biosciences, Lincoln, NE, United States). The blots were then quantified using Image Studio™ Lite (version 5.2.5) software.

#### 2.2.5 Deoxyglucose Uptake

Human myotubes were cultured in 6-well tissue culture plates, washed three times with PBS and incubated for 15 min (37°C, 5% CO_2_) in a buffer containing NaCl (140 mM), HEPES (20 mM), KCl (5 mM), MgSO_4_ (2.5 mM), CaCl_2_ (1 mM), 2-[^3^H(G)]deoxy-d-glucose (1 μCi/ml), 10 μM deoxyglucose, and LIF 0.01–1 nM. Following incubation, the cells were washed three times with ice-cold PBS, lysed with 0.05 M NaOH, and homogenized by ultra-sound sonication for 10 s before radioactivity was counted by liquid scintillation (Packard Tri-Carb 1900 TR, PerkinElmer). The amount of radioactivity taken up by the cells was then related to the protein content of each sample determined according to Bradford (Bradford, 1976). Noncarrier-mediated uptake was determined in the presence of cytochalasin B (10 μM) and subtracted from all presented values. Statistical analysis of the data was performed using GraphPad Prism 8.0.1 for Windows (GraphPad Software Inc., San Diego, CA, United States). The results are presented as normalized to untreated control cells, where the value *n* represents the number of experimental replicates. Unpaired Student’s *t*-test was employed to determine effects of treatment, where a *p*-value < 0.05 was considered significant.

#### 2.2.6 Preparation of Conditioned Media From Primary Human Myotubes

Primary human myotubes were grown and submitted to EPS protocol 1. After completion of EPS, the serum-free media were collected and centrifuged at 1,400 rpm for 7 min at 4°C to remove detached cells and cellular debris. The supernatant was then carefully transferred to a new 15 ml centrifuge tube and kept frozen at −80°C.

#### 2.2.7 Proteome Analysis

Proteome analysis was performed at Proteomic Core Facility (Dept of Immunology, University of Oslo and Oslo University Hospital, Oslo, Norway). Proteins in the conditioned media and cell proteins were analyzed from EPS-stimulated (protocol 1) and unstimulated control myotubes taken from seven young male subjects using a global proteomic approach. In brief, the media samples were first concentrated using 10 kDa Amicon^®^ Ultra centrifugal filters (Merck) followed by protein reduction, alkylation, and trypsin (Promega) digestion. The resulting peptide mixtures were purified by the STAGE-TIP method using a C18 resin disk (3 M Empore) before the samples were analyzed by a nanoLC-MS/MS using nEASY-LC coupled to QExacitvePlus (Thermo Electron, Bremen, Germany) using a 60 min LC separation gradient.

Cultured myotubes were harvested in Laemmli buffer (0.5 M Tris-HCl, 10% SDS and 20% glycerol). All samples were prepared using a Protein Aggregation Capture on Microparticles method, described previously (Batth et al., 2019) followed by protein reduction, alkylation, and trypsin digestion. The resulting tryptic peptides were analyzed using EvoSepOne coupled to timsTOF Flex (Bruker) using 88 min separation gradient.

The resulting LC-MS/MS files from secreted proteins and cell proteome analysis were processed with MaxQuant version 1.6.1.0 and 1.6.17.0, respectively, for protein identification and label-free quantification (LFQ) using default settings. The searches were done against the human Uniprot Reference Proteome database (September 2018 and December 2020). Perseus software version 1.6.1.3 and 1.6.14.0 were used for the statistical analysis of the total secreted proteins and cell proteome MaxQuant results, respectively. Here, a paired two-tailed Student’s *t*-test (*p* < 0.05) was performed to determine differences in protein secretion or expression between EPS-treated and unstimulated myotubes. Protein ANalysis THrough Evolutionary Relationships (PANTHER, http://www.pantherdb.org/, version 16.0) analysis tool (Mi et al., 2021), the web-based enrichment analysis tool EnrichR (Kuleshov et al., 2016) (http://amp.pharm.mssm.edu/Enrichr) and ShinyGO online analysis tool (Ge et al., 2020) (http://ge-lab.org/go/) were used to group differentially regulated protein into protein class and Gene Ontology (GO) Biological Process annotations. The human MitoCarta 3.0 (Rath et al., 2021) was used to identify mitochondrial proteins from our list of significantly regulated proteins. The mass spectrometry proteomics data are deposited to the ProteomeXchange Consortium via the PRIDE with the dataset identifier PXD033025.

#### 2.2.8 Isolation of RNA and High Throughput RNA Sequencing

After treatment with EPS protocol 1, total RNA from cultured myotubes of young male subjects was extracted using the RNeasy mini kit according to manufacturer’s protocol. The quantity and quality of RNA, as well as preparation of RNA library and transcriptome sequencing was performed by Novogene Co., LTD. (Milton, United Kingdom). The library preparations were sequenced on their Illumina platform and paired-end sequencing pipeline.

Raw data (raw reads) of FASTQ format were first processed through fastp. Reference genome and gene model annotation files were downloaded from genome website browser (NCBI/UCSC/Ensembl) directly, and paired-end clean reads were aligned to the reference genome using the Spliced Transcripts Alignment to a Reference (STAR) software version 2.6.1d using default parameters. FeatureCounts version 1.5.0-p3 was used to count the read numbers mapped of each gene before Reads Per Kilobase of exone model per Million mapped reads (RPKM) of each gene was calculated based on the length of the gene and reads count mapped. Differential expression analysis of transcriptomes between stimulated and unstimulated control group was performed using DESeq2 edgeR package version 3.24.3. The resulting *p* value was adjusted using the Benjamini and Hochberg’s method for controlling the FDR. The PANTHER Classification system was employed to classify the DEGs according to protein class, while mitochondria-related DEGs were identified using MitoCarta 3.0. The web-based enrichment analysis tool EnrichR was used to test the statistical enrichment of differentially expressed genes (DEGs) at the biologically functional level. All *p* values < 0.05 were considered statistically significant. Sequence data are submitted to Gene Expression Omnibus and can be accessed through http://www.ncbi.nlm.nih.gov/geo with the ID: GSE200335.

#### 2.2.9 Affymetrix Microarray

Microarray analysis was performed on RNA of primary human myotubes from four middle-aged women donors exposed to EPS protocol 2. Purified RNA was labeled with the Ambion WT Expression kit (Life Technologies, Bleiswijk, Netherlands) and hybridized to an Affymetrix GeneChip Human Gene 1.0 ST array plate before scanning on an Affymetrix 3,000-7 G instrument (Santa Clara, CA, United States). Probe sets were defined according to Dai et al. (2005), in which probes are assigned to Entrez IDs as a unique gene identifier. Quartile data normalization and background correction were applied using the Robust Mutichip Average (RMA) (Irizarry et al., 2003), as previously described (Jans et al., 2012). Differentially expressed probe sets were determined using an intensity-based moderated paired t-statistics (Sartor et al., 2006). *p*-values were corrected for multiple testing using the False Discovery Rate (FDR) approach. Genes with a *q*-value < 0.2 and a fold change >1.2 were considered statistically significant. All microarray data are submitted to Gene Expression Omnibus and can be accessed through http://www.ncbi.nlm.nih.gov/geo with the ID: GSE40789 (control myotubes) and GSE201340 (EPS-stimulated myotubes).

#### 2.2.10 Multiplex Immunoassay and ELISA

Primary human skeletal muscle cells from middle-aged women and men were cultured and submitted to EPS protocol 2. After completion of EPS, culture media was collected and stored at −80°C until use. Commercially available human Bio-Plex Pro™ multiplex bead-based immunoassay was used to determine the concentration of LIF. The analysis was performed using Bio-Rad’s Bio-Plex^®^ MEGPIX™ suspension array system according to the manufacturer’s protocol. The calibration curve for the proteins was analyzed with 5 parametric logistic curve regression using Bio-Plex Manager™ software system (Bio-Rad, Hercules, CA). Commercially available Human IL-8/CXCL8 Quantikine ELISA Kit (R&D systems, Minneapolis, MN) was used to determine the concentration of C-X-C Motif Chemokine Ligand 8 (CXCL8), using the manufacturer’s assay procedure. The multiplex and ELISA data are presented as normalized to samples from unstimulated control cells, with *n* representing the number of experiments performed. Comparisons between groups were evaluated using two-tailed, paired Student’s *t*-test to assess statistical significance (*p*-value < 0.05).

## 3 Results

### 3.1 Effects of EPS on Substrate Metabolism and Activation of AMPK in Human Myotubes

**FIGURE 1:**
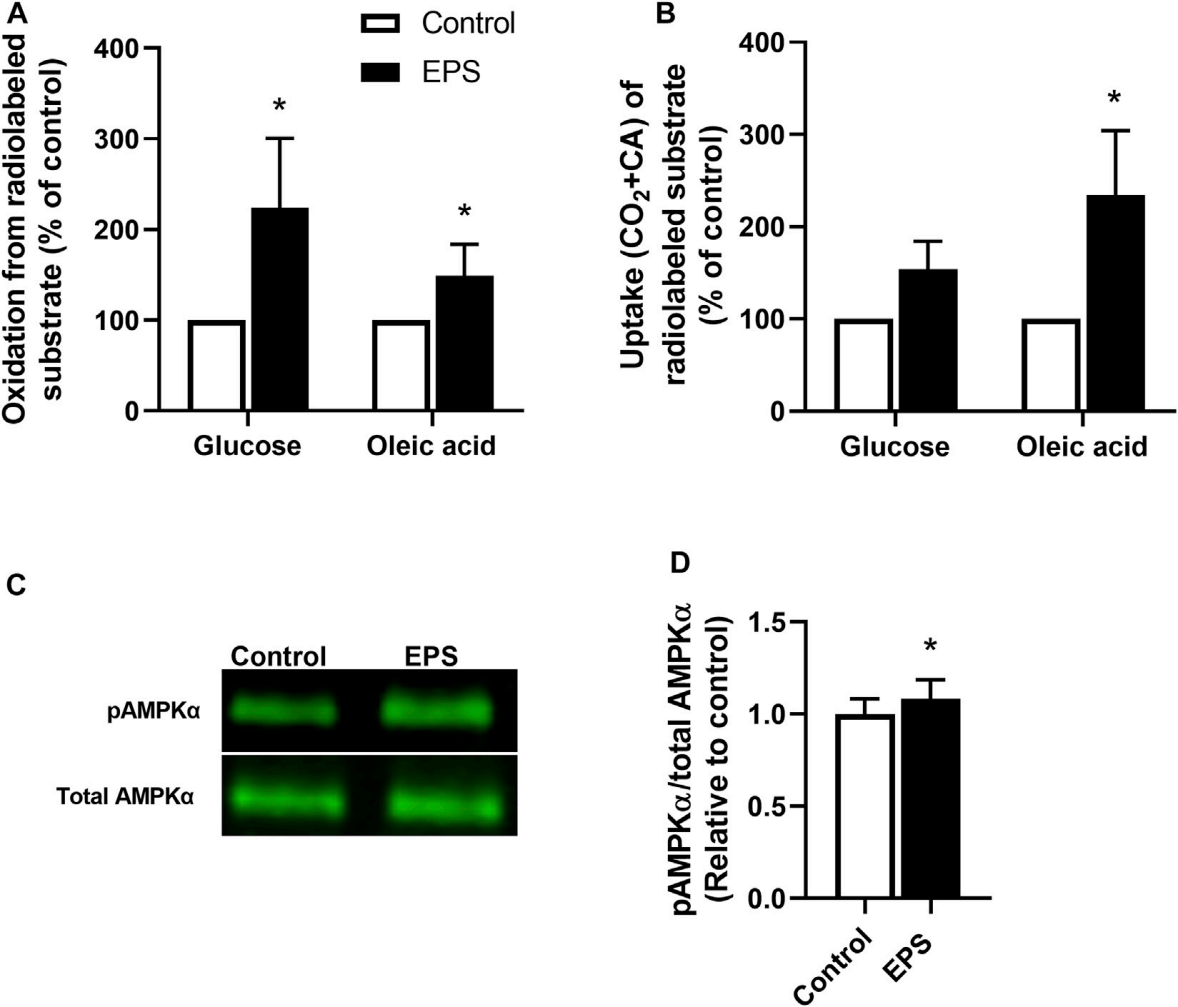
Changes in substrate metabolism and phosphorylation of AMP-activated protein kinase (AMPK) in myotubes after electrical pulse stimulation (EPS). Human myotubes from young male subjects were cultured on 6-well plates and exposed to EPS protocol 1 (2 ms, 10 V and 0.1 Hz for 24 h). **(A,B)** After EPS, myotubes were incubated with a medium containing [^14^C]glucose (0.5 μCi/ml, 200 μM) or [^14^C]oleic acid (0.5 μCi/ml, 100 μM). Cell-associated (CA) radioactivity and trapped CO_2_ (oxidation) were measured as described in methods. **(A)** Oxidation of glucose and oleic acid after EPS. Data are expressed as mean ± SEM, normalized to unstimulated control cells (absolute values: glucose; 0.39 ± 0.17 nmol/mg protein and oleic acid; 0.32 ± 0.14 nmol/mg protein). **(B)** Glucose and oleic acid uptake quantified as the sum of trapped CO_2_ and the remaining CA radioactivity in cells. Values are presented as mean ± SEM, normalized to unstimulated control cells (absolute values: glucose 25.4 ± 11.4 nmol/mg protein, oleic acid 103.7 ± 46.4 nmol/mg protein). **(C,D)** Aliquots of total cell lysates were prepared in Laemmli buffer and electrophoretically separated before phosphorylation of AMPKα was assessed by immunoblotting using a phospho-specific AMPKα^Thr172^ antibody. **(C)** Representative immunoblots and **(D)** quantified protein expression of AMPKα^Thr172^ phosphorylation. Expression of pAMPKα^Thr172^ was normalized to total AMPKα^Thr172^ expression. All results are presented as mean ± SEM from five (*n* = 5, **(A,B)** and six (*n* = 6, **(D)** individual experiments. *p < 0.05 vs. control.

We used the EPS protocol 1 (i.e., 2 ms, 10 V, and 0.1 Hz for 24 h) in myotubes established from young male subjects (BMI 23.8 ± 0.8 kg/m^2^, age 23.4 ± 0.9 years) to explore substrate metabolism in skeletal muscle cells. The effects of EPS were determined by measuring the oxidation and uptake of glucose and oleic acid. Our results showed that both glucose and oleic acid oxidation were increased following the EPS protocol 1 (Figure 1A), showing that this EPS protocol can improve oxidative metabolism in a similar fashion to exercise *in vivo*, as previously shown for protocol 2 (Nikolić et al., 2012). Moreover, total cellular uptake of oleic acid in myotubes was enhanced by EPS protocol 1, whereas no significant difference was observed for glucose uptake (Figure 1B). Activation of the cellular energy sensor, AMPK, was additionally assessed by immunoblotting. Our data showed that exposure of the cells from these young male subjects to EPS protocol 1 increased phosphorylation of AMPKα^Thr172^ compared to unstimulated control cells (Figures 1C,D).

### 3.2 Proteomic Identification of Differentially Expressed Cellular Proteins After EPS

To explore the cellular mechanisms behind the EPS-induced effects on energy substrate utilization, we used high-resolution mass spectrometry-based proteomic approach to compare global protein expression profiles of stimulated and unstimulated control myotubes from young male subjects (BMI 23.8 (±0.8) kg/m^2^, age 23.4 ± 0.9) submitted to EPS protocol 1. A total of 2482 proteins were identified. Among these, 1,366 proteins with a minimum of three label-free quantification values in at least one sample group were considered for further analysis. Compared to unstimulated control cells, 78 proteins were significantly upregulated in EPS-exposed myotubes, while only three proteins; lysosome-associated membrane glycoprotein 1 (LAMP1), 60S ribosomal protein L19 (RPL19) and monocarboxylate transporter 1 (SLC16A1) were found to be significantly downregulated (Table 1).

**TABLE 1:** Differentially expressed proteins identified in human skeletal muscle cells following electrical pulse stimulation (EPS) protocol 1 for 24 h.

Protein IDs	Gene names	Protein names	*p*-value	Fold change
P12829	*MYL4*	Myosin light chain 4	1,26E-02	8,76
Q8IWX7	*UNC45B*	Protein unc-45 homolog B	1,75E-02	6,86
P68032;P62736;P63267	*ACTC1; ACTA2; ACTG2*	Actin, alpha cardiac muscle 1;Actin, aortic smooth muscle;Actin, gamma-enteric smooth muscle	2,35E-02	5,03
P63316	*TNNC1*	Troponin C, slow skeletal and cardiac muscles	5,93E-03	5,01
P05976	*MYL1*	Myosin light chain 1/3, skeletal muscle isoform	1,69E-02	4,98
P35609	*ACTN2*	Alpha-actinin-2	3,90E-02	4,91
P13929	*ENO3*	Beta-enolase	9,34E-03	4,87
P35080	*PFN2*	Profilin-2	2,58E-02	4,86
Q99439	*CNN2*	Calponin-2	1,96E-02	4,82
O14950;P19105	*MYL12B; MYL12A*	Myosin regulatory light chain 12B;Myosin regulatory light chain 12A	3,51E-02	4,62
P45379	*TNNT2*	Troponin T, cardiac muscle	2,37E-02	4,57
P43034	*PAFAH1B1*	Platelet-activating factor acetylhydrolase IB subunit alpha	2,71E-04	4,53
P52943	*CRIP2*	Cysteine-rich protein 2	3,30E-02	4,41
Q96A32	*MYLPF*	Myosin regulatory light chain 2, skeletal muscle isoform	3,40E-02	4,21
Q0ZGT2	*NEXN*	Nexilin	4,59E-02	4,12
Q9BRF8	*CPPED1*	Serine/threonine-protein phosphatase CPPED1	3,21E-03	4,06
P13805	*TNNT1*	Troponin T, slow skeletal muscle	1,76E-02	4,00
Q01995	*TAGLN*	Transgelin	1,89E-02	3,98
Q9UHQ9	*CYB5R1*	NADH-cytochrome b5 reductase 1	3,05E-02	3,75
P13591	*NCAM1*	Neural cell adhesion molecule 1	4,40E-02	3,67
Q53FA7	*TP53I3*	Quinone oxidoreductase PIG3	4,43E-03	3,59
Q13404	*UBE2V1*	Ubiquitin-conjugating enzyme E2 variant 1	2,50E-02	3,53
Q2TBA0	*KLHL40*	Kelch-like protein 40	9,20E-03	3,45
P50502;Q8NFI4;Q8IZP2	*ST13; ST13P5; ST13P4*	Hsc70-interacting protein;Putative protein FAM10A5;Putative protein FAM10A4	4,76E-02	3,37
Q9UPN3	*MACF1*	Microtubule-actin cross-linking factor 1, isoforms 1/2/3/5	2,28E-02	3,30
Q13642	*FHL1*	Four and a half LIM domains protein 1	4,80E-02	3,29
P46976	*GYG1*	Glycogenin-1	1,25E-02	3,28
Q14192	*FHL2*	Four and a half LIM domains protein 2	2,91E-02	3,21
P49773	*HINT1*	Histidine triad nucleotide-binding protein 1	4,66E-02	3,19
Q9UPY8;Q15555	*MAPRE3*	Microtubule-associated protein RP/EB family member 3	3,05E-03	3,15
Q8TDZ2	*MICAL1*	Protein-methionine sulfoxide oxidase MICAL1	8,06E-03	3,09
Q5BKX8	*MURC*	Muscle-related coiled-coil protein	5,83E-03	3,04
P60891;P11908;P21108	*PRPS1; PRPS2*	Ribose-phosphate pyrophosphokinase 1;Ribose-phosphate pyrophosphokinase 2	3,86E-02	2,95
P21980	*TGM2*	Protein-glutamine gamma-glutamyltransferase 2	3,60E-02	2,84
P05198	*EIF2S1*	Eukaryotic translation initiation factor 2 subunit 1	2,45E-02	2,84
P21291	*CSRP1*	Cysteine and glycine-rich protein 1	4,79E-02	2,81
Q9Y570	*PPME1*	Protein phosphatase methylesterase 1	4,73E-02	2,78
O75947	*ATP5H*	ATP synthase subunit d, mitochondrial	2,10E-02	2,76
Q14444	*CAPRIN1*	Caprin-1	4,85E-02	2,70
P14649	*MYL6B*	Myosin light chain 6B	2,33E-03	2,66
Q9Y2Q3	*GSTK1*	Glutathione S-transferase kappa 1	4,64E-02	2,65
Q9UKM9	*RALY*	RNA-binding protein Raly	6,78E-03	2,61
O94875	*SORBS2*	Sorbin and SH3 domain-containing protein 2	3,57E-02	2,60
P61970	*NUTF2*	Nuclear transport factor 2	4,89E-02	2,58
P62333	*PSMC6*	26S protease regulatory subunit 10B	4,87E-02	2,53
P47897	*QARS*	Glutamine--tRNA ligase	2,93E-03	2,48
P17174	*GOT1*	Aspartate aminotransferase, cytoplasmic	1,74E-02	2,46
P67809	*YBX1*	Nuclease-sensitive element-binding protein 1	1,73E-02	2,43
P21912	*SDHB*	Succinate dehydrogenase [ubiquinone] iron-sulfur subunit, mitochondrial	1,44E-02	2,38
Q99497	*PARK7*	Protein deglycase DJ-1	4,77E-02	2,37
Q9P2J5	*LARS*	Leucine-tRNA ligase, cytoplasmic	4,92E-02	2,36
Q92499	*DDX1*	ATP-dependent RNA helicase DDX1	4,96E-02	2,33
P56556	*NDUFA6*	NADH dehydrogenase [ubiquinone] 1 alpha subcomplex subunit 6	5,47E-03	2,33
Q8WWI1	*LMO7*	LIM domain only protein 7	4,05E-02	2,30
P09104	*ENO2*	Gamma-enolase	2,71E-02	2,25
Q16270	*IGFBP7*	Insulin-like growth factor-binding protein 7	1,74E-02	2,24
Q9Y230	*RUVBL2*	RuvB-like 2	4,80E-02	2,16
Q15691	*MAPRE1*	Microtubule-associated protein RP/EB family member 1	3,52E-02	2,11
Q99584	*S100A13*	Protein S100-A13	4,95E-02	2,11
P63167;Q96FJ2	*DYNLL1; DYNLL2*	Dynein light chain 1, cytoplasmic;Dynein light chain 2, cytoplasmic	7,16E-03	2,09
P09486	*SPARC*	SPARC	4,88E-02	2,05
Q96CT7	*CCDC124*	Coiled-coil domain-containing protein 124	8,52E-03	2,04
P12814	*ACTN1*	Alpha-actinin-1	3,40E-02	2,00
Q14240	*EIF4A2*	Eukaryotic initiation factor 4A-II;Eukaryotic initiation factor 4A-II, N-terminally processed	4,00E-02	1,99
P27144	*AK4*	Adenylate kinase 4, mitochondrial	4,44E-02	1,96
O95816	*BAG2*	BAG family molecular chaperone regulator 2	3,23E-02	1,89
Q9BW30	*TPPP3*	Tubulin polymerization-promoting protein family member 3	4,79E-02	1,86
Q8IXJ6	*SIRT2*	NAD-dependent protein deacetylase sirtuin-2	4,40E-02	1,82
P62258	*YWHAE*	14-3-3 protein epsilon	4,01E-02	1,78
Q16181;Q6ZU15	*SEPT7*	Septin-7	1,75E-02	1,73
Q96QR8	*PURB*	Transcriptional activator protein Pur-beta	2,79E-02	1,70
A1L0T0	*ILVBL*	Acetolactate synthase-like protein	2,37E-02	1,69
P35579	*MYH9*	Myosin-9	4,59E-02	1,69
O00233	*PSMD9*	26S proteasome non-ATPase regulatory subunit 9	1,71E-02	1,68
Q9UFN0	*NIPSNAP3A*	Protein NipSnap homolog 3A	3,24E-02	1,63
P62979;P0CG48;P0CG47;P62987	*RPS27A; UBC; UBB; UBA52*	Ubiquitin-40S ribosomal protein S27a;Ubiquitin;40S ribosomal protein S27a;Polyubiquitin-C;Ubiquitin;Polyubiquitin-B;Ubiquitin;Ubiquitin-60S ribosomal protein L40;Ubiquitin;60S ribosomal protein L40	3,79E-02	1,47
P68363;A6NHL2;Q9H853	*TUBA1B*	Tubulin alpha-1B chain	4,60E-02	1,37
P60709;Q9BYX7	*ACTB*	Actin, cytoplasmic 1;Actin, cytoplasmic 1, N-terminally processed	3,82E-02	1,36
P11279	*LAMP1*	Lysosome-associated membrane glycoprotein 1	1,93E-02	-1,37
P84098	*RPL19*	60S ribosomal protein L19	2,27E-02	-1,52
P53985	*SLC16A1*	Monocarboxylate transporter 1	4,98E-02	-1,78

**FIGURE 2:**
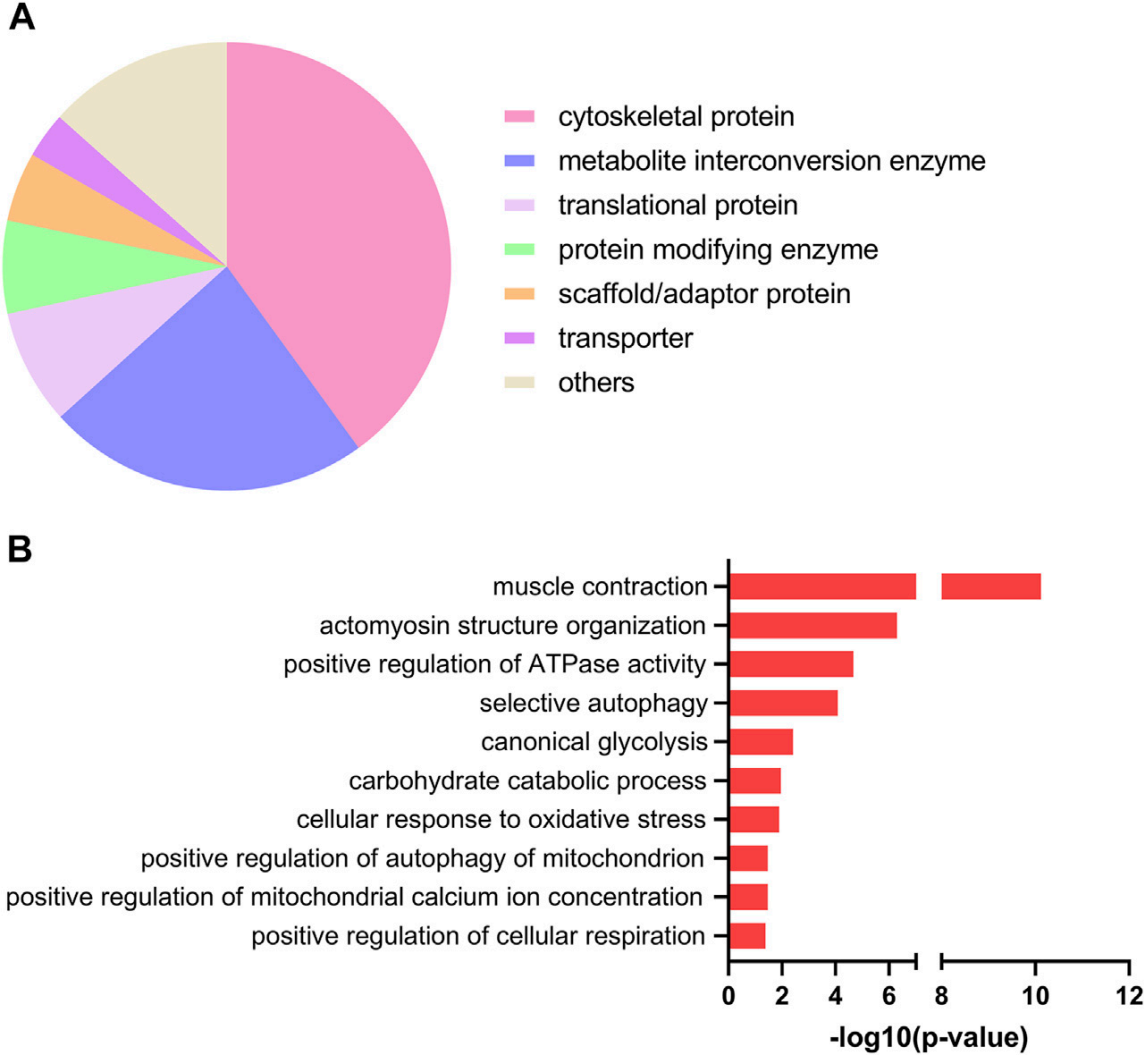
Protein classification and selected gene ontology (GO)-biological processes of upregulated proteins in electrical pulse-stimulated myotubes from young male subjects. **(A)** Protein classes of differentially expressed proteins following electrical pulse stimulation (EPS) protocol 1. **(B)** Selected GO terms among significantly enriched biological processes.

Next, we used the PANTHER resource for classification of the EPS-regulated proteins. As shown in Figure 2A, most regulated proteins were classified as cytoskeletal proteins (43%), metabolite interconversion enzymes (23%) and translational proteins (8%). To further gain insight into the biological roles of the upregulated proteins, we employed Gene Ontology (GO)-based category clustering of biological processes using the biological function enrichment analysis tool EnrichR. Upregulated proteins in myotubes exposed to EPS were significantly enriched in several GO terms, including muscle contraction, positive regulation of ATPase activity, glucose metabolic process, carbohydrate catabolic process, and positive regulation of cellular respiration (Figure 2B). Using human MitoCarta 3.0, we observed that seven out of the 78 upregulated proteins were mitochondria-related proteins. These included succinate dehydrogenase [ubiquinone] iron-sulfur subunit (SDHB), NADH dehydrogenase [ubiquinone] 1 alpha subcomplex subunit 6 (NDUFA6), adenylate kinase 4 (AK4), glutathione S-transferase kappa 1 (GSTK1), protein NipSnap homolog 3A (NIPSNAP3A), histidine triad nucleotide-binding protein 1 (HINT1) and protein deglycase DJ-1 (PARK7) (Table 1).

### 3.3 Differentially Expressed Genes (DEGs) After EPS

**FIGURE 3:**
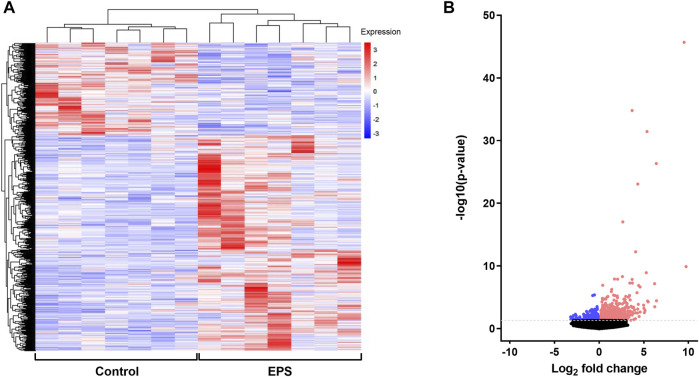
Identification of differentially expressed genes (DEGs) in electrical pulse-stimulated myotubes from young male subjects. **(A)** Heat map showing DEGs after electrical pulse stimulation (EPS protocol 1). Each row represents a gene, whereas each column represents control or EPS-treated myotubes from seven donors. **(B)** Volcano plot of identified transcripts indicating 952 DEGs (665 upregulated and 287 downregulated). The color red on the figures represents upregulated DEGs and blue downregulated DEGs.

To examine the EPS-induced changes at the gene expression level, RNA expression profile of stimulated (EPS protocol 1) and unstimulated myotubes from the same experiments as described in 3.2 were analyzed using RNA seq approach. Differentially expressed genes (DEGs) between the two groups were determined from the level of gene expression using the reads per kilobase per million mapped reads (RPKM). DEGs were hierarchically clustered and visualized as a heat map (Figure 3A). Genes within each condition are clustered together and show the same trends in expression patterns, indicating good reproducibility of EPS treatment. Legend colors used for the clustering information ranging from red to blue indicates gene expression levels from high to low, respectively. A volcano plot was also generated to observe the overall distribution of DEGs between the groups, revealing 665 upregulated and 287 downregulated genes following EPS (Figure 3B). Detailed gene names of DEGs are listed in Supplementary Table 1. Among the upregulated DEGs, 19 genes were annotated by MitoCarta as mitochondria-related genes, whereas 15 mitochondria-related genes were found to be in the list of downregulated DEGs. While comparing the list of significantly regulated proteins and genes obtained from cell proteomics and RNA seq, only enolase 2 (ENO2), a glycolytic enzyme that plays an important role in glycolysis, was upregulated at both protein and mRNA levels.

**FIGURE 4:**
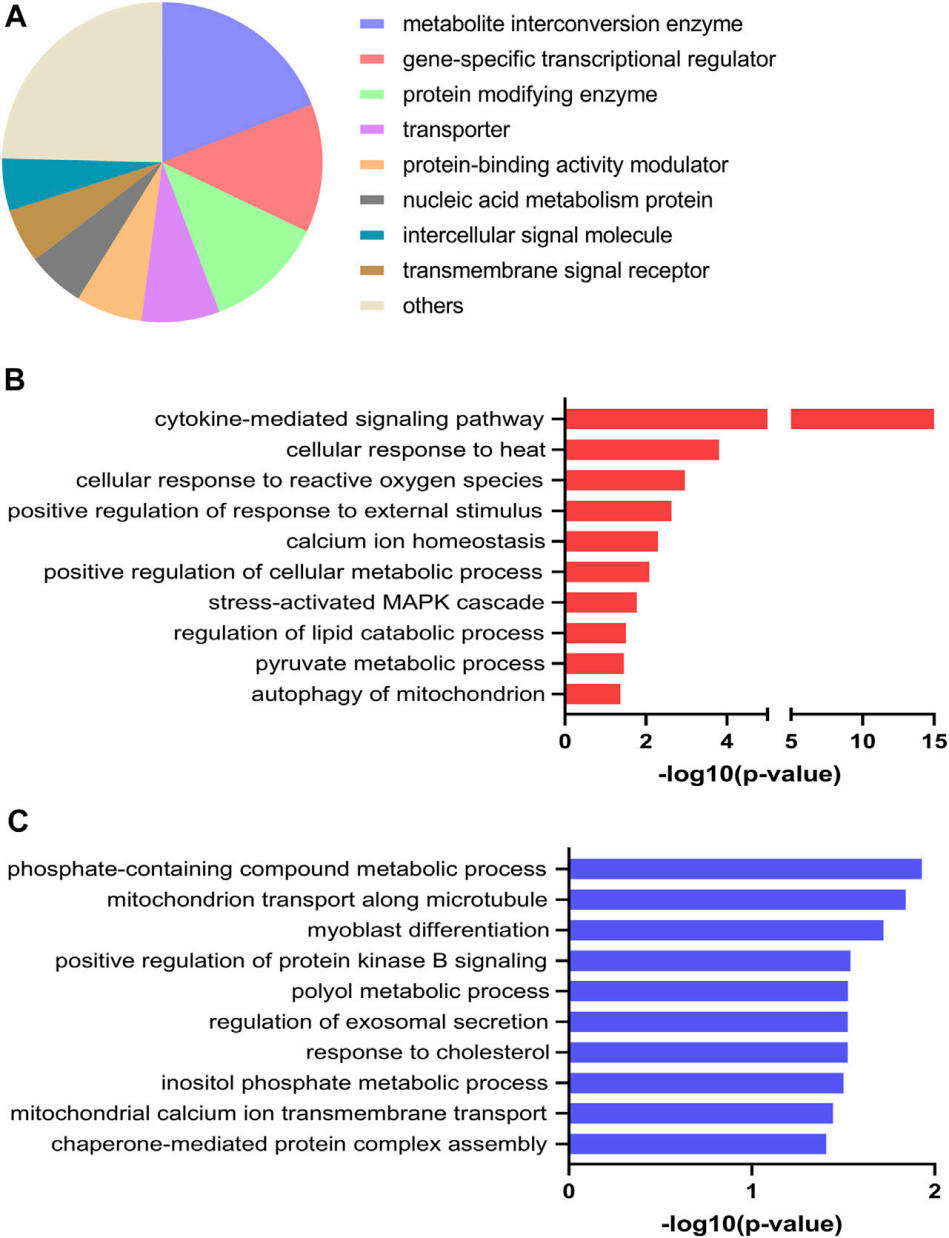
Protein class and selected Gene Ontology (GO) enrichment of differentially expressed genes (DEGs) for electrical pulse-stimulated (EPS protocol 1) versus unstimulated control myotubes from young male subjects. **(A)** Protein classes of DEGs. **(B)** Selected GO biological processes enriched for upregulated DEGs **(C)** Selected GO biological processes enriched for downregulated DEGs.

Both up- and downregulated DEGs were submitted to PANTHER resource and grouped according to protein class. The majority of DEGs were classified as metabolite interconversion enzymes (19%), gene-specific transcriptional regulators (13%), protein modifying enzymes (12%), transporters (8%) and protein-binding activity modulators (7%) (Figure 4A). To evaluate the biological significance of the identified DEGs altered by EPS treatment, GO enrichment analysis was performed using EnrichR analysis tool, where GO Biological Process categories proved to be the most informative. Here, the DEGs represent a wide spectrum of biological processes. Of relevance, the DEGs that were upregulated were significantly enriched in positive regulation of cellular metabolic process, regulation of lipid catabolic process as well as pyruvate metabolic process (Figure 4B). In addition, biological processes involved in cellular response to heat and calcium ion homeostasis were also reflected in this analysis (Figure 4B). With regard to downregulated DEGs, biological processes including mitochondrial calcium ion transmembrane transport and inositol phosphate metabolic process were found to be significantly enriched (Figure 4C). In the GO enrichment analysis of upregulated DEGs stanniocalcin 1 (*STC1*) was identified in the GO term calcium ion homeostasis. Conversely, the mitochondrial calcium uniporter dominant negative β (*MCUB*) (see also Supplementary Table 1) was among the identified downregulated DEGs enriched in mitochondrial calcium ion transport.

### 3.4 Differential Expression of Secreted Proteins After EPS

**FIGURE 5:**
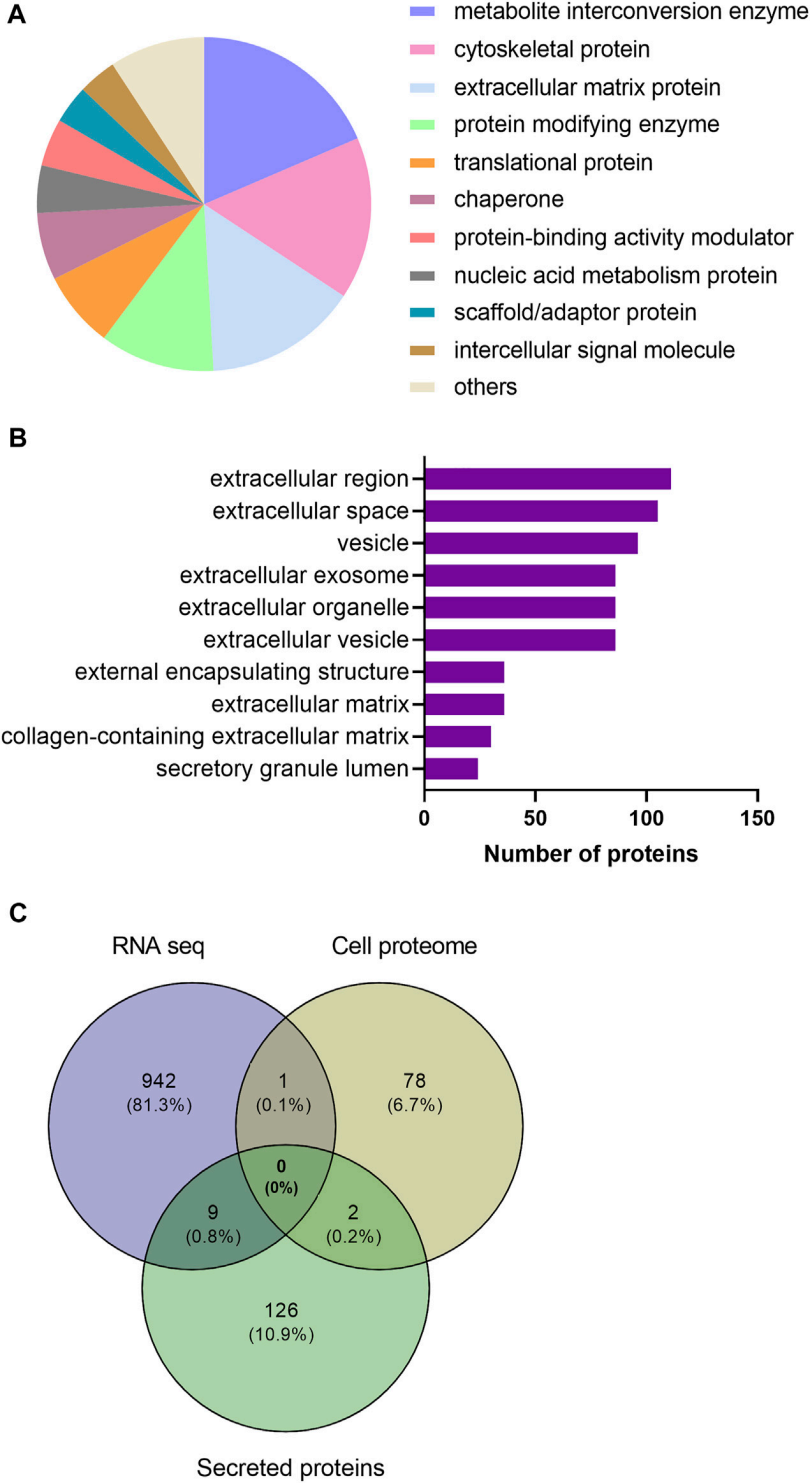
Categorization of significantly regulated secreted proteins after electrical pulse stimulation (EPS protocol 1) of human myotubes obtained from young male subjects based on protein class and Gene Ontology (GO) enrichment analysis. **(A)** Secreted proteins grouped based on PANTHER protein class **(B)** GO cellular component terms overrepresented by EPS-regulated secreted proteins. **(C)** A Venn diagram analysis of regulated proteins and genes identified by proteomic and RNA sequencing (RNA seq) analysis. The intersection of the circles represents overlapping proteins and genes among the cell proteome, secreted proteins and transcriptome from the three datasets.

As myokines released from the skeletal muscle are considered to have implications for the beneficial effects of exercise, we wanted to explore the secreted proteins of myotubes following EPS. From the same EPS experiments (protocol 1, cells from young male subjects) as described in 3.2 and 3.3, serum-free culture media was collected from the myotubes. Proteins in the media were studied by employing a high-resolution LC-MS/MS proteomic analysis. Seven biological replicates per group were included in the analysis, where a total of 1,215 proteins were detected in conditioned medium. Further analysis revealed that extracellular levels of 137 proteins were significantly changed by EPS protocol 1 compared to unstimulated conditions (Supplementary Table 2). Among these proteins in the conditioned medium, 54 were found to be increased, while 83 were reduced by EPS protocol 1. Table 2 shows the top 50 proteins ranked according to fold-change. Proteins significantly changed by EPS protocol 1 in the extracellular fraction were grouped according to their protein class using PANTHER, where the majority were classified as metabolite interconversion enzymes (19%), cytoskeletal proteins (16%) and extracellular matrix proteins (15%) (Figure 5A). Cellular component analysis of proteins that were changed in conditioned media suggested enrichment of the extracellular region, extracellular space, and vesicles (Figure 5B), corroborating that the proteins were indeed myokines regulated by EPS.

**TABLE 2:** List of top 50 differentially secreted proteins induced by electrical pulse stimulation (EPS protocol 1).

Protein IDs	Gene names	Protein names	*p*-value	Fold Change
P31431	*SDC4*	Syndecan-4	1,95E-03	6,0
P48307	*TFPI2*	Tissue factor pathway inhibitor 2	3,58E-05	5,9
P98066	*TNFAIP6*	Tumor necrosis factor-inducible gene 6 protein	3,19E-02	5,5
P22105	*TNXB*	Tenascin-X	1,83E-02	4,0
Q92626	*PXDN*	Peroxidasin homolog	6,76E-05	3,8
P03956	*MMP1*	Interstitial collagenase;22 kDa interstitial collagenase;27 kDa interstitial collagenase	1,40E-02	3,7
P10145	*CXCL8*	Interleukin-8;MDNCF-a;Interleukin-8;IL-8(5-77);IL-8(6-77);IL-8(7-77);IL-8(8-77);IL-8(9-77)	5,72E-03	3,7
P42830	*CXCL5*	C-X-C motif chemokine 5;ENA-78(8-78);ENA-78(9-78)	4,34E-02	3,6
P29279	*CTGF*	Connective tissue growth factor	1,36E-02	3,4
P09341	*CXCL1*	Growth-regulated alpha protein;GRO-alpha(4-73);GRO-alpha(5-73);GRO-alpha(6-73)	3,94E-02	3,2
P19876	*CXCL3*	C-X-C motif chemokine 3;GRO-gamma(5-73)	3,94E-02	3,2
P19875	*CXCL2*	C-X-C motif chemokine 2;GRO-beta(5-73)	3,94E-02	3,2
Q9BS26	*ERP44*	Endoplasmic reticulum resident protein 44	8,20E-03	3,1
Q14767	*LTBP2*	Latent-transforming growth factor beta-binding protein 2	4,40E-06	2,9
Q76M96	*CCDC80*	Coiled-coil domain-containing protein 80	1,38E-04	2,3
Q05707	*COL14A1*	Collagen alpha-1(XIV) chain	1,10E-02	2,3
Q15582	*TGFBI*	Transforming growth factor-beta-induced protein ig-h3	6,20E-04	2,3
O95967	*EFEMP2*	EGF-containing fibulin-like extracellular matrix protein 2	1,40E-03	2,2
O95633	*FSTL3*	Follistatin-related protein 3	2,02E-02	2,1
P15531	*NME1*	Nucleoside diphosphate kinase A	4,92E-02	2,1
P10124	*SRGN*	Serglycin	4,27E-02	2,0
Q13177	*PAK2*	Serine/threonine-protein kinase PAK 2;PAK-2p27;PAK-2p34	3,79E-02	1,9
P02751	*FN1*	Fibronectin;Anastellin;Ugl-Y1;Ugl-Y2;Ugl-Y3	1,55E-05	1,9
P98160	*HSPG2*	Basement membrane-specific heparan sulfate proteoglycan core protein;Endorepellin;LG3 peptide	2,96E-03	1,9
P08254	*MMP3*	Stromelysin-1	1,81E-02	1,9
P31942	*HNRNPH3*	Heterogeneous nuclear ribonucleoprotein H3	3,74E-02	1,8
P00441	*SOD1*	Superoxide dismutase [Cu-Zn]	6,74E-03	−3,0
Q9UKY7	*CDV3*	Protein CDV3 homolog	3,50E-02	−3,1
P11766	*ADH5*	Alcohol dehydrogenase class-3	1,67E-02	−3,2
P09972	*ALDOC*	Fructose-bisphosphate aldolase C	5,15E-03	−3,3
P05155	*SERPING1*	Plasma protease C1 inhibitor	3,77E-02	−3,3
O75368	*SH3BGRL*	SH3 domain-binding glutamic acid-rich-like protein	8,04E-03	−3,4
P63220	*RPS21*	40S ribosomal protein S21	1,69E-03	−3,4
P05387	*RPLP2*	60S acidic ribosomal protein P2	3,19E-02	−3,5
P09382	*LGALS1*	Galectin-1	9,35E-03	−3,5
P32119	*PRDX2*	Peroxiredoxin-2	2,80E-02	−3,5
P07437	*TUBB*	Tubulin beta chain	4,21E-02	−3,6
Q8NBS9	*TXNDC5*	Thioredoxin domain-containing protein 5	1,21E-02	−3,6
P60903	*S100A10*	Protein S100-A10	4,44E-02	−3,7
P07686	*HEXB*	Beta-hexosaminidase subunit beta;Beta-hexosaminidase subunit beta chain B;Beta-hexosaminidase subunit beta chain A	1,65E-02	−3,7
P29692	*EEF1D*	Elongation factor 1-delta	3,27E-03	−3,8
P22314	*UBA1*	Ubiquitin-like modifier-activating enzyme 1	1,82E-02	−3,8
P08865	*RPSA*	40S ribosomal protein SA	5,22E-03	−4,0
P05388	*RPLP0;*	60S acidic ribosomal protein P0	1,08E-02	−4,2
Q8NHW5	*RPLP0P6*	60S acidic ribosomal protein P0-like	1,08E-02	−4,2
Q9NQC3	*RTN4*	Reticulon-4	1,41E-02	−4,5
O75083	*WDR1*	WD repeat-containing protein 1	1,32E-03	−4,7
Q9H299	*SH3BGRL3*	SH3 domain-binding glutamic acid-rich-like protein 3	1,81E-02	−4,8
P60033	*CD81*	CD81 antigen	5,59E-03	−5,2
Q3ZCM7	*TUBB8*	Tubulin beta-8 chain	5,49E-03	−6,3
Q01105	*SET*	Protein SET	2,33E-03	−6,3
P0DME0	*SETSIP*	Protein SETSIP	2,33E-03	−6,3
Q5VTE0	*EEF1A1P5*	Putative elongation factor 1-alpha-like 3	4,90E-03	−12,8
P68104	*EEF1A1*	Elongation factor 1-alpha 1	4,90E-03	−12,8
Q05639	*EEF1A2*	Elongation factor 1-alpha 2	4,90E-03	−12,8
P04406	*GAPDH*	Glyceraldehyde-3-phosphate dehydrogenase	8,27E-04	−14,5

Finally, comparison between significantly regulated genes, cellular proteins, and secreted proteins from the same experiments using myotubes from young male subjects and EPS protocol 1 was performed using Venn diagram analysis (Figure 5C). We found no shared gene/protein within all the three datasets. Nonetheless, in addition to ENO2 that was found to overlap among the data from RNA seq and cell proteomics, two proteins, actin alpha cardiac muscle 1 (ACTC1) and 14-3-3 epsilon (ε) protein (YWHAE), were common among regulated proteins found in the cell proteome and secreted proteins. Nine proteins were commonly shared between the secreted proteins and the transcriptome data (Figure 5C). These are the C-X-C Motif chemokine ligand 1, 5 and 8 (CXCL1, CXCL5, CXCL8), matrix metallopeptidase-1 and -3 (MMP1 and MMP3), tissue factor pathway inhibitor 2 (TFPI2), brain acid soluble protein 1 (BASP1), TNF alpha induced protein 6 (TNFAIP6), and ribosomal protein lateral stalk subunit P2 (RPLP2).

### 3.5 Shared Regulated Transcripts Between EPS Protocol 1 in Cells From Young Male Subjects and EPS Protocol 2 in Cells From Middle-Aged Women

**FIGURE 6:**
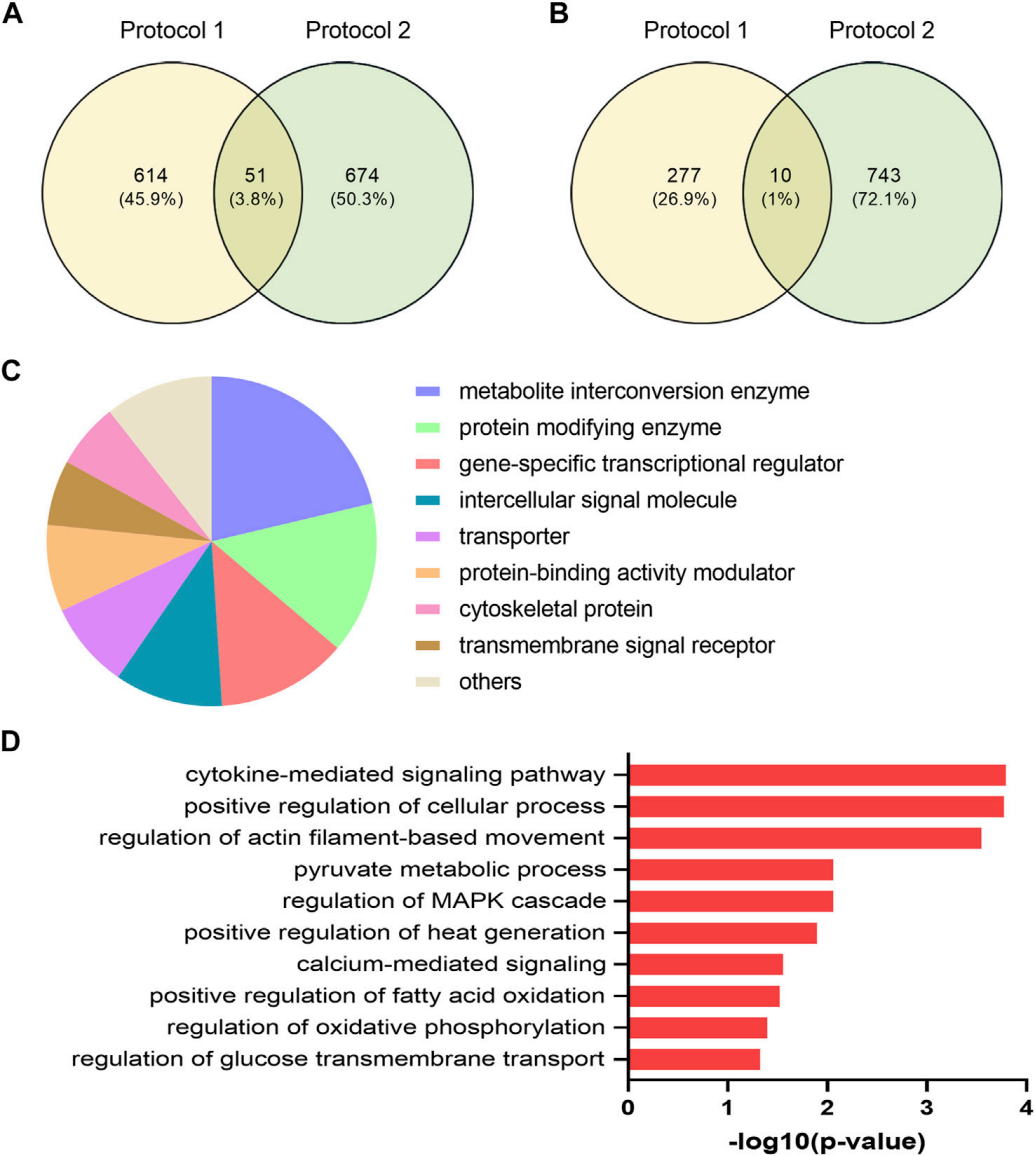
Functional classification and selected Gene Ontology (GO) enrichment of shared differentially expressed genes (DEGs) in myotubes from young male subjects and middle-aged women exposed to EPS protocol 1 and 2, respectively. Common upregulated **(A)** and downregulated **(B)** transcripts in both EPS protocols showed by Venn diagram **(C)** Protein classes corresponding to all commonly shared DEGs **(D)** Selected GO Biological Process showing GO terms significantly enriched in the upregulated DEGs.

Different EPS protocols are used in the literature (reviewed in (Nikolić et al., 2017)), as well as in our laboratory (Nikolić et al., 2012; Feng et al., 2015; Løvsletten et al., 2020). Cultured human myotubes are also know to retain several characteristics of the donor (Aas et al., 2013). Thus, to verify common effects of two different protocols applied on two different donor groups, we compared DEGs from the young male subjects (BMI 23.8 ± 0.8 kg/m^2^, age 23.4 ± 0.9 years) exposed to protocol 1 (Supplementary Table 1) with mRNA expression profile of myotubes from middle-aged women (BMI 24.3 ± 1.7 kg/m^2^, age 49.8 ± 2.2 years) submitted to EPS protocol 2 (i.e. 2 ms, 30 V, and 1 Hz for 48 h), assessed by microarray analysis. Although the majority of the DEGs are different between these lists, we found a total of 61 DEGs that were commonly shared and showed the same expression pattern in both groups and EPS models. Out of these 61 DEGs, 51 genes were upregulated (Figure 6A) and 10 genes were downregulated (Figure 6B). These commonly shared DEGs were grouped as metabolite interconversion enzyme (21%), protein modifying enzyme (15%), gene-specific transcriptional regulator (13%), intercellular signal molecule (11%), and transporter (9%) (Figure 6C) according to PANTHER protein class classification. Moreover, among the list of overlapping transcripts, one mitochondrial gene, peptidylprolyl isomerase F (*PPIF*) involved in mitochondrial bioenergetics, was found to be upregulated in both.

A number of GO Biological Processes for the upregulated genes were further retrieved by performing GO-enrichment analysis using EnrichR analysis tool. Of GO biological processes related to metabolism, pyruvate metabolic process, regulation of glucose transmembrane transport, positive regulation of fatty acid oxidation, and regulation of oxidative phosphorylation were significantly enriched (Figure 6D). Of particular interest was the nuclear receptor subfamily 4 group A member 3 (*NR4A3*) involved in most of the aforementioned GO terms. Enrichment analysis of downregulated DEGs was not carried out as the number of downregulated DEGs was limited (10 genes). The details of these shared transcripts are summarized in Table 3.

**TABLE 3:** Regulated overlapping genes between EPS-treated myotubes from young male subjects and middle-aged women obtained from RNA sequencing and microarray platforms.

Gene ID	Gene name	Gene description	Fold change EPS 24 h (protocol 1)	Fold change EPS 48 h (protocol 2)
ENSG00000168685	*IL7R*	interleukin 7 receptor	2,17	1,78
ENSG00000100292	*HMOX1*	heme oxygenase 1	2,57	1,77
ENSG00000119714	*GPR68*	G protein-coupled receptor 68	1,78	1,65
ENSG00000064195	*DLX3*	distal-less homeobox 3	1,83	1,61
ENSG00000105825	*TFPI2*	tissue factor pathway inhibitor 2	2,66	1,60
ENSG00000001084	*GCLC*	glutamate-cysteine ligase catalytic subunit	1,24	1,59
ENSG00000115919	*KYNU*	kynureninase	3,21	1,44
ENSG00000196611	*MMP1*	matrix metallopeptidase 1	3,31	1,44
ENSG00000159167	*STC1*	stanniocalcin 1	4,13	1,40
ENSG00000260549	*MT1L*	metallothionein 1L, pseudogene	4,21	1,38
ENSG00000135678	*CPM*	carboxypeptidase M	1,76	1,37
ENSG00000130066	*SAT1*	spermidine/spermine N1-acetyltransferase 1	1,55	1,35
ENSG00000091129	*NRCAM*	neuronal cell adhesion molecule	1,48	1,30
ENSG00000162493	*PDPN*	podoplanin	1,68	1,30
ENSG00000029153	*ARNTL2*	aryl hydrocarbon receptor nuclear translocator like 2	1,56	1,29
ENSG00000198018	*ENTPD7*	ectonucleoside triphosphate diphosphohydrolase 7	1,42	1,28
ENSG00000128342	*LIF*	Leukemia inhibitory factor, interleukin 6 family cytokine	3,05	1,28
ENSG00000138685	*FGF2*	fibroblast growth factor 2	1,91	1,26
ENSG00000153162	*BMP6*	bone morphogenetic protein 6	2,42	1,26
ENSG00000197013	*ZNF429*	zinc finger protein 429	1,28	1,25
ENSG00000073756	*PTGS2*	prostaglandin-endoperoxide synthase 2	5,73	1,25
ENSG00000185947	*ZNF267*	zinc finger protein 267	1,56	1,24
ENSG00000131737	*KRT34*	keratin 34	3,35	1,24
ENSG00000065833	*ME1*	malic enzyme 1	1,21	1,23
ENSG00000170075	*GPR37L1*	G protein-coupled receptor 37 like 1	9,62	1,23
ENSG00000143995	*MEIS1*	Meis homeobox 1	2,88	1,22
ENSG00000130254	*SAFB2*	scaffold attachment factor B2	1,13	1,22
ENSG00000183735	*TBK1*	TANK binding kinase 1	1,19	1,21
ENSG00000137776	*SLTM*	SAFB like transcription modulator	1,22	1,21
ENSG00000163681	*SLMAP*	sarcolemma associated protein	1,10	1,20
ENSG00000114573	*ATP6V1A*	ATPase H+ transporting V1 subunit A	1,17	1,20
ENSG00000136160	*EDNRB*	endothelin receptor type B	2,03	1,20
ENSG00000132436	*FIGNL1*	fidgetin like 1	1,28	1,20
ENSG00000117228	*GBP1*	guanylate binding protein 1	1,39	1,20
ENSG00000118193	*KIF14*	kinesin family member 14	3,69	1,18
ENSG00000124789	*NUP153*	nucleoporin 153	1,18	1,18
ENSG00000119508	*NR4A3*	nuclear receptor subfamily 4 group A member 3	2,00	1,18
ENSG00000113522	*RAD50*	RAD50 double strand break repair protein	1,23	1,17
ENSG00000169429	*CXCL8*	C-X-C motif chemokine ligand 8	12,97	1,17
ENSG00000177409	*SAMD9L*	sterile alpha motif domain containing 9 like	1,36	1,17
ENSG00000108179	*PPIF*	peptidylprolyl isomerase F	1,44	1,16
ENSG00000144802	*NFKBIZ*	NFKB inhibitor zeta	4,17	1,16
ENSG00000109436	*TBC1D9*	TBC1 domain family member 9	1,40	1,15
ENSG00000205362	*MT1A*	metallothionein 1A	13,17	1,15
ENSG00000111885	*MAN1A1*	mannosidase alpha class 1A member 1	1,91	1,15
ENSG00000116266	*STXBP3*	syntaxin binding protein 3	1,09	1,14
ENSG00000104419	*NDRG1*	N-myc downstream regulated 1	1,52	1,13
ENSG00000008294	*SPAG9*	sperm associated antigen 9	1,20	1,12
ENSG00000168209	*DDIT4*	DNA damage inducible transcript 4	1,35	1,12
ENSG00000164283	*ESM1*	endothelial cell specific molecule 1	2,65	1,12
ENSG00000085662	*AKR1B1*	aldo-keto reductase family 1 member B	1,73	1,10
ENSG00000106484	*MEST*	mesoderm specific transcript	−1,64	−1,11
ENSG00000136114	*THSD1*	thrombospondin type 1 domain containing 1	−1,43	−1,12
ENSG00000205208	*C4orf46*	chromosome 4 open reading frame 46	−1,36	−1,14
ENSG00000133243	*BTBD2*	BTB domain containing 2	−1,21	−1,15
ENSG00000184584	*TMEM173*	transmembrane protein 173	−1,20	−1,17
ENSG00000100605	*ITPK1*	inositol−tetrakisphosphate 1-kinase	−1,15	−1,20
ENSG00000124193	*SRSF6*	serine and arginine rich splicing factor 6	−1,11	-1,20
ENSG00000185133	*INPP5J*	inositol polyphosphate-5-phosphatase J	−2,43	−1,22
ENSG00000126603	*GLIS2*	GLIS family zinc finger 2	−1,43	−1,23
ENSG00000130592	*LSP1*	lymphocyte specific protein 1	−1,81	−1,24

### 3.6 Validation of Differentially Regulated Myokines After EPS

**FIGURE 7:**
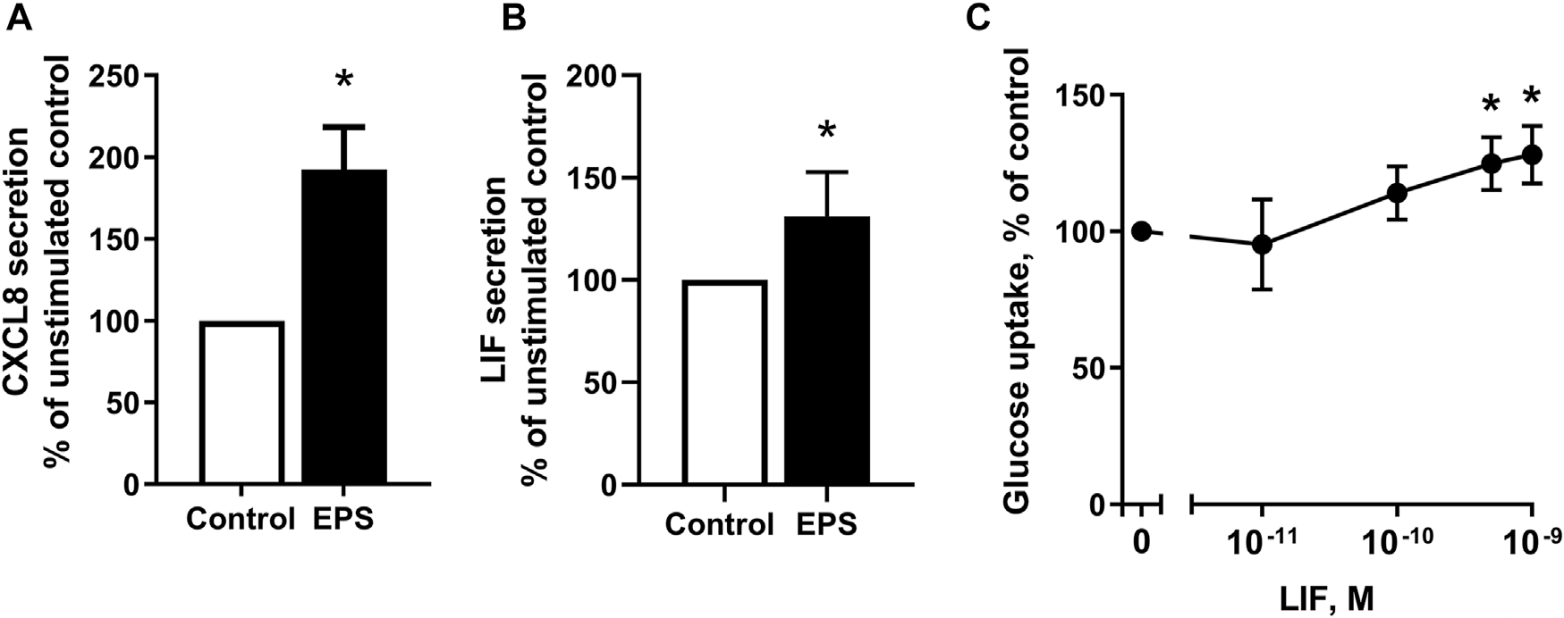
Detection of myokines in conditioned media following electrical pulse stimulation (EPS protocol 2) for 48 h and effect of leukemia inhibitory factor (LIF) on glucose uptake. **(A,B)** Human myotubes from middle-aged women and men were cultured and EPS protocol 2 was applied before conditioned media were harvested. The concentrations of CXCL8 **(A)** and LIF **(B)** in media were determined by ELISA and multiplex immunoassay, respectively. Data are expressed as mean ± SEM, normalized to media from non-treated control cells (absolute values: IL-8; 3 ± 0.07 pM. and LIF; 1 ± 0.03 pM.) **(C)** Myotubes from the same subjects were incubated in buffer with 2-[^3^H(G)]deoxy-d-glucose (1 μCi/ml, 10 μM) and various concentrations of LIF (0.01–1 nM) and uptake of deoxyglucose was measured for 15 min as described in Materials and Methods. All data are shown as normalized to unstimulated control cells from six (*n* = 6, **(A,B)** and nine (*n* = 9, **(C)** individual experiments. *p < 0.05 vs. control.

We observed that myotubes from young male subjects (BMI 23.8 (±0.8) kg/m^2^, age 23.4 ± 0.9) exposed to EPS protocol 1 had significantly higher mRNA expression of the myokines CXCL8 and LIF (Supplementary Table 1) compared to control cells, as well as higher level of secreted CXCL8 (Table 2). Microarray data of cells obtained from middle-aged women (24.3 (±1.7) kg/m^2^, age 49.8 ± 2.2) following EPS protocol 2 also show higher mRNA expression of CXCL8 and LIF (Table 3). To elaborate on these results, ELISA or bead-based multiplex assay was employed to investigate whether LIF and CXCL8 proteins were increased in the cultured media collected from myotubes of middle-aged women and men (BMI 29.7 ± 2.3 kg/m^2^, age 50.4 ± 1.7 years) after the application of EPS protocol 2. The results showed that concentrations of both CXCL8 (Figure 7A) and LIF (Figure 7B) were elevated in media obtained from EPS-treated myotubes compared to control cells.

Although myokines are supposed to be implicated in the metabolic and physiologic responses to exercise in various organs, including skeletal muscle itself, only few myokines have been linked to a specific biological function. As our *in vitro* experiments provide evidence that LIF was among the secreted proteins induced by EPS, we wanted to examine whether stimulation of myotubes with LIF could affect glucose uptake. Myotubes established from middle-aged women and men were treated with LIF 0.01–1 nM for 15 min. Here, we show that deoxyglucose uptake was significantly increased in cultured myotubes (Figure 7C).

## 4 Discussion

Given the numerous beneficial changes that occur during exercise, understanding the cellular and molecular mechanisms underlying the complex physiological responses to exercise is a long-standing goal. In this study, we used two different *in vitro* models of chronic exercise to investigate mechanisms involved in metabolic adaptations in human skeletal muscle cells. We showed that application of EPS protocol 1 (2 ms, 10 V and 0.1 Hz for 24 h) to myotubes derived from young male subjects increased oxidation of both glucose and oleic acid, as well as enhanced the uptake of oleic acid compared to untreated control cells. We also characterized the EPS-regulated cell proteome, transcriptome, and secreted proteins to uncover the biological processes contributing to the improved metabolism in myotubes from these young male subjects. The cell proteome analysis showed 81 proteins (78 upregulated and 3 downregulated) that were differentially regulated in myotubes submitted to EPS. High-throughput sequencing data revealed a robust change in transcript expression profile, where 952 genes (665 upregulated and 287 downregulated) were differentially expressed by EPS. Interestingly, among these DEGs, we found 61 genes to be commonly shared when comparing gene expression data obtained from myotubes derived from young male subjects exposed to EPS protocol 1 and from myotubes derived from middle-aged women exposed to protocol 2 (i.e., 2 ms, 30 V and 1 Hz for 48 h. Finally, investigation of secreted proteins in conditioned media using proteomic approach resulted in the identification of 137 proteins (54 upregulated and 83 downregulated) as a response to EPS protocol 1.

Over the years, a number of different EPS protocols have been used as *in vitro* exercise models to explore mechanisms underlying exercise-induced adaptations of skeletal muscle (Lambernd et al., 2012; Nikolić et al., 2012; Scheler et al., 2013; Feng et al., 2015; Furuichi et al., 2018; Løvsletten et al., 2020; Marš et al., 2020; Tamura et al., 2020). With regards to energy metabolism, we have previously reported increases in glucose and fatty acid metabolism in cultured skeletal muscle cells from middle-aged women and men following the application of EPS protocol 2 (Nikolić et al., 2012). In line with this notion, we observed similar metabolic responses when we exposed cultured myotubes obtained from young male subjects to EPS protocol 1. These findings are indeed in accordance with the changes in the metabolic properties of skeletal muscle mediated by exercise *in vivo* (Egan and Zierath, 2013), highlighting the usefulness of this model in recapitulating mechanisms behind metabolic alterations related to exercise.

Although substantial progress has been made in our understanding of distinct biological mechanisms that occur in response to exercise, full comprehension of the underlying cellular and molecular mechanisms involved in the adaptive response of skeletal muscle to contractile activity remains to be determined. Recent advances in omics technologies and their growing application have facilitated the progress to unravel the molecular landscape of exercise biology. By taking advantage of these omics-based measurements, we have identified a number of differentially regulated genes and proteins that may have implications on biological processes associated with metabolic alterations during muscle contraction. However, despite resembling several features of exercise *in vivo*, not all changes seen following whole-body exercise (see e.g. (Egan and Zierath, 2013; Flück, 2006; Hoppeler et al., 2011; Pedersen and Febbraio, 2012)) are reproduced in human myotubes exposed to EPS, which might be, at least in part, due to the lack of circulating hormones and other *in vivo* microenvironment alterations that typically occur during exercise *in vivo*.

Skeletal muscle adaptations are mediated by a network of molecular and metabolic pathways that are triggered by muscle contraction (Flück and Hoppeler, 2003; Ferraro et al., 2014). Cytoskeletal networks and their associated regulatory molecules are essential for efficient muscle contraction during exercise (Clark et al., 2002). Therefore, it was noteworthy that the majority of significantly regulated proteins obtained from cell proteome analysis in our study were classified as cytoskeletal proteins. These proteins are important in providing mechanical stability to the surface membrane during contraction and allow for transmission of forces generated by the actin-myosin cross-bridge cycle (Lieber et al., 2002). Moreover, it has been suggested that maintaining the structural integrity of the contractile apparatus is a highly energy consuming process, which further induces metabolic alterations to meet the ATP demand (DeWane et al., 2021). The availability of ATP is also crucial for skeletal muscle contractile activity. As the total amount of ATP stored within the cells is relatively small to sustain longer periods of contraction, metabolic pathways such as glycolysis and oxidative metabolism must therefore be activated to ensure sufficient ATP production (Hargreaves and Spriet, 2020). Indeed, we observed the mitochondrial-related proteins, such as AK4, SDHB, and NDUFA6 to be upregulated in cells from young male subjects exposed to EPS protocol 1. It has been reported that AK4 and SDHB are involved in the regulation of both mitochondrial TCA cycle and oxidative phosphorylation (Rutter et al., 2010; Klepinin et al., 2020), whereas NDUFA6, which is a subunit of the largest and most complex enzyme in the oxidative phosphorylation system (complex I), participate in mitochondrial catalytic activity (Angerer et al., 2014). Thus, upregulation of these proteins following EPS protocol 1 could mediate an increase in ATP production, which might also explain the improved oxidative capacity in EPS exposed myotubes from young male subjects. Consistent with these notions, the results from GO function annotation of cell proteome revealed that upregulated proteins in these myotubes were enriched in muscle contraction, actomyosin structure organization, canonical glycolysis, positive regulation of cellular respiration, and ATPase activity.

In addition to the aforementioned biological processes, cellular response to oxidative stress and selective autophagy, including positive regulation of autophagy of mitochondrion were reflected in this analysis. Indeed, oxidative stress and autophagy are known to be evoked by regular exercise (Powers and Jackson, 2008; Ferraro et al., 2014; Vainshtein and Hood, 2015; Powers et al., 2020). Both oxidative stress and autophagy are processes with ambivalent effects as they can be detrimental and beneficial depending on their balance. Although, the consequences of exercise-induced oxidative stress is still debated, emerging evidences suggest that production of reactive oxygen species (ROS) during exercise plays an important role in the activation of nuclear factor-kappa B (NF-κB) and peroxisome proliferator-activated receptor gamma coactivator 1-alpha (PGC-1α) to promote synthesis of antioxidants and mitochondrial biogenesis (Powers et al., 2011; Powers et al., 2020). Further, the mitochondrial proteins GSTK1 and PARK7, which are found to be upregulated in myotubes of young male subjects exposed to EPS protocol 1 have been implicated to play a role in protecting the mitochondria against oxidative stress (Raza, 2011; Mencke et al., 2021). Thus, the available evidence suggests that ROS production as a result of exercise-mediated oxidative stress could be a requirement for skeletal muscle metabolic adaptations, and that compensatory mechanisms are induced to maintain the redox balance, protecting the cell against deleterious effects of contraction-mediated oxidative stress. Moreover, autophagy, which is a self-degradative process essential for providing new source of energy and disposal of misfolded proteins and damaged organelles including mitochondria (mitophagy) (Glick et al., 2010; Roberts and Markby, 2021), has been suggested to be initiated by ROS and AMPK in exercising muscle (Glick et al., 2010; Hardie, 2011; Ferraro et al., 2014; Vainshtein and Hood, 2015). It is known that the raise in AMP:ATP-ratio during *in vivo* exercise and *ex vivo* skeletal muscle contraction activates AMPK (Hardie and SakamotoAMPK, 2006; Kjøbsted et al., 2018). In accordance to these findings, we observed that myotubes from young male subjects exposed to EPS protocol 1 had higher AMPK activity compared to control cells. With respect to energy metabolism, the activation of AMPK promotes enhanced glucose and fatty acid metabolism by inhibiting energy-consuming pathways and activating catabolic pathways, thereby ensuring that the ATP levels are restored (Hardie and SakamotoAMPK, 2006; Hardie, 2011; Kjøbsted et al., 2018). In addition, previous studies have demonstrated that induction of autophagy and mitochondria turnover following exercise could be coordinated by PGC-1α (Vainshtein et al., 2015a; Vainshtein et al., 2015b). The data obtained in the current study thus support the possibility that increased AMPK activity as a response to muscle contraction, independently of hormonal influence, could at least in part be responsible for the improved substrate metabolism through the induction of autophagy/mitophagy in a similar manner as exercise *in vivo*.

In accordance with the results from cell proteome, GO function annotation of upregulated DEGs from RNA seq analysis of cells from young male subjects exposed to protocol 1 were also enriched in many of the same GO terms, including cellular response to reactive oxygen species, autophagy of mitochondrion, cellular response to heat, and calcium ion homeostasis. Indeed, a rapid release and re-sequestration of Ca^2+^ from sarcoplasmic reticulum to cytosol, a process which is tightly regulated, is essential for muscle contraction as well as other cellular events (Berchtold et al., 2000). Over the years, it has become increasingly evident that mitochondria plays a key role in regulation of intracellular calcium homeostasis (Rizzuto et al., 2012). Upon contraction, the elevated level of cytosolic Ca^2+^ drives mitochondrial uptake and sequestration of Ca^2+^, which activates intrinsic functions including ATP production by oxidative phosphorylation (Griffiths and Rutter, 2009; Rizzuto et al., 2012). Moreover, heat is generated through both myosin-mediated ATP hydrolysis and Ca^2+^ transport driven by sarcoendoplasmic reticulum calcium ATPase (SERCA) during muscle contraction (Periasamy et al., 2017), indicating the link between Ca^2+^ homeostasis and heat production.

A close look into the DEGs involved in maintaining Ca^2+^ homeostasis, we found stanniocalcin-1 (*STC-1*) to be upregulated in myotubes established from both young male subjects and middle-aged women treated with two different EPS protocols. STC-1 is a Ca^2+^-regulating glycosylated peptide hormone associated with a variety of biological processes including oxidative stress, cell proliferation, apoptosis, and metabolism (Joshi, 2020). Using intact, respiring mitochondria isolated from rat muscle, Ellard et al. (2007) demonstrated that *STC-1* targeted the mitochondrial matrix to stimulate electron transport, uncouple oxidative phosphorylation, and enhance mitochondrial calcium accumulation (Ellard et al., 2007). This suggests that the metabolic alterations observed in EPS-treated myotubes could, at least in part, be mediated by Ca^2+^-signaling through the involvement of STC-1. Our analysis also showed that mitochondrial calcium ion transmembrane transport was enriched in downregulated DEGs. Among the genes involved in this process, the mitochondrial calcium uniporter dominant negative β (*MCUB*) was observed to be downregulated only in myotubes from young males subjected to EPS protocol 1. Although the role of MCUB is just starting to emerge, incorporation of MCBU to mitochondrial calcium uniporter channel (mtCU) appeared to decrease mtCU-dependent Ca^2+^ uptake which can lead to diminished contractile function and impaired mitochondrial energetics (Lambert et al., 2020). Thus, reduced expression of *MCBU* could have beneficial metabolic outcome in exercising muscle.

Along with intrinsic exercise-related factors including intensity, duration and mode of exercise, substrate utilization in skeletal muscle can be influenced by external factors such as age, sex, and body composition (Egan and Zierath, 2013). In the present study, we used different age groups, EPS protocols, and transcriptomic platforms to determine gene expression profile changes that can occur in skeletal muscle cells undergone different modalities of chronic “exercise” interventions. We found 61 (51 upregulated and 10 downregulated) DEGs to be commonly shared between EPS protocol 1-treated myotubes from young male subjects and EPS protocol 2-exposed middle-aged women, assessed by RNA seq and microarray analysis, respectively. Thus, the shared regulation of genes with both EPS protocols exerted on different donor groups indicates that these genes might be key contributing factors for skeletal muscle metabolic adaptations to muscle contraction regardless of age, gender, or type of contraction protocols.

Furthermore, enrichment analysis of commonly upregulated DEGs in both group also displayed biological processes involved in glucose and fatty acid metabolism including regulation of glucose transmembrane transport, pyruvate metabolic process, fatty acid oxidation, and regulation of oxidative phosphorylation. Among the genes, we found the nuclear receptor subfamily 4 group A member 3 (*NR4A3*) to be of particular interest as it was involved in almost all of the aforementioned metabolic processes. As one of the three members in NR4A family, NR4A3 (NOR-1) can exert different biological functions by directly or indirectly affecting the activity of mitochondria, as well as by interacting with other coactivators in nucleus to regulate gene transcription (Zhang et al., 2020). Increased expression of NR4A3 following exercise seems to be initiated by Ca^2+^ signaling (Pearen and Muscat, 2018), which is a fundamental driver of skeletal muscle contraction. Indeed, the upregulation of *NR4A3* following EPS was in accordance with previously reported transcriptomic data using a meta-analysis of currently available exercise response datasets (Pillon et al., 2020). In this meta-analysis from 66 published datasets, the authors identified NR4A3 to be an important regulator of exercise adaptations independent of exercise modality, which support the upregulation of NR4A3 in both of our *in vitro* exercise protocols. Several studies have demonstrated the role of NR4A3 in regulating skeletal muscle energy homeostasis, where muscle-specific overexpression of NR4A3 in mice has been implicated in mediating glycolysis, increased β-oxidation, increased tricarboxylic acid cycle flux, increased mitochondrial density, autophagy, and switch toward a more oxidative fiber type (Pearen et al., 2008; Pearen et al., 2013; Goode et al., 2016; Pearen and Muscat, 2018). Collectively, these reports clearly establish NR4A3 as a pleiotropic modulator of contraction-induced adaptations, suggesting that the improved glucose and fatty acid metabolism in myotubes after EPS could be induced by increased *NR4A3* activity.

Available evidence shows that exercise also exerts physiological alterations in the immune system, which is largely mediated by cytokine signaling (Docherty et al., 2022). Notably, both chronic EPS protocols have shown to elevate the transcriptional expression of several cytokines including the CXCL8/IL-8 and LIF which we found to be enriched in cytokine-mediated signaling pathway. These findings were in accordance with previous literature reporting an increase in IL-8 and LIF following exercise, and are thus classified as myokines (Pedersen and Febbraio, 2012; Das et al., 2020). Moreover, it is known that muscle contraction is a major stimulator of myokine secretion. Myokines are implicated in paracrine, endocrine, or autocrine effects to maintain tissue homeostasis (Pedersen and Febbraio, 2012; Das et al., 2020). However, there is limited information available on the vast majority of myokines defining their specific impact on overall metabolism. Several studies have demonstrated that, in addition to their inflammatory responses in various tissues, cytokines of the IL-6 family also have a role to play in muscle glucose metabolism (Watt et al., 2006; Glund et al., 2007; Brandt et al., 2015). Among these members, our study showed evidence that LIF is upregulated at mRNA as well as protein level following EPS. Thus, in the current study, we examined the effect of acute LIF treatment on glucose uptake in skeletal muscle cells established from middle-aged women and men. Our result showed that exposure of myotubes to LIF enhanced glucose uptake suggesting that LIF might possibly have a role in autocrine regulation of glucose metabolism in skeletal muscle.

However, the concentration of LIF used in the present study is substantially higher than what was found in media after EPS and the experimental conditions cannot be directly compared. Nonetheless, our finding was in line with previously reported data from mice (Brandt et al., 2015), where acute incubation of isolated soleus and EDL muscles with LIF for 30 min resulted in an increased uptake of glucose. In the same study, the authors proposed that the PI3-kinase/mTORC2/Akt signaling pathway may at least to some degree play a permissive role in LIF-stimulated glucose uptake. On the other hand, activation of AMPK was not found to be a contributing factor in LIF-stimulated glucose uptake in muscle (Brandt et al., 2015). Further studies are needed to elucidate the role of LIF with regard to its role in glucose metabolism.

Finally, comparison of regulated proteins and genes across the proteomics and RNA seq datasets obtained from myotubes of the same young male donors submitted to EPS protocol 1 showed only a handful of significantly regulated proteins/genes to be commonly shared between the secreted proteins, the cell proteome or the cell transcriptome, and none in all of them. However, this might be a result of delayed protein synthesis and protein transport. It is known that induced transcription does not immediately lead to increased protein levels since maturation, export, and translation of mRNA take some time (Liu et al., 2016). Thus, a direct comparison between protein levels and their coding transcripts originated from the same cells, and exposed to the same condition after such short timescale may not be appropriate.

In summary, we have demonstrated improved substrate metabolism in human skeletal muscle cells jointly with comprehensively changed profile of genes and proteins following electrical pulse stimulation. By using a combination of proteomic and high-throughput RNA sequencing, we identified a number of proteins and genes that were differentially regulated in myotubes from young male subjects exposed to EPS protocol 1. Microarray analysis was employed for another EPS protocol with higher voltage and longer duration of stimulation on myotubes from middle-aged women to compare regulated transcripts with the previous donor group. Here, we found some degree of overlap between the different age groups as well as *in vitro* exercise interventions regardless of donor differences and EPS conditions. GO enrichment analysis of differentially expressed proteins and DEGs provided insight into biological processes associated with energy metabolism. Results from this study indicate that the improved metabolic outcomes observed in EPS-treated myotubes could have been mediated through, but not limited to, the induction of metabolic pathways, such as glycolysis and oxidative metabolism, as well as cellular response to oxidative stress, autophagy/mitophagy and AMPK activation. Moreover, we showed that regulation of muscle metabolism could also be attributed by myokines exerting their effect in an autocrine fashion. While more research is needed, our data would provide a valuable resource for understanding the complex biology of exercise in mediating metabolic adaptation in skeletal muscle.

## Data Availability

The datasets presented in this study can be found in online repositories. The names of the repository/repositories and accession number(s) can be found in the article/Supplementary Material.

## Abbreviations

EPS, Electrical pulse stimulation; DEGs, Differentially expressed genes; GO, Gene ontology; LIF, leukemia inhibitory factor; AMPK, AMP-activated protein kinase; PGC-1α, peroxisome proliferator-activated receptor gamma coactivator 1-alpha.
