# Supplementary material for: Insight Into the Metabolic Adaptations of Electrically Pulse-Stimulated Human Myotubes Using Global Analysis of the Transcriptome and Proteome
Source: Front Physiol. 2022 Jul 6;13:928195. doi: 10.3389/fphys.2022.928195 (PMC9298736; doi:10.3389/fphys.2022.928195)
Supplement: Supplementary file 1 [file Table1.DOCX]

**Supplementary table 1: Differentially expressed genes following 24 h of EPS (2ms, 10V, and 1Hz)**

| **gene_id** | **gene_name** | **gene_description** | **padj** | **log2FoldChange** |
| --- | --- | --- | --- | --- |
| ENSG00000205358 | MT1H | metallothionein 1H | 3,05E-07 | 9,73 |
| ENSG00000125144 | MT1G | metallothionein 1G | 3,87E-42 | 9,52 |
| ENSG00000029559 | IBSP | integrin binding sialoprotein | 1,55E-02 | 6,41 |
| ENSG00000108342 | CSF3 | colony stimulating factor 3 | 2,18E-23 | 6,40 |
| ENSG00000162897 | FCAMR | Fc fragment of IgA and IgM receptor | 8,07E-02 | 6,22 |
| ENSG00000277632 | CCL3 | C-C motif chemokine ligand 3 | 8,08E-05 | 6,21 |
| ENSG00000235643 | LINC01647 | long intergenic non-protein coding RNA 1647 | 2,14E-02 | 5,49 |
| ENSG00000101336 | HCK | HCK proto-oncogene, Src family tyrosine kinase | 1,66E-02 | 5,43 |
| ENSG00000198417 | MT1F | metallothionein 1F | 2,30E-28 | 5,35 |
| ENSG00000163435 | ELF3 | E74 like ETS transcription factor 3 | 2,50E-06 | 5,28 |
| ENSG00000230392 | AC004835.1 | uncharacterized LOC101928336 | 1,89E-01 | 5,13 |
| ENSG00000169688 | MT1B | metallothionein 1B | 3,06E-01 | 5,06 |
| ENSG00000276085 | CCL3L1 | C-C motif chemokine ligand 3 like 1 | 1,16E-01 | 5,03 |
| ENSG00000171115 | GIMAP8 | GTPase, IMAP family member 8 | 5,49E-02 | 4,98 |
| ENSG00000007908 | SELE | selectin E | 2,70E-01 | 4,59 |
| ENSG00000173110 | HSPA6 | heat shock protein family A (Hsp70) member 6 | 2,23E-04 | 4,58 |
| ENSG00000183908 | LRRC55 | leucine rich repeat containing 55 | 1,00E+00 | 4,47 |
| ENSG00000166920 | C15orf48 | chromosome 15 open reading frame 48 | 1,41E-04 | 4,42 |
| ENSG00000060558 | GNA15 | G protein subunit alpha 15 | 4,35E-02 | 4,38 |
| ENSG00000205364 | MT1M | metallothionein 1M | 3,21E-20 | 4,32 |
| ENSG00000237988 | OR2I1P | olfactory receptor family 2 subfamily I member 1 pseudogene | 1,41E-01 | 4,32 |
| ENSG00000012504 | NR1H4 | nuclear receptor subfamily 1 group H member 4 | 3,26E-01 | 4,28 |
| ENSG00000135373 | EHF | ETS homologous factor | 1,60E-03 | 4,24 |
| ENSG00000223518 | CSNK1A1P1 | casein kinase 1 alpha 1 pseudogene 1 | 1,00E+00 | 4,08 |
| ENSG00000136244 | IL6 | interleukin 6 | 1,50E-09 | 4,08 |
| ENSG00000137766 | UNC13C | unc-13 homolog C | 1,00E+00 | 4,06 |
| ENSG00000170374 | SP7 | Sp7 transcription factor | 1,65E-01 | 3,96 |
| ENSG00000161270 | NPHS1 | NPHS1, nephrin | 1,00E+00 | 3,86 |
| ENSG00000093134 | VNN3 | vanin 3 | 9,47E-02 | 3,85 |
| ENSG00000102010 | BMX | BMX non-receptor tyrosine kinase | 1,00E+00 | 3,79 |
| ENSG00000151790 | TDO2 | tryptophan 2,3-dioxygenase | 1,01E-01 | 3,74 |
| ENSG00000205362 | MT1A | metallothionein 1A | 6,94E-05 | 3,72 |
| ENSG00000137745 | MMP13 | matrix metallopeptidase 13 | 2,79E-01 | 3,72 |
| ENSG00000169429 | CXCL8 | C-X-C motif chemokine ligand 8 | 2,06E-05 | 3,70 |
| ENSG00000187193 | MT1X | metallothionein 1X | 1,54E-31 | 3,69 |
| ENSG00000157131 | C8A | complement C8 alpha chain | 1,00E+00 | 3,69 |
| **gene_id** | **gene_name** | **gene_description** | **padj** | **log2FoldChange** |
| ENSG00000175746 | C15orf54 | chromosome 15 open reading frame 54 (putative) | 1,00E+00 | 3,58 |
| ENSG00000196664 | TLR7 | toll like receptor 7 | 1,00E+00 | 3,56 |
| ENSG00000139656 | SMIM2 | small integral membrane protein 2 | 1,00E+00 | 3,48 |
| ENSG00000173626 | TRAPPC3L | trafficking protein particle complex 3 like | 1,00E+00 | 3,48 |
| ENSG00000163739 | CXCL1 | C-X-C motif chemokine ligand 1 | 1,19E-02 | 3,47 |
| ENSG00000225217 | HSPA7 | heat shock protein family A (Hsp70) member 7 | 2,79E-01 | 3,46 |
| ENSG00000124102 | PI3 | peptidase inhibitor 3 | 6,94E-05 | 3,44 |
| ENSG00000229644 | NAMPTP1 | nicotinamide phosphoribosyltransferase pseudogene 1 | 3,72E-03 | 3,39 |
| ENSG00000115009 | CCL20 | C-C motif chemokine ligand 20 | 3,83E-03 | 3,38 |
| ENSG00000269466 | H3.Y | histone cluster 2, H3c pseudogene | 1,00E+00 | 3,32 |
| ENSG00000255921 | AC026310.2 | novel transcript, antisense to BCAT1 | 9,94E-02 | 3,32 |
| ENSG00000260953 | AC009093.4 | novel transcript | 1,00E+00 | 3,30 |
| ENSG00000237133 | AC020594.1 | novel transcript | 1,00E+00 | 3,27 |
| ENSG00000170075 | GPR37L1 | G protein-coupled receptor 37 like 1 | 5,43E-03 | 3,27 |
| ENSG00000165805 | C12orf50 | chromosome 12 open reading frame 50 | 1,00E+00 | 3,26 |
| ENSG00000136695 | IL36RN | interleukin 36 receptor antagonist | 2,12E-01 | 3,22 |
| ENSG00000128271 | ADORA2A | adenosine A2a receptor | 1,04E-01 | 3,16 |
| ENSG00000253522 | MIR3142HG | MIR3142 host gene | 3,77E-01 | 3,16 |
| ENSG00000253954 | HMGN1P38 | high mobility group nucleosome binding domain 1 pseudogene 38 | 1,00E+00 | 3,14 |
| ENSG00000261210 | CLEC19A | C-type lectin domain containing 19A | 1,00E+00 | 3,14 |
| ENSG00000163734 | CXCL3 | C-X-C motif chemokine ligand 3 | 3,44E-02 | 3,13 |
| ENSG00000267909 | CCDC177 | coiled-coil domain containing 177 | 1,00E+00 | 3,11 |
| ENSG00000115008 | IL1A | interleukin 1 alpha | 5,49E-02 | 3,09 |
| ENSG00000139572 | GPR84 | G protein-coupled receptor 84 | 1,00E+00 | 3,08 |
| ENSG00000242258 | LINC00996 | long intergenic non-protein coding RNA 996 | 1,00E+00 | 3,07 |
| ENSG00000184731 | FAM110C | family with sequence similarity 110 member C | 1,00E+00 | 3,06 |
| ENSG00000180061 | TMEM150B | transmembrane protein 150B | 1,00E+00 | 3,05 |
| ENSG00000179799 | OR7E22P | olfactory receptor family 7 subfamily E member 22 pseudogene | 8,54E-01 | 3,04 |
| ENSG00000136689 | IL1RN | interleukin 1 receptor antagonist | 7,57E-01 | 3,02 |
| ENSG00000197406 | DIO3 | iodothyronine deiodinase 3 | 1,00E+00 | 3,02 |
| ENSG00000285210 | AL136382.1 | novel transcript | 1,00E+00 | 3,00 |
| ENSG00000108700 | CCL8 | C-C motif chemokine ligand 8 | 8,88E-01 | 2,99 |
| ENSG00000277270 | AL160412.1 | novel transcript | 1,00E+00 | 2,98 |
| ENSG00000125538 | IL1B | interleukin 1 beta | 4,63E-03 | 2,97 |
| ENSG00000271503 | CCL5 | C-C motif chemokine ligand 5 | 2,50E-02 | 2,97 |
| ENSG00000145451 | GLRA3 | glycine receptor alpha 3 | 1,00E+00 | 2,92 |
| ENSG00000229261 | AL596223.1 | uncharacterized LOC101928994 | 5,78E-01 | 2,91 |
| ENSG00000081041 | CXCL2 | C-X-C motif chemokine ligand 2 | 1,09E-02 | 2,90 |
| **gene_id** | **gene_name** | **gene_description** | **padj** | **log2FoldChange** |
| ENSG00000224680 | PLA2G12AP1 | phospholipase A2 group XIIA pseudogene 1 | 1,00E+00 | 2,90 |
| ENSG00000183734 | ASCL2 | achaete-scute family bHLH transcription factor 2 | 1,00E+00 | 2,90 |
| ENSG00000140284 | SLC27A2 | solute carrier family 27 member 2 | 1,00E+00 | 2,89 |
| ENSG00000163735 | CXCL5 | C-X-C motif chemokine ligand 5 | 9,94E-02 | 2,89 |
| ENSG00000241104 | CEACAMP10 | carcinoembryonic antigen related cell adhesion molecule pseudogene 10 | 4,48E-01 | 2,89 |
| ENSG00000205300 | AL356414.1 | novel transcript | 2,27E-01 | 2,88 |
| ENSG00000173239 | LIPM | lipase family member M | 5,81E-01 | 2,87 |
| ENSG00000139767 | SRRM4 | serine/arginine repetitive matrix 4 | 1,80E-01 | 2,85 |
| ENSG00000101443 | WFDC2 | WAP four-disulfide core domain 2 | 1,00E+00 | 2,84 |
| ENSG00000236882 | LINC01554 | long intergenic non-protein coding RNA 1554 | 1,52E-01 | 2,83 |
| ENSG00000226644 | AL121899.1 | uncharacterized LOC388780 | 1,00E+00 | 2,81 |
| ENSG00000104321 | TRPA1 | transient receptor potential cation channel subfamily A member 1 | 2,56E-02 | 2,79 |
| ENSG00000124875 | CXCL6 | C-X-C motif chemokine ligand 6 | 5,98E-01 | 2,79 |
| ENSG00000215267 | AKR1C7P | aldo-keto reductase family 1 member C7, pseudogene | 1,89E-01 | 2,78 |
| ENSG00000125968 | ID1 | inhibitor of DNA binding 1, HLH protein | 1,57E-01 | 2,78 |
| ENSG00000197632 | SERPINB2 | serpin family B member 2 | 9,71E-03 | 2,77 |
| ENSG00000004468 | CD38 | CD38 molecule | 7,28E-01 | 2,76 |
| ENSG00000157856 | DRC1 | dynein regulatory complex subunit 1 | 1,00E+00 | 2,76 |
| ENSG00000231274 | SBK3 | SH3 domain binding kinase family member 3 | 1,80E-01 | 2,72 |
| ENSG00000274956 | NKAIN3-IT1 | NKAIN3 intronic transcript | 1,00E+00 | 2,72 |
| ENSG00000228917 | AL591806.1 | uncharacterized LOC105371471 | 1,00E+00 | 2,70 |
| ENSG00000272573 | MUSTN1 | musculoskeletal, embryonic nuclear protein 1 | 1,00E+00 | 2,70 |
| ENSG00000176754 | LINC00303 | long intergenic non-protein coding RNA 303 | 1,00E+00 | 2,68 |
| ENSG00000214510 | SPINK13 | serine peptidase inhibitor, Kazal type 13 (putative) | 1,00E+00 | 2,68 |
| ENSG00000231473 | RB1-DT | RB1 divergent transcript | 1,00E+00 | 2,68 |
| ENSG00000260979 | AC022167.3 | novel transcript | 1,00E+00 | 2,67 |
| ENSG00000171903 | CYP4F11 | cytochrome P450 family 4 subfamily F member 11 | 4,00E-01 | 2,67 |
| ENSG00000073734 | ABCB11 | ATP binding cassette subfamily B member 11 | 1,00E+00 | 2,66 |
| ENSG00000262333 | HNRNPA1P16 | heterogeneous nuclear ribonucleoprotein A1 pseudogene 16 | 1,00E+00 | 2,65 |
| ENSG00000169715 | MT1E | metallothionein 1E | 3,02E-14 | 2,65 |
| ENSG00000229498 | AC105053.1 | uncharacterized LOC284950 | 1,00E+00 | 2,64 |
| ENSG00000225169 | BRI3P1 | brain protein I3 pseudogene 1 | 1,00E+00 | 2,64 |
| ENSG00000255553 | AP003733.3 | uncharacterized LOC100507521 | 1,21E-01 | 2,63 |
| ENSG00000231638 | LUARIS | lncRNA upregulator of antiviral response interferon signaling | 1,00E+00 | 2,62 |
| ENSG00000224490 | TTC21B-AS1 | TTC21B antisense RNA 1 | 1,00E+00 | 2,62 |
| ENSG00000163874 | ZC3H12A | zinc finger CCCH-type containing 12A | 9,69E-06 | 2,61 |
| ENSG00000249755 | AC083829.1 | CD53 molecule (CD53) pseudogene | 3,45E-01 | 2,61 |
| ENSG00000229781 | AC013444.1 | RRN3 RNA polymerase I transcription factor homolog (S. cerevisiae) (RRN3) pseudogene | 1,00E+00 | 2,59 |
| **gene_id** | **gene_name** | **gene_description** | **padj** | **log2FoldChange** |
| ENSG00000123610 | TNFAIP6 | TNF alpha induced protein 6 | 3,91E-02 | 2,59 |
| ENSG00000107165 | TYRP1 | tyrosinase related protein 1 | 1,00E+00 | 2,59 |
| ENSG00000205361 | MT1DP | metallothionein 1D, pseudogene | 4,74E-01 | 2,55 |
| ENSG00000231574 | LINC02015 | long intergenic non-protein coding RNA 2015 | 1,42E-01 | 2,54 |
| ENSG00000146666 | LINC00525 | long intergenic non-protein coding RNA 525 | 1,00E+00 | 2,53 |
| ENSG00000073756 | PTGS2 | prostaglandin-endoperoxide synthase 2 | 1,65E-01 | 2,52 |
| ENSG00000131203 | IDO1 | indoleamine 2,3-dioxygenase 1 | 3,77E-01 | 2,49 |
| ENSG00000163106 | HPGDS | hematopoietic prostaglandin D synthase | 1,00E+00 | 2,49 |
| ENSG00000108688 | CCL7 | C-C motif chemokine ligand 7 | 1,45E-01 | 2,48 |
| ENSG00000187173 | LCE2A | late cornified envelope 2A | 9,88E-01 | 2,44 |
| ENSG00000280587 | LINC01348 | long intergenic non-protein coding RNA 1348 | 5,72E-01 | 2,44 |
| ENSG00000254919 | AC090692.1 | novel transcript | 1,00E+00 | 2,43 |
| ENSG00000178803 | ADORA2A-AS1 | ADORA2A antisense RNA 1 | 1,98E-01 | 2,43 |
| ENSG00000164400 | CSF2 | colony stimulating factor 2 | 9,50E-01 | 2,42 |
| ENSG00000230611 | HMGB1P27 | high mobility group box 1 pseudogene 27 | 2,98E-01 | 2,35 |
| ENSG00000237094 | AL732372.2 | pseudogene similar to part of septin 14 SEPT14 | 1,00E+00 | 2,33 |
| ENSG00000234695 | AC002076.1 | uncharacterized LOC105375401 | 1,00E+00 | 2,32 |
| ENSG00000135298 | ADGRB3 | adhesion G protein-coupled receptor B3 | 7,70E-01 | 2,32 |
| ENSG00000105835 | NAMPT | nicotinamide phosphoribosyltransferase | 3,44E-02 | 2,31 |
| ENSG00000163016 | ALMS1P1 | ALMS1, centrosome and basal body associated protein pseudogene 1 | 1,00E+00 | 2,31 |
| ENSG00000234750 | AC012618.2 | ribosomal protein S2 (RPS2) pseudogene | 1,00E+00 | 2,30 |
| ENSG00000003989 | SLC7A2 | solute carrier family 7 member 2 | 1,80E-01 | 2,30 |
| ENSG00000232934 | AL157786.1 | novel transcript, antisense to ACSL5 | 1,00E+00 | 2,30 |
| ENSG00000215022 | AL008729.1 | uncharacterized LOC100130357 | 3,30E-01 | 2,24 |
| ENSG00000205502 | C2CD4B | C2 calcium dependent domain containing 4B | 5,80E-01 | 2,23 |
| ENSG00000261618 | AC083837.1 | novel transcript | 1,00E+00 | 2,21 |
| ENSG00000227744 | LINC01940 | long intergenic non-protein coding RNA 1940 | 5,00E-01 | 2,20 |
| ENSG00000166670 | MMP10 | matrix metallopeptidase 10 | 3,65E-02 | 2,19 |
| ENSG00000042980 | ADAM28 | ADAM metallopeptidase domain 28 | 2,77E-01 | 2,19 |
| ENSG00000120337 | TNFSF18 | TNF superfamily member 18 | 1,22E-01 | 2,18 |
| ENSG00000236049 | LINC01920 | long intergenic non-protein coding RNA 1920 | 8,22E-01 | 2,14 |
| ENSG00000230790 | AC012456.2 | novel transcript | 1,00E+00 | 2,13 |
| ENSG00000236938 | AC003092.2 | novel transcript | 5,06E-01 | 2,12 |
| ENSG00000234789 | AL590369.1 | novel transcript | 1,00E+00 | 2,12 |
| ENSG00000268423 | AC093503.1 | novel transcript, sense intronic to PPP5D1 | 1,00E+00 | 2,12 |
| ENSG00000092345 | DAZL | deleted in azoospermia like | 8,54E-01 | 2,11 |
| ENSG00000158473 | CD1D | CD1d molecule | 7,32E-01 | 2,10 |
| ENSG00000158748 | HTR6 | 5-hydroxytryptamine receptor 6 | 9,84E-01 | 2,09 |
| **gene_id** | **gene_name** | **gene_description** | **padj** | **log2FoldChange** |
| ENSG00000279908 | AC026748.1 | tec | 1,00E+00 | 2,08 |
| ENSG00000070190 | DAPP1 | dual adaptor of phosphotyrosine and 3-phosphoinositides 1 | 6,30E-01 | 2,08 |
| ENSG00000235885 | LINC01828 | long intergenic non-protein coding RNA 1828 | 5,10E-01 | 2,08 |
| ENSG00000260549 | MT1L | metallothionein 1L, pseudogene | 1,99E-05 | 2,07 |
| ENSG00000236136 | ADORA2BP1 | adenosine A2b receptor pseudogene 1 | 2,20E-01 | 2,06 |
| ENSG00000144802 | NFKBIZ | NFKB inhibitor zeta | 6,60E-03 | 2,06 |
| ENSG00000198734 | F5 | coagulation factor V | 9,98E-01 | 2,05 |
| ENSG00000159167 | STC1 | stanniocalcin 1 | 5,62E-02 | 2,05 |
| ENSG00000168229 | PTGDR | prostaglandin D2 receptor | 3,16E-01 | 2,04 |
| ENSG00000224321 | RPL12P14 | ribosomal protein L12 pseudogene 14 | 7,92E-01 | 2,03 |
| ENSG00000162894 | FCMR | Fc fragment of IgM receptor | 4,18E-03 | 2,02 |
| ENSG00000162892 | IL24 | interleukin 24 | 3,18E-01 | 2,02 |
| ENSG00000203883 | SOX18 | SRY-box 18 | 4,64E-01 | 2,01 |
| ENSG00000028277 | POU2F2 | POU class 2 homeobox 2 | 1,04E-01 | 2,00 |
| ENSG00000104951 | IL4I1 | interleukin 4 induced 1 | 6,43E-01 | 2,00 |
| ENSG00000069535 | MAOB | monoamine oxidase B | 2,92E-01 | 1,98 |
| ENSG00000146216 | TTBK1 | tau tubulin kinase 1 | 1,00E+00 | 1,97 |
| ENSG00000240602 | AADACP1 | arylacetamide deacetylase pseudogene 1 | 4,75E-01 | 1,97 |
| ENSG00000223764 | LINC02593 | long intergenic non-protein coding RNA 2593 | 3,20E-01 | 1,94 |
| ENSG00000187634 | SAMD11 | sterile alpha motif domain containing 11 | 3,28E-01 | 1,94 |
| ENSG00000023445 | BIRC3 | baculoviral IAP repeat containing 3 | 1,73E-02 | 1,94 |
| ENSG00000233817 | AL162727.1 | novel transcript | 7,76E-01 | 1,94 |
| ENSG00000105509 | HAS1 | hyaluronan synthase 1 | 3,30E-01 | 1,92 |
| ENSG00000261220 | AC103706.1 | novel transcript | 5,80E-01 | 1,91 |
| ENSG00000197921 | HES5 | hes family bHLH transcription factor 5 | 8,54E-01 | 1,91 |
| ENSG00000137491 | SLCO2B1 | solute carrier organic anion transporter family member 2B1 | 3,44E-02 | 1,90 |
| ENSG00000148344 | PTGES | prostaglandin E synthase | 2,06E-01 | 1,90 |
| ENSG00000118193 | KIF14 | kinesin family member 14 | 2,76E-01 | 1,88 |
| ENSG00000227158 | AC073621.1 | ligand of numb-protein X 1, E3 ubiquitin protein ligase pseudogene | 1,04E-01 | 1,88 |
| ENSG00000236824 | BCYRN1 | brain cytoplasmic RNA 1 | 7,33E-01 | 1,87 |
| ENSG00000235812 | ADAM21P1 | ADAM metallopeptidase domain 21 pseudogene 1 | 1,00E+00 | 1,86 |
| ENSG00000138135 | CH25H | cholesterol 25-hydroxylase | 8,25E-01 | 1,86 |
| ENSG00000284906 | AC091057.6 | Rho GTPase-activating protein 11B | 1,00E+00 | 1,85 |
| ENSG00000274756 | AC243732.1 | ribosomal protein S2 (RPS2) pseudogene | 1,00E+00 | 1,83 |
| ENSG00000117318 | ID3 | inhibitor of DNA binding 3, HLH protein | 2,14E-01 | 1,83 |
| ENSG00000285744 | AC083837.2 | novel transcript | 5,01E-01 | 1,81 |
| ENSG00000160111 | CPAMD8 | C3 and PZP like, alpha-2-macroglobulin domain containing 8 | 1,16E-01 | 1,80 |
| ENSG00000049249 | TNFRSF9 | TNF receptor superfamily member 9 | 9,21E-01 | 1,79 |
| **gene_id** | **gene_name** | **gene_description** | **padj** | **log2FoldChange** |
| ENSG00000198535 | C2CD4A | C2 calcium dependent domain containing 4A | 2,10E-01 | 1,78 |
| ENSG00000105929 | ATP6V0A4 | ATPase H+ transporting V0 subunit a4 | 1,00E+00 | 1,78 |
| ENSG00000050730 | TNIP3 | TNFAIP3 interacting protein 3 | 3,72E-03 | 1,77 |
| ENSG00000213885 | RPL13AP7 | ribosomal protein L13a pseudogene 7 | 1,00E+00 | 1,77 |
| ENSG00000163347 | CLDN1 | claudin 1 | 1,36E-01 | 1,76 |
| ENSG00000100739 | BDKRB1 | bradykinin receptor B1 | 4,64E-01 | 1,75 |
| ENSG00000228412 | AL022068.1 | uncharacterized LOC100506885 | 2,12E-01 | 1,75 |
| ENSG00000254783 | AP003084.1 | immunoglobin superfamily, member 21 (IGSF21) pseudogene | 8,54E-01 | 1,75 |
| ENSG00000131737 | KRT34 | keratin 34 | 8,38E-01 | 1,74 |
| ENSG00000278075 | AC114341.1 | novel transcript | 8,32E-01 | 1,74 |
| ENSG00000196611 | MMP1 | matrix metallopeptidase 1 | 2,97E-01 | 1,73 |
| ENSG00000206073 | SERPINB4 | serpin family B member 4 | 9,34E-01 | 1,72 |
| ENSG00000120949 | TNFRSF8 | TNF receptor superfamily member 8 | 4,94E-01 | 1,72 |
| ENSG00000108551 | RASD1 | ras related dexamethasone induced 1 | 9,01E-02 | 1,72 |
| ENSG00000125148 | MT2A | metallothionein 2A | 1,99E-05 | 1,71 |
| ENSG00000131979 | GCH1 | GTP cyclohydrolase 1 | 3,77E-01 | 1,70 |
| ENSG00000164181 | ELOVL7 | ELOVL fatty acid elongase 7 | 1,56E-01 | 1,69 |
| ENSG00000189120 | SP6 | Sp6 transcription factor | 3,16E-01 | 1,69 |
| ENSG00000115919 | KYNU | kynureninase | 1,95E-01 | 1,68 |
| ENSG00000197249 | SERPINA1 | serpin family A member 1 | 6,43E-01 | 1,68 |
| ENSG00000153823 | PID1 | phosphotyrosine interaction domain containing 1 | 1,06E-01 | 1,68 |
| ENSG00000259436 | AC010247.2 | novel transcript, antisense to POU2F2 | 9,50E-01 | 1,67 |
| ENSG00000237605 | AL591846.2 | novel transcript | 9,45E-01 | 1,66 |
| ENSG00000128849 | CGNL1 | cingulin like 1 | 1,27E-02 | 1,65 |
| ENSG00000128342 | LIF | LIF, interleukin 6 family cytokine | 3,55E-03 | 1,61 |
| ENSG00000168874 | ATOH8 | atonal bHLH transcription factor 8 | 1,65E-01 | 1,61 |
| ENSG00000123689 | G0S2 | G0/G1 switch 2 | 8,88E-01 | 1,61 |
| ENSG00000159753 | CARMIL2 | capping protein regulator and myosin 1 linker 2 | 4,94E-01 | 1,60 |
| ENSG00000138821 | SLC39A8 | solute carrier family 39 member 8 | 6,57E-01 | 1,59 |
| ENSG00000231993 | EP300-AS1 | EP300 antisense RNA 1 | 1,00E+00 | 1,59 |
| ENSG00000134321 | RSAD2 | radical S-adenosyl methionine domain containing 2 | 1,07E-01 | 1,58 |
| ENSG00000163121 | NEURL3 | neuralized E3 ubiquitin protein ligase 3 | 1,00E+00 | 1,57 |
| ENSG00000118503 | TNFAIP3 | TNF alpha induced protein 3 | 7,84E-02 | 1,56 |
| ENSG00000255046 | AC069185.1 | novel transcript | 7,60E-01 | 1,56 |
| ENSG00000187068 | C3orf70 | chromosome 3 open reading frame 70 | 4,12E-01 | 1,55 |
| ENSG00000113494 | PRLR | prolactin receptor | 8,17E-02 | 1,54 |
| ENSG00000273091 | AP000255.1 | novel transcript | 1,00E+00 | 1,54 |
| ENSG00000185101 | ANO9 | anoctamin 9 | 8,36E-01 | 1,54 |
| **gene_id** | **gene_name** | **gene_description** | **padj** | **log2FoldChange** |
| ENSG00000156510 | HKDC1 | hexokinase domain containing 1 | 9,11E-01 | 1,53 |
| ENSG00000249978 | TRGV7 | T cell receptor gamma variable 7 (pseudogene) | 1,00E+00 | 1,53 |
| ENSG00000143995 | MEIS1 | Meis homeobox 1 | 5,46E-01 | 1,53 |
| ENSG00000241218 | AC063944.2 | citrate synthase (CS) pseudogene | 1,00E+00 | 1,52 |
| ENSG00000188056 | TREML4 | triggering receptor expressed on myeloid cells like 4 | 1,00E+00 | 1,51 |
| ENSG00000272196 | HIST2H2AA4 | histone cluster 2 H2A family member a4 | 8,03E-02 | 1,50 |
| ENSG00000275552 | AC243965.2 | novel transcript, antisense ABHD4 | 6,52E-01 | 1,50 |
| ENSG00000234883 | MIR155HG | MIR155 host gene | 9,34E-01 | 1,50 |
| ENSG00000137033 | IL33 | interleukin 33 | 7,53E-01 | 1,49 |
| ENSG00000105289 | TJP3 | tight junction protein 3 | 8,69E-01 | 1,48 |
| ENSG00000104722 | NEFM | neurofilament medium | 1,00E+00 | 1,48 |
| ENSG00000182329 | KIAA2012 | KIAA2012 | 1,00E+00 | 1,47 |
| ENSG00000100906 | NFKBIA | NFKB inhibitor alpha | 3,37E-02 | 1,46 |
| ENSG00000115738 | ID2 | inhibitor of DNA binding 2 | 3,74E-01 | 1,46 |
| ENSG00000249035 | CLMAT3 | colorectal liver metastasis associated transcript 3 | 1,00E+00 | 1,46 |
| ENSG00000112742 | TTK | TTK protein kinase | 2,70E-01 | 1,45 |
| ENSG00000155966 | AFF2 | AF4/FMR2 family member 2 | 6,46E-02 | 1,44 |
| ENSG00000163131 | CTSS | cathepsin S | 9,83E-01 | 1,44 |
| ENSG00000176907 | TCIM | transcriptional and immune response regulator | 1,80E-01 | 1,44 |
| ENSG00000105825 | TFPI2 | tissue factor pathway inhibitor 2 | 5,98E-01 | 1,41 |
| ENSG00000164283 | ESM1 | endothelial cell specific molecule 1 | 9,39E-01 | 1,40 |
| ENSG00000006606 | CCL26 | C-C motif chemokine ligand 26 | 5,34E-04 | 1,40 |
| ENSG00000123870 | ZNF137P | zinc finger protein 137, pseudogene | 4,58E-01 | 1,40 |
| ENSG00000125657 | TNFSF9 | TNF superfamily member 9 | 5,62E-01 | 1,39 |
| ENSG00000134326 | CMPK2 | cytidine/uridine monophosphate kinase 2 | 6,67E-01 | 1,39 |
| ENSG00000248371 | LINC02056 | long intergenic non-protein coding RNA 2056 | 6,53E-01 | 1,39 |
| ENSG00000224023 | FLJ37035 | uncharacterized LOC399821 | 1,00E+00 | 1,37 |
| ENSG00000124479 | NDP | NDP, norrin cystine knot growth factor | 6,66E-01 | 1,37 |
| ENSG00000147234 | FRMPD3 | FERM and PDZ domain containing 3 | 1,00E+00 | 1,36 |
| ENSG00000234678 | ELF3-AS1 | ELF3 antisense RNA 1 | 9,98E-01 | 1,36 |
| ENSG00000100292 | HMOX1 | heme oxygenase 1 | 1,14E-03 | 1,36 |
| ENSG00000112299 | VNN1 | vanin 1 | 3,61E-01 | 1,36 |
| ENSG00000152270 | PDE3B | phosphodiesterase 3B | 1,00E+00 | 1,35 |
| ENSG00000108691 | CCL2 | C-C motif chemokine ligand 2 | 7,96E-01 | 1,35 |
| ENSG00000114270 | COL7A1 | collagen type VII alpha 1 chain | 1,00E+00 | 1,35 |
| ENSG00000177757 | FAM87B | family with sequence similarity 87 member B | 4,19E-01 | 1,34 |
| ENSG00000285802 | AL450043.1 | novel transcript | 7,52E-01 | 1,34 |
| ENSG00000069696 | DRD4 | dopamine receptor D4 | 5,33E-01 | 1,33 |
| **gene_id** | **gene_name** | **gene_description** | **padj** | **log2FoldChange** |
| ENSG00000169607 | CKAP2L | cytoskeleton associated protein 2 like | 5,01E-01 | 1,33 |
| ENSG00000188710 | QRFP | pyroglutamylated RFamide peptide | 3,19E-01 | 1,33 |
| ENSG00000137331 | IER3 | immediate early response 3 | 1,78E-01 | 1,31 |
| ENSG00000203711 | C6orf99 | chromosome 6 open reading frame 99 | 1,00E+00 | 1,31 |
| ENSG00000112096 | SOD2 | superoxide dismutase 2 | 5,42E-01 | 1,30 |
| ENSG00000172201 | ID4 | inhibitor of DNA binding 4, HLH protein | 1,00E+00 | 1,30 |
| ENSG00000279170 | AL137784.3 | TEC | 1,00E+00 | 1,30 |
| ENSG00000103522 | IL21R | interleukin 21 receptor | 7,91E-01 | 1,30 |
| ENSG00000125637 | PSD4 | pleckstrin and Sec7 domain containing 4 | 6,71E-01 | 1,30 |
| ENSG00000268812 | AC004264.1 | uncharacterized LOC91370 | 4,85E-01 | 1,29 |
| ENSG00000090376 | IRAK3 | interleukin 1 receptor associated kinase 3 | 8,00E-01 | 1,29 |
| ENSG00000224800 | RPS27AP6 | ribosomal protein S27a pseudogene 6 | 9,84E-01 | 1,28 |
| ENSG00000153162 | BMP6 | bone morphogenetic protein 6 | 5,10E-01 | 1,28 |
| ENSG00000251000 | AC008592.3 | gamma-glutamyl cyclotransferase (GGCT) pseudogene | 1,00E+00 | 1,27 |
| ENSG00000140519 | RHCG | Rh family C glycoprotein | 3,57E-01 | 1,25 |
| ENSG00000133392 | MYH11 | myosin heavy chain 11 | 9,50E-01 | 1,24 |
| ENSG00000165490 | DDIAS | DNA damage induced apoptosis suppressor | 4,58E-01 | 1,23 |
| ENSG00000065320 | NTN1 | netrin 1 | 5,62E-02 | 1,23 |
| ENSG00000134070 | IRAK2 | interleukin 1 receptor associated kinase 2 | 9,94E-02 | 1,22 |
| ENSG00000033122 | LRRC7 | leucine rich repeat containing 7 | 3,00E-01 | 1,22 |
| ENSG00000104635 | SLC39A14 | solute carrier family 39 member 14 | 3,36E-01 | 1,22 |
| ENSG00000118257 | NRP2 | neuropilin 2 | 9,39E-01 | 1,22 |
| ENSG00000159212 | CLIC6 | chloride intracellular channel 6 | 7,00E-01 | 1,22 |
| ENSG00000146457 | WTAP | WT1 associated protein | 4,58E-01 | 1,21 |
| ENSG00000113555 | PCDH12 | protocadherin 12 | 2,23E-01 | 1,21 |
| ENSG00000233058 | LINC00884 | long intergenic non-protein coding RNA 884 | 1,74E-01 | 1,21 |
| ENSG00000228787 | NLGN4Y-AS1 | NLGN4Y antisense RNA 1 | 6,50E-01 | 1,21 |
| ENSG00000177483 | RBM44 | RNA binding motif protein 44 | 9,49E-01 | 1,20 |
| ENSG00000204389 | HSPA1A | heat shock protein family A (Hsp70) member 1A | 1,11E-01 | 1,19 |
| ENSG00000204446 | C9orf170 | chromosome 9 open reading frame 170 | 9,50E-01 | 1,19 |
| ENSG00000133805 | AMPD3 | adenosine monophosphate deaminase 3 | 5,16E-02 | 1,19 |
| ENSG00000187479 | C11orf96 | chromosome 11 open reading frame 96 | 1,65E-01 | 1,18 |
| ENSG00000272084 | AL137127.1 | novel transcript | 6,07E-01 | 1,18 |
| ENSG00000248323 | LUCAT1 | lung cancer associated transcript 1 | 1,48E-01 | 1,18 |
| ENSG00000091972 | CD200 | CD200 molecule | 7,60E-01 | 1,17 |
| ENSG00000184106 | TREML3P | triggering receptor expressed on myeloid cells like 3, pseudogene | 4,64E-01 | 1,16 |
| ENSG00000241081 | RPL22P2 | ribosomal protein L22 pseudogene 2 | 1,00E+00 | 1,15 |
| ENSG00000107968 | MAP3K8 | mitogen-activated protein kinase kinase kinase 8 | 5,98E-01 | 1,15 |
| **gene_id** | **gene_name** | **gene_description** | **padj** | **log2FoldChange** |
| ENSG00000261040 | WFDC21P | WAP four-disulfide core domain 21, pseudogene | 3,66E-01 | 1,15 |
| ENSG00000272153 | AL365330.1 | novel transcript, antisense to LRRC47 | 9,29E-01 | 1,14 |
| ENSG00000124731 | TREM1 | triggering receptor expressed on myeloid cells 1 | 9,49E-01 | 1,14 |
| ENSG00000177459 | ERICH5 | glutamate rich 5 | 1,00E+00 | 1,14 |
| ENSG00000236453 | AC003092.1 | novel transcript | 4,26E-01 | 1,14 |
| ENSG00000081051 | AFP | alpha fetoprotein | 4,26E-01 | 1,13 |
| ENSG00000256001 | AC079949.1 | novel transcript | 7,00E-01 | 1,12 |
| ENSG00000103044 | HAS3 | hyaluronan synthase 3 | 1,06E-01 | 1,11 |
| ENSG00000168685 | IL7R | interleukin 7 receptor | 7,96E-01 | 1,11 |
| ENSG00000260475 | AL353719.1 | novel transcript | 5,98E-01 | 1,11 |
| ENSG00000249641 | HOXC13-AS | HOXC13 antisense RNA | 7,15E-01 | 1,11 |
| ENSG00000240747 | KRBOX1 | KRAB box domain containing 1 | 8,54E-01 | 1,11 |
| ENSG00000106327 | TFR2 | transferrin receptor 2 | 8,03E-01 | 1,10 |
| ENSG00000274422 | AC245060.5 | novel transcript | 1,00E+00 | 1,08 |
| ENSG00000144395 | CCDC150 | coiled-coil domain containing 150 | 5,10E-01 | 1,08 |
| ENSG00000153294 | ADGRF4 | adhesion G protein-coupled receptor F4 | 1,00E+00 | 1,08 |
| ENSG00000247095 | MIR210HG | MIR210 host gene | 4,28E-01 | 1,08 |
| ENSG00000101230 | ISM1 | isthmin 1 | 1,00E+00 | 1,08 |
| ENSG00000244055 | AC007566.1 | novel transcript, antisense to ERVW-1 and PEX1 | 1,00E+00 | 1,07 |
| ENSG00000025708 | TYMP | thymidine phosphorylase | 1,00E+00 | 1,06 |
| ENSG00000228192 | AL512353.1 | novel transcript, antisense to ERMAP | 7,06E-01 | 1,06 |
| ENSG00000134802 | SLC43A3 | solute carrier family 43 member 3 | 9,56E-01 | 1,06 |
| ENSG00000184588 | PDE4B | phosphodiesterase 4B | 7,00E-01 | 1,06 |
| ENSG00000163694 | RBM47 | RNA binding motif protein 47 | 5,22E-01 | 1,06 |
| ENSG00000253837 | AC090197.1 | uncharacterized LOC100507156 | 5,81E-01 | 1,05 |
| ENSG00000279491 | AP003733.4 | uncharacterized LOC399900 | 5,67E-01 | 1,05 |
| ENSG00000253227 | AC090192.2 | novel transcript | 5,61E-01 | 1,04 |
| ENSG00000282885 | AL627171.2 | novel transcript | 4,94E-01 | 1,03 |
| ENSG00000149968 | MMP3 | matrix metallopeptidase 3 | 1,00E+00 | 1,03 |
| ENSG00000136160 | EDNRB | endothelin receptor type B | 9,11E-01 | 1,02 |
| ENSG00000138778 | CENPE | centromere protein E | 7,98E-01 | 1,01 |
| ENSG00000110944 | IL23A | interleukin 23 subunit alpha | 9,63E-01 | 1,01 |
| ENSG00000119508 | NR4A3 | nuclear receptor subfamily 4 group A member 3 | 4,12E-01 | 1,00 |
| ENSG00000100902 | PSMA6 | proteasome subunit alpha 6 | 4,64E-03 | 0,99 |
| ENSG00000175592 | FOSL1 | FOS like 1, AP-1 transcription factor subunit | 9,94E-02 | 0,99 |
| ENSG00000203837 | PNLIPRP3 | pancreatic lipase related protein 3 | 9,50E-01 | 0,98 |
| ENSG00000198053 | SIRPA | signal regulatory protein alpha | 5,62E-01 | 0,98 |
| ENSG00000213085 | CFAP45 | cilia and flagella associated protein 45 | 4,70E-01 | 0,98 |
| **gene_id** | **gene_name** | **gene_description** | **padj** | **log2FoldChange** |
| ENSG00000216285 | AC078819.1 | phosphoglycerate mutase 1 (brain) (PGAM1) pseudogene | 5,67E-01 | 0,97 |
| ENSG00000234155 | LINC02535 | long intergenic non-protein coding RNA 2535 | 9,50E-01 | 0,96 |
| ENSG00000141294 | LRRC46 | leucine rich repeat containing 46 | 1,00E+00 | 0,96 |
| ENSG00000119915 | ELOVL3 | ELOVL fatty acid elongase 3 | 2,70E-01 | 0,95 |
| ENSG00000138685 | FGF2 | fibroblast growth factor 2 | 4,64E-01 | 0,94 |
| ENSG00000180044 | C3orf80 | chromosome 3 open reading frame 80 | 8,69E-01 | 0,93 |
| ENSG00000232415 | ELN-AS1 | ELN antisense RNA 1 | 9,85E-01 | 0,93 |
| ENSG00000105711 | SCN1B | sodium voltage-gated channel beta subunit 1 | 8,60E-01 | 0,93 |
| ENSG00000111885 | MAN1A1 | mannosidase alpha class 1A member 1 | 1,00E+00 | 0,93 |
| ENSG00000270607 | AC009549.1 | novel transcript | 2,41E-01 | 0,92 |
| ENSG00000157111 | TMEM171 | transmembrane protein 171 | 7,96E-01 | 0,92 |
| ENSG00000258824 | AL122035.1 | novel transcript | 9,34E-01 | 0,90 |
| ENSG00000062582 | MRPS24 | mitochondrial ribosomal protein S24 | 6,85E-01 | 0,90 |
| ENSG00000236829 | Z97634.1 | uncharacterized LOC100134368 | 9,90E-01 | 0,89 |
| ENSG00000173926 | MARCH3 | membrane associated ring-CH-type finger 3 | 2,33E-01 | 0,89 |
| ENSG00000197208 | SLC22A4 | solute carrier family 22 member 4 | 6,43E-01 | 0,89 |
| ENSG00000086570 | FAT2 | FAT atypical cadherin 2 | 8,03E-01 | 0,89 |
| ENSG00000186665 | C17orf58 | chromosome 17 open reading frame 58 | 9,45E-01 | 0,88 |
| ENSG00000264947 | MIR3181 | microRNA 3181 | 1,00E+00 | 0,88 |
| ENSG00000125848 | FLRT3 | fibronectin leucine rich transmembrane protein 3 | 9,11E-01 | 0,88 |
| ENSG00000202474 | RNA5SP283 | RNA, 5S ribosomal pseudogene 283 | 7,96E-01 | 0,88 |
| ENSG00000008513 | ST3GAL1 | ST3 beta-galactoside alpha-2,3-sialyltransferase 1 | 3,77E-01 | 0,88 |
| ENSG00000064195 | DLX3 | distal-less homeobox 3 | 1,00E+00 | 0,87 |
| ENSG00000104147 | OIP5 | Opa interacting protein 5 | 1,00E+00 | 0,87 |
| ENSG00000170385 | SLC30A1 | solute carrier family 30 member 1 | 5,43E-03 | 0,87 |
| ENSG00000244586 | WNT5A-AS1 | WNT5A antisense RNA 1 | 9,49E-01 | 0,87 |
| ENSG00000101665 | SMAD7 | SMAD family member 7 | 3,47E-01 | 0,87 |
| ENSG00000204388 | HSPA1B | heat shock protein family A (Hsp70) member 1B | 7,28E-01 | 0,86 |
| ENSG00000274213 | AC015912.3 | novel transcript | 7,73E-01 | 0,85 |
| ENSG00000160326 | SLC2A6 | solute carrier family 2 member 6 | 8,54E-01 | 0,85 |
| ENSG00000273472 | AC096733.2 | novel transcript | 5,50E-01 | 0,85 |
| ENSG00000167604 | NFKBID | NFKB inhibitor delta | 3,37E-01 | 0,85 |
| ENSG00000158201 | ABHD3 | abhydrolase domain containing 3 | 5,49E-02 | 0,85 |
| ENSG00000214900 | LINC01588 | long intergenic non-protein coding RNA 1588 | 5,01E-01 | 0,84 |
| ENSG00000119714 | GPR68 | G protein-coupled receptor 68 | 1,00E+00 | 0,84 |
| ENSG00000178860 | MSC | musculin | 1,80E-01 | 0,83 |
| ENSG00000237596 | AL138828.1 | novel transcript | 5,07E-01 | 0,83 |
| ENSG00000154493 | C10orf90 | chromosome 10 open reading frame 90 | 7,32E-01 | 0,82 |
| **gene_id** | **gene_name** | **gene_description** | **padj** | **log2FoldChange** |
| ENSG00000260160 | AC011468.1 | novel transcript | 1,00E+00 | 0,81 |
| ENSG00000103995 | CEP152 | centrosomal protein 152 | 9,60E-01 | 0,81 |
| ENSG00000135678 | CPM | carboxypeptidase M | 9,84E-01 | 0,81 |
| ENSG00000101447 | FAM83D | family with sequence similarity 83 member D | 1,00E+00 | 0,80 |
| ENSG00000159110 | IFNAR2 | interferon alpha and beta receptor subunit 2 | 9,84E-01 | 0,80 |
| ENSG00000110911 | SLC11A2 | solute carrier family 11 member 2 | 2,91E-01 | 0,79 |
| ENSG00000085662 | AKR1B1 | aldo-keto reductase family 1 member B | 1,00E+00 | 0,79 |
| ENSG00000089351 | GRAMD1A | GRAM domain containing 1A | 6,23E-01 | 0,79 |
| ENSG00000253276 | CCDC71L | coiled-coil domain containing 71 like | 9,84E-01 | 0,79 |
| ENSG00000269982 | AC018809.2 | novel transcript, antisense to TMEM111 | 3,59E-01 | 0,76 |
| ENSG00000178878 | APOLD1 | apolipoprotein L domain containing 1 | 7,91E-01 | 0,76 |
| ENSG00000165899 | OTOGL | otogelin like | 9,15E-01 | 0,76 |
| ENSG00000162493 | PDPN | podoplanin | 8,82E-01 | 0,75 |
| ENSG00000138411 | HECW2 | HECT, C2 and WW domain containing E3 ubiquitin protein ligase 2 | 4,94E-01 | 0,75 |
| ENSG00000120051 | CFAP58 | cilia and flagella associated protein 58 | 1,00E+00 | 0,75 |
| ENSG00000274818 | AC004825.2 | novel transcript | 8,54E-01 | 0,74 |
| ENSG00000077150 | NFKB2 | nuclear factor kappa B subunit 2 | 9,71E-03 | 0,74 |
| ENSG00000226564 | FTH1P20 | ferritin heavy chain 1 pseudogene 20 | 8,03E-01 | 0,74 |
| ENSG00000149289 | ZC3H12C | zinc finger CCCH-type containing 12C | 6,01E-02 | 0,74 |
| ENSG00000129646 | QRICH2 | glutamine rich 2 | 1,00E+00 | 0,74 |
| ENSG00000178381 | ZFAND2A | zinc finger AN1-type containing 2A | 7,70E-01 | 0,74 |
| ENSG00000146409 | SLC18B1 | solute carrier family 18 member B1 | 1,00E+00 | 0,73 |
| ENSG00000269973 | AC010969.2 | novel transcript | 1,00E+00 | 0,73 |
| ENSG00000104856 | RELB | RELB proto-oncogene, NF-kB subunit | 2,26E-01 | 0,73 |
| ENSG00000167034 | NKX3-1 | NK3 homeobox 1 | 1,85E-01 | 0,73 |
| ENSG00000023909 | GCLM | glutamate-cysteine ligase modifier subunit | 3,34E-02 | 0,72 |
| ENSG00000056558 | TRAF1 | TNF receptor associated factor 1 | 8,88E-01 | 0,71 |
| ENSG00000237126 | AC073254.1 | novel transcript | 9,56E-01 | 0,71 |
| ENSG00000072571 | HMMR | hyaluronan mediated motility receptor | 9,94E-01 | 0,71 |
| ENSG00000198743 | SLC5A3 | solute carrier family 5 member 3 | 5,98E-01 | 0,71 |
| ENSG00000167703 | SLC43A2 | solute carrier family 43 member 2 | 3,84E-01 | 0,70 |
| ENSG00000121621 | KIF18A | kinesin family member 18A | 4,73E-01 | 0,70 |
| ENSG00000235919 | ASH1L-AS1 | ASH1L antisense RNA 1 | 9,84E-01 | 0,70 |
| ENSG00000228063 | LYPLAL1-DT | LYPLAL1 divergent transcript | 8,00E-01 | 0,69 |
| ENSG00000232973 | CYP1B1-AS1 | CYP1B1 antisense RNA 1 | 1,00E+00 | 0,69 |
| ENSG00000151136 | BTBD11 | BTB domain containing 11 | 6,07E-01 | 0,69 |
| ENSG00000139618 | BRCA2 | BRCA2, DNA repair associated | 9,45E-01 | 0,69 |
| ENSG00000185022 | MAFF | MAF bZIP transcription factor F | 5,67E-01 | 0,68 |
| **gene_id** | **gene_name** | **gene_description** | **padj** | **log2FoldChange** |
| ENSG00000100583 | SAMD15 | sterile alpha motif domain containing 15 | 1,00E+00 | 0,68 |
| ENSG00000163251 | FZD5 | frizzled class receptor 5 | 5,85E-01 | 0,68 |
| ENSG00000228327 | AL669831.1 | general transcription factor IIi (GTF2I) pseudogene | 5,38E-01 | 0,68 |
| ENSG00000171310 | CHST11 | carbohydrate sulfotransferase 11 | 9,39E-01 | 0,68 |
| ENSG00000278948 | AL031587.5 | TEC | 7,60E-01 | 0,67 |
| ENSG00000138386 | NAB1 | NGFI-A binding protein 1 | 4,75E-01 | 0,66 |
| ENSG00000125347 | IRF1 | interferon regulatory factor 1 | 9,64E-01 | 0,66 |
| ENSG00000113448 | PDE4D | phosphodiesterase 4D | 9,64E-01 | 0,66 |
| ENSG00000124593 | AL365205.1 | novel transcript | 9,00E-01 | 0,66 |
| ENSG00000185215 | TNFAIP2 | TNF alpha induced protein 2 | 8,54E-01 | 0,66 |
| ENSG00000187134 | AKR1C1 | aldo-keto reductase family 1 member C1 | 7,00E-01 | 0,64 |
| ENSG00000204516 | MICB | MHC class I polypeptide-related sequence B | 1,00E+00 | 0,64 |
| ENSG00000029153 | ARNTL2 | aryl hydrocarbon receptor nuclear translocator like 2 | 9,49E-01 | 0,64 |
| ENSG00000133657 | ATP13A3 | ATPase 13A3 | 4,12E-01 | 0,64 |
| ENSG00000185947 | ZNF267 | zinc finger protein 267 | 9,71E-03 | 0,64 |
| ENSG00000146232 | NFKBIE | NFKB inhibitor epsilon | 1,11E-01 | 0,63 |
| ENSG00000130066 | SAT1 | spermidine/spermine N1-acetyltransferase 1 | 7,00E-01 | 0,63 |
| ENSG00000234975 | FTH1P2 | ferritin heavy chain 1 pseudogene 2 | 9,40E-01 | 0,63 |
| ENSG00000150773 | PIH1D2 | PIH1 domain containing 2 | 7,60E-01 | 0,62 |
| ENSG00000233621 | LINC01137 | long intergenic non-protein coding RNA 1137 | 8,48E-02 | 0,62 |
| ENSG00000172216 | CEBPB | CCAAT enhancer binding protein beta | 5,07E-01 | 0,62 |
| ENSG00000232187 | FTH1P7 | ferritin heavy chain 1 pseudogene 7 | 9,88E-01 | 0,62 |
| ENSG00000071539 | TRIP13 | thyroid hormone receptor interactor 13 | 9,00E-01 | 0,61 |
| ENSG00000131669 | NINJ1 | ninjurin 1 | 1,00E+00 | 0,61 |
| ENSG00000159399 | HK2 | hexokinase 2 | 1,36E-01 | 0,60 |
| ENSG00000104419 | NDRG1 | N-myc downstream regulated 1 | 2,98E-01 | 0,60 |
| ENSG00000188993 | LRRC66 | leucine rich repeat containing 66 | 9,02E-01 | 0,60 |
| ENSG00000096968 | JAK2 | Janus kinase 2 | 9,15E-01 | 0,60 |
| ENSG00000163738 | MTHFD2L | methylenetetrahydrofolate dehydrogenase (NADP+ dependent) 2 like | 7,60E-01 | 0,59 |
| ENSG00000175352 | NRIP3 | nuclear receptor interacting protein 3 | 3,66E-01 | 0,59 |
| ENSG00000105643 | ARRDC2 | arrestin domain containing 2 | 9,43E-01 | 0,58 |
| ENSG00000141664 | ZCCHC2 | zinc finger CCHC-type containing 2 | 7,70E-01 | 0,57 |
| ENSG00000138835 | RGS3 | regulator of G protein signaling 3 | 3,18E-01 | 0,57 |
| ENSG00000269958 | AL049840.4 | novel transcript, sense intronic to KLC1 | 5,36E-02 | 0,57 |
| ENSG00000170525 | PFKFB3 | 6-phosphofructo-2-kinase/fructose-2,6-biphosphatase 3 | 7,32E-01 | 0,57 |
| ENSG00000091129 | NRCAM | neuronal cell adhesion molecule | 9,98E-01 | 0,57 |
| ENSG00000149177 | PTPRJ | protein tyrosine phosphatase, receptor type J | 7,96E-01 | 0,56 |
| ENSG00000131435 | PDLIM4 | PDZ and LIM domain 4 | 6,76E-01 | 0,56 |
| **gene_id** | **gene_name** | **gene_description** | **padj** | **log2FoldChange** |
| ENSG00000085117 | CD82 | CD82 molecule | 7,06E-01 | 0,56 |
| ENSG00000271614 | ATP2B1-AS1 | ATP2B1 antisense RNA 1 | 1,00E+00 | 0,56 |
| ENSG00000164970 | FAM219A | family with sequence similarity 219 member A | 9,21E-03 | 0,55 |
| ENSG00000134851 | TMEM165 | transmembrane protein 165 | 9,37E-01 | 0,55 |
| ENSG00000167996 | FTH1 | ferritin heavy chain 1 | 2,70E-01 | 0,55 |
| ENSG00000213066 | FGFR1OP | FGFR1 oncogene partner | 3,71E-01 | 0,55 |
| ENSG00000182791 | CCDC87 | coiled-coil domain containing 87 | 1,00E+00 | 0,55 |
| ENSG00000091073 | DTX2 | deltex E3 ubiquitin ligase 2 | 5,46E-01 | 0,54 |
| ENSG00000149485 | FADS1 | fatty acid desaturase 1 | 5,92E-01 | 0,54 |
| ENSG00000276728 | AC142472.1 | novel transcript | 1,00E+00 | 0,54 |
| ENSG00000121068 | TBX2 | T-box 2 | 2,90E-01 | 0,53 |
| ENSG00000240849 | TMEM189 | transmembrane protein 189 | 1,08E-01 | 0,53 |
| ENSG00000108179 | PPIF | peptidylprolyl isomerase F | 5,27E-01 | 0,53 |
| ENSG00000183337 | BCOR | BCL6 corepressor | 9,84E-01 | 0,53 |
| ENSG00000197442 | MAP3K5 | mitogen-activated protein kinase kinase kinase 5 | 7,60E-01 | 0,52 |
| ENSG00000237989 | LINC01679 | long intergenic non-protein coding RNA 1679 | 1,00E+00 | 0,52 |
| ENSG00000177674 | AGTRAP | angiotensin II receptor associated protein | 9,56E-01 | 0,52 |
| ENSG00000166801 | FAM111A | family with sequence similarity 111 member A | 1,00E+00 | 0,52 |
| ENSG00000003402 | CFLAR | CASP8 and FADD like apoptosis regulator | 2,45E-01 | 0,51 |
| ENSG00000118804 | STBD1 | starch binding domain 1 | 9,94E-01 | 0,51 |
| ENSG00000089775 | ZBTB25 | zinc finger and BTB domain containing 25 | 5,27E-01 | 0,51 |
| ENSG00000271383 | NBPF19 | NBPF member 19 | 9,11E-01 | 0,51 |
| ENSG00000174799 | CEP135 | centrosomal protein 135 | 7,96E-01 | 0,51 |
| ENSG00000125319 | C17orf53 | chromosome 17 open reading frame 53 | 5,33E-01 | 0,51 |
| ENSG00000198018 | ENTPD7 | ectonucleoside triphosphate diphosphohydrolase 7 | 9,48E-01 | 0,51 |
| ENSG00000184014 | DENND5A | DENN domain containing 5A | 4,21E-01 | 0,50 |
| ENSG00000127920 | GNG11 | G protein subunit gamma 11 | 9,94E-01 | 0,50 |
| ENSG00000221926 | TRIM16 | tripartite motif containing 16 | 8,54E-01 | 0,50 |
| ENSG00000162924 | REL | REL proto-oncogene, NF-kB subunit | 1,00E+00 | 0,49 |
| ENSG00000109436 | TBC1D9 | TBC1 domain family member 9 | 3,61E-01 | 0,49 |
| ENSG00000169330 | KIAA1024 | KIAA1024 | 6,93E-01 | 0,48 |
| ENSG00000112137 | PHACTR1 | phosphatase and actin regulator 1 | 8,13E-01 | 0,48 |
| ENSG00000134057 | CCNB1 | cyclin B1 | 1,00E+00 | 0,48 |
| ENSG00000091651 | ORC6 | origin recognition complex subunit 6 | 8,50E-01 | 0,48 |
| ENSG00000120910 | PPP3CC | protein phosphatase 3 catalytic subunit gamma | 9,66E-02 | 0,48 |
| ENSG00000267493 | CIRBP-AS1 | CIRBP antisense RNA 1 | 7,06E-01 | 0,47 |
| ENSG00000269439 | AC010618.3 | novel transcript | 9,02E-01 | 0,47 |
| ENSG00000117228 | GBP1 | guanylate binding protein 1 | 9,98E-01 | 0,47 |
| **gene_id** | **gene_name** | **gene_description** | **padj** | **log2FoldChange** |
| ENSG00000171608 | PIK3CD | phosphatidylinositol-4,5-bisphosphate 3-kinase catalytic subunit delta | 9,40E-01 | 0,47 |
| ENSG00000161011 | SQSTM1 | sequestosome 1 | 7,22E-02 | 0,46 |
| ENSG00000135837 | CEP350 | centrosomal protein 350 | 1,06E-01 | 0,46 |
| ENSG00000125629 | INSIG2 | insulin induced gene 2 | 6,30E-01 | 0,46 |
| ENSG00000136824 | SMC2 | structural maintenance of chromosomes 2 | 4,75E-01 | 0,46 |
| ENSG00000112319 | EYA4 | EYA transcriptional coactivator and phosphatase 4 | 1,00E+00 | 0,46 |
| ENSG00000171444 | MCC | MCC, WNT signaling pathway regulator | 9,15E-01 | 0,46 |
| ENSG00000177409 | SAMD9L | sterile alpha motif domain containing 9 like | 7,60E-01 | 0,45 |
| ENSG00000178694 | NSUN3 | NOP2/Sun RNA methyltransferase family member 3 | 5,85E-02 | 0,45 |
| ENSG00000178607 | ERN1 | endoplasmic reticulum to nucleus signaling 1 | 4,64E-01 | 0,44 |
| ENSG00000138182 | KIF20B | kinesin family member 20B | 1,00E+00 | 0,44 |
| ENSG00000138166 | DUSP5 | dual specificity phosphatase 5 | 9,45E-01 | 0,44 |
| ENSG00000128512 | DOCK4 | dedicator of cytokinesis 4 | 9,64E-01 | 0,44 |
| ENSG00000168209 | DDIT4 | DNA damage inducible transcript 4 | 4,64E-01 | 0,43 |
| ENSG00000254470 | AP5B1 | adaptor related protein complex 5 subunit beta 1 | 7,92E-01 | 0,43 |
| ENSG00000154640 | BTG3 | BTG anti-proliferation factor 3 | 5,62E-01 | 0,43 |
| ENSG00000111674 | ENO2 | enolase 2 | 9,15E-01 | 0,43 |
| ENSG00000108175 | ZMIZ1 | zinc finger MIZ-type containing 1 | 9,85E-01 | 0,43 |
| ENSG00000131747 | TOP2A | DNA topoisomerase II alpha | 9,27E-01 | 0,43 |
| ENSG00000083223 | TUT7 | terminal uridylyl transferase 7 | 1,17E-01 | 0,42 |
| ENSG00000256235 | SMIM3 | small integral membrane protein 3 | 3,77E-01 | 0,42 |
| ENSG00000176171 | BNIP3 | BCL2 interacting protein 3 | 8,54E-01 | 0,41 |
| ENSG00000059145 | UNKL | unkempt family like zinc finger | 4,26E-01 | 0,41 |
| ENSG00000241316 | SUCLG2-AS1 | SUCLG2 antisense RNA 1 (head to head) | 7,61E-01 | 0,40 |
| ENSG00000104312 | RIPK2 | receptor interacting serine/threonine kinase 2 | 1,00E+00 | 0,40 |
| ENSG00000221990 | EXOC3-AS1 | EXOC3 antisense RNA 1 | 1,00E+00 | 0,40 |
| ENSG00000115963 | RND3 | Rho family GTPase 3 | 8,88E-01 | 0,40 |
| ENSG00000135241 | PNPLA8 | patatin like phospholipase domain containing 8 | 8,02E-02 | 0,40 |
| ENSG00000189308 | LIN54 | lin-54 DREAM MuvB core complex component | 1,36E-04 | 0,40 |
| ENSG00000161791 | FMNL3 | formin like 3 | 1,00E+00 | 0,39 |
| ENSG00000015475 | BID | BH3 interacting domain death agonist | 5,22E-01 | 0,38 |
| ENSG00000010818 | HIVEP2 | human immunodeficiency virus type I enhancer binding protein 2 | 2,32E-01 | 0,38 |
| ENSG00000196396 | PTPN1 | protein tyrosine phosphatase, non-receptor type 1 | 1,17E-02 | 0,38 |
| ENSG00000086061 | DNAJA1 | DnaJ heat shock protein family (Hsp40) member A1 | 5,49E-02 | 0,38 |
| ENSG00000131323 | TRAF3 | TNF receptor associated factor 3 | 2,27E-01 | 0,38 |
| ENSG00000182481 | KPNA2 | karyopherin subunit alpha 2 | 7,60E-01 | 0,38 |
| ENSG00000171451 | DSEL | dermatan sulfate epimerase like | 1,00E+00 | 0,38 |
| ENSG00000228794 | LINC01128 | long intergenic non-protein coding RNA 1128 | 1,00E+00 | 0,38 |
| **gene_id** | **gene_name** | **gene_description** | **padj** | **log2FoldChange** |
| ENSG00000109320 | NFKB1 | nuclear factor kappa B subunit 1 | 9,50E-01 | 0,37 |
| ENSG00000135912 | TTLL4 | tubulin tyrosine ligase like 4 | 5,17E-01 | 0,37 |
| ENSG00000135541 | AHI1 | Abelson helper integration site 1 | 3,18E-01 | 0,36 |
| ENSG00000163961 | RNF168 | ring finger protein 168 | 7,60E-01 | 0,36 |
| ENSG00000269743 | SLC25A53 | solute carrier family 25 member 53 | 9,39E-01 | 0,36 |
| ENSG00000135766 | EGLN1 | egl-9 family hypoxia inducible factor 1 | 4,85E-01 | 0,36 |
| ENSG00000131626 | PPFIA1 | PTPRF interacting protein alpha 1 | 7,91E-03 | 0,36 |
| ENSG00000170485 | NPAS2 | neuronal PAS domain protein 2 | 9,50E-01 | 0,36 |
| ENSG00000129667 | RHBDF2 | rhomboid 5 homolog 2 | 6,05E-01 | 0,36 |
| ENSG00000197013 | ZNF429 | zinc finger protein 429 | 8,57E-01 | 0,36 |
| ENSG00000126870 | WDR60 | WD repeat domain 60 | 9,56E-01 | 0,35 |
| ENSG00000072274 | TFRC | transferrin receptor | 1,00E+00 | 0,35 |
| ENSG00000127481 | UBR4 | ubiquitin protein ligase E3 component n-recognin 4 | 2,77E-01 | 0,35 |
| ENSG00000132436 | FIGNL1 | fidgetin like 1 | 1,00E+00 | 0,35 |
| ENSG00000120647 | CCDC77 | coiled-coil domain containing 77 | 7,96E-01 | 0,35 |
| ENSG00000073150 | PANX2 | pannexin 2 | 9,64E-01 | 0,35 |
| ENSG00000023330 | ALAS1 | 5'-aminolevulinate synthase 1 | 7,26E-01 | 0,35 |
| ENSG00000144597 | EAF1 | ELL associated factor 1 | 8,22E-01 | 0,34 |
| ENSG00000118985 | ELL2 | elongation factor for RNA polymerase II 2 | 3,18E-01 | 0,34 |
| ENSG00000235363 | SNRPGP10 | small nuclear ribonucleoprotein polypeptide G pseudogene 10 | 1,00E+00 | 0,34 |
| ENSG00000149231 | CCDC82 | coiled-coil domain containing 82 | 1,16E-01 | 0,34 |
| ENSG00000143971 | ETAA1 | ETAA1, ATR kinase activator | 7,60E-01 | 0,34 |
| ENSG00000167987 | VPS37C | VPS37C, ESCRT-I subunit | 2,99E-01 | 0,34 |
| ENSG00000198604 | BAZ1A | bromodomain adjacent to zinc finger domain 1A | 5,96E-01 | 0,34 |
| ENSG00000176788 | BASP1 | brain abundant membrane attached signal protein 1 | 1,00E+00 | 0,34 |
| ENSG00000156313 | RPGR | retinitis pigmentosa GTPase regulator | 8,87E-01 | 0,34 |
| ENSG00000101945 | SUV39H1 | suppressor of variegation 3-9 homolog 1 | 1,00E+00 | 0,34 |
| ENSG00000116793 | PHTF1 | putative homeodomain transcription factor 1 | 2,98E-01 | 0,34 |
| ENSG00000127080 | IPPK | inositol-pentakisphosphate 2-kinase | 1,00E+00 | 0,34 |
| ENSG00000204209 | DAXX | death domain associated protein | 9,40E-01 | 0,33 |
| ENSG00000139112 | GABARAPL1 | GABA type A receptor associated protein like 1 | 1,00E+00 | 0,33 |
| ENSG00000260267 | AC026471.1 | novel transcript, antisense to ARMC5 | 5,98E-01 | 0,33 |
| ENSG00000225614 | ZNF469 | zinc finger protein 469 | 9,50E-01 | 0,33 |
| ENSG00000173276 | ZBTB21 | zinc finger and BTB domain containing 21 | 3,77E-01 | 0,33 |
| ENSG00000136147 | PHF11 | PHD finger protein 11 | 1,00E+00 | 0,33 |
| ENSG00000125037 | EMC3 | ER membrane protein complex subunit 3 | 4,47E-01 | 0,32 |
| ENSG00000251136 | AF117829.1 | uncharacterized LOC101929709 | 9,88E-01 | 0,32 |
| ENSG00000122970 | IFT81 | intraflagellar transport 81 | 9,90E-01 | 0,32 |
| **gene_id** | **gene_name** | **gene_description** | **padj** | **log2FoldChange** |
| ENSG00000181220 | ZNF746 | zinc finger protein 746 | 1,00E+00 | 0,32 |
| ENSG00000100139 | MICALL1 | MICAL like 1 | 8,31E-01 | 0,32 |
| ENSG00000172493 | AFF1 | AF4/FMR2 family member 1 | 1,00E+00 | 0,32 |
| ENSG00000167995 | BEST1 | bestrophin 1 | 1,00E+00 | 0,32 |
| ENSG00000123136 | DDX39A | DExD-box helicase 39A | 9,73E-01 | 0,32 |
| ENSG00000100647 | SUSD6 | sushi domain containing 6 | 9,84E-01 | 0,31 |
| ENSG00000143479 | DYRK3 | dual specificity tyrosine phosphorylation regulated kinase 3 | 1,00E+00 | 0,31 |
| ENSG00000124588 | NQO2 | N-ribosyldihydronicotinamide:quinone reductase 2 | 1,00E+00 | 0,31 |
| ENSG00000157224 | CLDN12 | claudin 12 | 1,00E+00 | 0,31 |
| ENSG00000001084 | GCLC | glutamate-cysteine ligase catalytic subunit | 8,51E-01 | 0,31 |
| ENSG00000099290 | WASHC2A | WASH complex subunit 2A | 6,44E-01 | 0,31 |
| ENSG00000168394 | TAP1 | transporter 1, ATP binding cassette subfamily B member | 5,80E-01 | 0,31 |
| ENSG00000102804 | TSC22D1 | TSC22 domain family member 1 | 9,11E-01 | 0,31 |
| ENSG00000157625 | TAB3 | TGF-beta activated kinase 1 (MAP3K7) binding protein 3 | 2,26E-02 | 0,31 |
| ENSG00000136810 | TXN | thioredoxin | 8,88E-01 | 0,30 |
| ENSG00000108395 | TRIM37 | tripartite motif containing 37 | 3,00E-01 | 0,30 |
| ENSG00000012211 | PRICKLE3 | prickle planar cell polarity protein 3 | 9,88E-01 | 0,30 |
| ENSG00000113522 | RAD50 | RAD50 double strand break repair protein | 1,00E+00 | 0,30 |
| ENSG00000130779 | CLIP1 | CAP-Gly domain containing linker protein 1 | 5,80E-01 | 0,30 |
| ENSG00000174276 | ZNHIT2 | zinc finger HIT-type containing 2 | 6,30E-01 | 0,30 |
| ENSG00000159884 | CCDC107 | coiled-coil domain containing 107 | 9,98E-01 | 0,29 |
| ENSG00000197860 | SGTB | small glutamine rich tetratricopeptide repeat containing beta | 8,25E-01 | 0,29 |
| ENSG00000169594 | BNC1 | basonuclin 1 | 9,40E-01 | 0,29 |
| ENSG00000145780 | FEM1C | fem-1 homolog C | 4,15E-01 | 0,29 |
| ENSG00000137776 | SLTM | SAFB like transcription modulator | 9,39E-01 | 0,29 |
| ENSG00000011007 | ELOA | elongin A | 3,19E-01 | 0,28 |
| ENSG00000100567 | PSMA3 | proteasome subunit alpha 3 | 5,33E-01 | 0,28 |
| ENSG00000148154 | UGCG | UDP-glucose ceramide glucosyltransferase | 9,45E-01 | 0,28 |
| ENSG00000184831 | APOO | apolipoprotein O | 9,40E-01 | 0,28 |
| ENSG00000065833 | ME1 | malic enzyme 1 | 8,81E-01 | 0,28 |
| ENSG00000148634 | HERC4 | HECT and RLD domain containing E3 ubiquitin protein ligase 4 | 1,00E+00 | 0,28 |
| ENSG00000116977 | LGALS8 | galectin 8 | 1,00E+00 | 0,27 |
| ENSG00000148331 | ASB6 | ankyrin repeat and SOCS box containing 6 | 9,39E-01 | 0,27 |
| ENSG00000023287 | RB1CC1 | RB1 inducible coiled-coil 1 | 8,54E-01 | 0,27 |
| ENSG00000008294 | SPAG9 | sperm associated antigen 9 | 6,78E-01 | 0,27 |
| ENSG00000127838 | PNKD | PNKD, MBL domain containing | 1,00E+00 | 0,26 |
| ENSG00000114480 | GBE1 | 1,4-alpha-glucan branching enzyme 1 | 1,00E+00 | 0,26 |
| ENSG00000115652 | UXS1 | UDP-glucuronate decarboxylase 1 | 9,90E-01 | 0,25 |
| **gene_id** | **gene_name** | **gene_description** | **padj** | **log2FoldChange** |
| ENSG00000102543 | CDADC1 | cytidine and dCMP deaminase domain containing 1 | 4,12E-01 | 0,25 |
| ENSG00000188786 | MTF1 | metal regulatory transcription factor 1 | 1,00E+00 | 0,25 |
| ENSG00000130347 | RTN4IP1 | reticulon 4 interacting protein 1 | 9,48E-01 | 0,25 |
| ENSG00000094975 | SUCO | SUN domain containing ossification factor | 4,19E-01 | 0,25 |
| ENSG00000183735 | TBK1 | TANK binding kinase 1 | 1,89E-01 | 0,25 |
| ENSG00000145734 | BDP1 | B double prime 1, subunit of RNA polymerase III transcription initiation factor IIIB | 7,49E-01 | 0,24 |
| ENSG00000124789 | NUP153 | nucleoporin 153 | 1,65E-01 | 0,24 |
| ENSG00000141452 | RMC1 | regulator of MON1-CCZ1 | 5,92E-01 | 0,24 |
| ENSG00000075292 | ZNF638 | zinc finger protein 638 | 5,37E-01 | 0,23 |
| ENSG00000132471 | WBP2 | WW domain binding protein 2 | 6,32E-01 | 0,23 |
| ENSG00000114573 | ATP6V1A | ATPase H+ transporting V1 subunit A | 1,00E+00 | 0,23 |
| ENSG00000132823 | OSER1 | oxidative stress responsive serine rich 1 | 9,84E-01 | 0,23 |
| ENSG00000171206 | TRIM8 | tripartite motif containing 8 | 8,54E-01 | 0,23 |
| ENSG00000100416 | TRMU | tRNA 5-methylaminomethyl-2-thiouridylate methyltransferase | 4,25E-01 | 0,23 |
| ENSG00000278318 | ZNF229 | zinc finger protein 229 | 6,78E-01 | 0,23 |
| ENSG00000180992 | MRPL14 | mitochondrial ribosomal protein L14 | 1,00E+00 | 0,23 |
| ENSG00000118620 | ZNF430 | zinc finger protein 430 | 1,00E+00 | 0,23 |
| ENSG00000130772 | MED18 | mediator complex subunit 18 | 8,88E-01 | 0,22 |
| ENSG00000047249 | ATP6V1H | ATPase H+ transporting V1 subunit H | 5,50E-01 | 0,22 |
| ENSG00000197217 | ENTPD4 | ectonucleoside triphosphate diphosphohydrolase 4 | 9,40E-01 | 0,22 |
| ENSG00000155975 | VPS37A | VPS37A, ESCRT-I subunit | 5,38E-01 | 0,22 |
| ENSG00000122965 | RBM19 | RNA binding motif protein 19 | 6,73E-01 | 0,21 |
| ENSG00000198742 | SMURF1 | SMAD specific E3 ubiquitin protein ligase 1 | 9,84E-01 | 0,21 |
| ENSG00000066697 | MSANTD3 | Myb/SANT DNA binding domain containing 3 | 9,84E-01 | 0,21 |
| ENSG00000075413 | MARK3 | microtubule affinity regulating kinase 3 | 9,15E-01 | 0,21 |
| ENSG00000135018 | UBQLN1 | ubiquilin 1 | 8,87E-01 | 0,21 |
| ENSG00000106608 | URGCP | upregulator of cell proliferation | 4,94E-01 | 0,20 |
| ENSG00000087087 | SRRT | serrate, RNA effector molecule | 5,92E-01 | 0,20 |
| ENSG00000137710 | RDX | radixin | 9,40E-01 | 0,20 |
| ENSG00000101189 | MRGBP | MRG domain binding protein | 3,71E-01 | 0,20 |
| ENSG00000057757 | PITHD1 | PITH domain containing 1 | 7,60E-01 | 0,20 |
| ENSG00000011132 | APBA3 | amyloid beta precursor protein binding family A member 3 | 8,69E-01 | 0,20 |
| ENSG00000171928 | TVP23B | trans-golgi network vesicle protein 23 homolog B | 1,00E+00 | 0,20 |
| ENSG00000163516 | ANKZF1 | ankyrin repeat and zinc finger domain containing 1 | 6,73E-01 | 0,19 |
| ENSG00000127666 | TICAM1 | toll like receptor adaptor molecule 1 | 9,84E-01 | 0,19 |
| ENSG00000153922 | CHD1 | chromodomain helicase DNA binding protein 1 | 1,00E+00 | 0,19 |
| ENSG00000170142 | UBE2E1 | ubiquitin conjugating enzyme E2 E1 | 7,70E-01 | 0,19 |
| ENSG00000161847 | RAVER1 | ribonucleoprotein, PTB binding 1 | 9,88E-01 | 0,19 |
| **gene_id** | **gene_name** | **gene_description** | **padj** | **log2FoldChange** |
| ENSG00000111726 | CMAS | cytidine monophosphate N-acetylneuraminic acid synthetase | 9,98E-01 | 0,19 |
| ENSG00000110330 | BIRC2 | baculoviral IAP repeat containing 2 | 8,54E-01 | 0,18 |
| ENSG00000179335 | CLK3 | CDC like kinase 3 | 6,25E-01 | 0,18 |
| ENSG00000076108 | BAZ2A | bromodomain adjacent to zinc finger domain 2A | 1,00E+00 | 0,18 |
| ENSG00000130254 | SAFB2 | scaffold attachment factor B2 | 1,00E+00 | 0,18 |
| ENSG00000103042 | SLC38A7 | solute carrier family 38 member 7 | 9,84E-01 | 0,18 |
| ENSG00000198900 | TOP1 | DNA topoisomerase I | 2,04E-01 | 0,18 |
| ENSG00000148719 | DNAJB12 | DnaJ heat shock protein family (Hsp40) member B12 | 7,00E-01 | 0,17 |
| ENSG00000153560 | UBP1 | upstream binding protein 1 | 9,60E-01 | 0,17 |
| ENSG00000060339 | CCAR1 | cell division cycle and apoptosis regulator 1 | 5,37E-01 | 0,16 |
| ENSG00000173120 | KDM2A | lysine demethylase 2A | 7,60E-01 | 0,16 |
| ENSG00000160058 | BSDC1 | BSD domain containing 1 | 5,62E-01 | 0,16 |
| ENSG00000006831 | ADIPOR2 | adiponectin receptor 2 | 1,00E+00 | 0,16 |
| ENSG00000173039 | RELA | RELA proto-oncogene, NF-kB subunit | 5,37E-01 | 0,15 |
| ENSG00000136813 | ECPAS | Ecm29 proteasome adaptor and scaffold | 7,50E-01 | 0,15 |
| ENSG00000136754 | ABI1 | abl interactor 1 | 1,00E+00 | 0,14 |
| ENSG00000119321 | FKBP15 | FK506 binding protein 15 | 5,42E-01 | 0,14 |
| ENSG00000119844 | AFTPH | aftiphilin | 9,84E-01 | 0,14 |
| ENSG00000163681 | SLMAP | sarcolemma associated protein | 7,96E-01 | 0,14 |
| ENSG00000116266 | STXBP3 | syntaxin binding protein 3 | 9,94E-01 | 0,12 |
| ENSG00000114982 | KANSL3 | KAT8 regulatory NSL complex subunit 3 | 9,15E-01 | 0,11 |
| ENSG00000185787 | MORF4L1 | mortality factor 4 like 1 | 6,78E-01 | -0,10 |
| ENSG00000188529 | SRSF10 | serine and arginine rich splicing factor 10 | 5,98E-01 | -0,12 |
| ENSG00000105221 | AKT2 | AKT serine/threonine kinase 2 | 1,00E+00 | -0,12 |
| ENSG00000087274 | ADD1 | adducin 1 | 8,62E-01 | -0,13 |
| ENSG00000081760 | AACS | acetoacetyl-CoA synthetase | 8,21E-01 | -0,13 |
| ENSG00000215021 | PHB2 | prohibitin 2 | 9,84E-01 | -0,14 |
| ENSG00000276293 | PIP4K2B | phosphatidylinositol-5-phosphate 4-kinase type 2 beta | 1,00E+00 | -0,14 |
| ENSG00000168118 | RAB4A | RAB4A, member RAS oncogene family | 1,00E+00 | -0,15 |
| ENSG00000133884 | DPF2 | double PHD fingers 2 | 9,84E-01 | -0,15 |
| ENSG00000139579 | NABP2 | nucleic acid binding protein 2 | 7,60E-01 | -0,15 |
| ENSG00000132589 | FLOT2 | flotillin 2 | 9,94E-01 | -0,15 |
| ENSG00000124193 | SRSF6 | serine and arginine rich splicing factor 6 | 9,98E-01 | -0,15 |
| ENSG00000124172 | ATP5F1E | ATP synthase F1 subunit epsilon | 6,07E-01 | -0,16 |
| ENSG00000064102 | INTS13 | integrator complex subunit 13 | 9,94E-01 | -0,16 |
| ENSG00000179941 | BBS10 | Bardet-Biedl syndrome 10 | 1,00E+00 | -0,16 |
| ENSG00000113658 | SMAD5 | SMAD family member 5 | 4,26E-01 | -0,17 |
| ENSG00000141258 | SGSM2 | small G protein signaling modulator 2 | 8,03E-01 | -0,17 |
| **gene_id** | **gene_name** | **gene_description** | **padj** | **log2FoldChange** |
| ENSG00000175826 | CTDNEP1 | CTD nuclear envelope phosphatase 1 | 7,96E-01 | -0,17 |
| ENSG00000104375 | STK3 | serine/threonine kinase 3 | 8,06E-01 | -0,17 |
| ENSG00000178537 | SLC25A20 | solute carrier family 25 member 20 | 1,00E+00 | -0,17 |
| ENSG00000172943 | PHF8 | PHD finger protein 8 | 9,88E-01 | -0,17 |
| ENSG00000155229 | MMS19 | MMS19 homolog, cytosolic iron-sulfur assembly component | 5,59E-01 | -0,17 |
| ENSG00000089009 | RPL6 | ribosomal protein L6 | 8,79E-01 | -0,17 |
| ENSG00000136247 | ZDHHC4 | zinc finger DHHC-type containing 4 | 5,98E-01 | -0,17 |
| ENSG00000074201 | CLNS1A | chloride nucleotide-sensitive channel 1A | 4,94E-01 | -0,18 |
| ENSG00000177600 | RPLP2 | ribosomal protein lateral stalk subunit P2 | 1,00E+00 | -0,18 |
| ENSG00000117411 | B4GALT2 | beta-1,4-galactosyltransferase 2 | 5,50E-01 | -0,18 |
| ENSG00000175390 | EIF3F | eukaryotic translation initiation factor 3 subunit F | 8,35E-01 | -0,18 |
| ENSG00000099385 | BCL7C | BCL tumor suppressor 7C | 9,60E-01 | -0,18 |
| ENSG00000204498 | NFKBIL1 | NFKB inhibitor like 1 | 1,00E+00 | -0,19 |
| ENSG00000086200 | IPO11 | importin 11 | 1,00E+00 | -0,19 |
| ENSG00000163344 | PMVK | phosphomevalonate kinase | 9,40E-01 | -0,19 |
| ENSG00000184939 | ZFP90 | ZFP90 zinc finger protein | 7,65E-01 | -0,19 |
| ENSG00000104522 | TSTA3 | tissue specific transplantation antigen P35B | 6,82E-01 | -0,19 |
| ENSG00000171169 | NAIF1 | nuclear apoptosis inducing factor 1 | 1,00E+00 | -0,19 |
| ENSG00000100605 | ITPK1 | inositol-tetrakisphosphate 1-kinase | 6,42E-01 | -0,20 |
| ENSG00000130332 | LSM7 | LSM7 homolog, U6 small nuclear RNA and mRNA degradation associated | 1,00E+00 | -0,20 |
| ENSG00000151502 | VPS26B | VPS26, retromer complex component B | 4,94E-01 | -0,20 |
| ENSG00000145916 | RMND5B | required for meiotic nuclear division 5 homolog B | 9,09E-01 | -0,20 |
| ENSG00000084693 | AGBL5 | ATP/GTP binding protein like 5 | 6,30E-01 | -0,20 |
| ENSG00000175115 | PACS1 | phosphofurin acidic cluster sorting protein 1 | 1,00E+00 | -0,20 |
| ENSG00000215039 | CD27-AS1 | CD27 antisense RNA 1 | 9,48E-01 | -0,21 |
| ENSG00000135108 | FBXO21 | F-box protein 21 | 1,42E-01 | -0,21 |
| ENSG00000178397 | FAM220A | family with sequence similarity 220 member A | 7,61E-01 | -0,21 |
| ENSG00000204611 | ZNF616 | zinc finger protein 616 | 5,80E-01 | -0,21 |
| ENSG00000144647 | POMGNT2 | protein O-linked mannose N-acetylglucosaminyltransferase 2 (beta 1,4-) | 3,80E-01 | -0,21 |
| ENSG00000143314 | MRPL24 | mitochondrial ribosomal protein L24 | 1,00E+00 | -0,21 |
| ENSG00000170881 | RNF139 | ring finger protein 139 | 4,64E-01 | -0,22 |
| ENSG00000271601 | LIX1L | limb and CNS expressed 1 like | 5,98E-01 | -0,22 |
| ENSG00000073536 | NLE1 | notchless homolog 1 | 5,92E-01 | -0,22 |
| ENSG00000197928 | ZNF677 | zinc finger protein 677 | 9,39E-01 | -0,22 |
| ENSG00000116874 | WARS2 | tryptophanyl tRNA synthetase 2, mitochondrial | 1,00E+00 | -0,22 |
| ENSG00000114544 | SLC41A3 | solute carrier family 41 member 3 | 9,45E-01 | -0,23 |
| ENSG00000100417 | PMM1 | phosphomannomutase 1 | 7,00E-01 | -0,23 |
| ENSG00000069275 | NUCKS1 | nuclear casein kinase and cyclin dependent kinase substrate 1 | 1,11E-01 | -0,23 |
| **gene_id** | **gene_name** | **gene_description** | **padj** | **log2FoldChange** |
| ENSG00000139719 | VPS33A | VPS33A, CORVET/HOPS core subunit | 7,61E-01 | -0,23 |
| ENSG00000057935 | MTA3 | metastasis associated 1 family member 3 | 6,73E-01 | -0,23 |
| ENSG00000132879 | FBXO44 | F-box protein 44 | 1,00E+00 | -0,23 |
| ENSG00000128059 | PPAT | phosphoribosyl pyrophosphate amidotransferase | 5,92E-01 | -0,24 |
| ENSG00000100612 | DHRS7 | dehydrogenase/reductase 7 | 8,03E-02 | -0,24 |
| ENSG00000139641 | ESYT1 | extended synaptotagmin 1 | 1,00E+00 | -0,24 |
| ENSG00000183665 | TRMT12 | tRNA methyltransferase 12 homolog | 8,87E-01 | -0,24 |
| ENSG00000164904 | ALDH7A1 | aldehyde dehydrogenase 7 family member A1 | 1,52E-01 | -0,24 |
| ENSG00000092096 | SLC22A17 | solute carrier family 22 member 17 | 9,85E-01 | -0,24 |
| ENSG00000100320 | RBFOX2 | RNA binding fox-1 homolog 2 | 9,88E-01 | -0,24 |
| ENSG00000160917 | CPSF4 | cleavage and polyadenylation specific factor 4 | 4,64E-01 | -0,25 |
| ENSG00000175581 | MRPL48 | mitochondrial ribosomal protein L48 | 1,00E+00 | -0,25 |
| ENSG00000152382 | TADA1 | transcriptional adaptor 1 | 1,00E+00 | -0,25 |
| ENSG00000124641 | MED20 | mediator complex subunit 20 | 5,06E-01 | -0,25 |
| ENSG00000172667 | ZMAT3 | zinc finger matrin-type 3 | 9,15E-01 | -0,25 |
| ENSG00000010322 | NISCH | nischarin | 9,40E-01 | -0,26 |
| ENSG00000185305 | ARL15 | ADP ribosylation factor like GTPase 15 | 1,00E+00 | -0,26 |
| ENSG00000054793 | ATP9A | ATPase phospholipid transporting 9A (putative) | 5,50E-01 | -0,26 |
| ENSG00000110315 | RNF141 | ring finger protein 141 | 8,56E-01 | -0,26 |
| ENSG00000184584 | TMEM173 | transmembrane protein 173 | 4,94E-01 | -0,26 |
| ENSG00000160753 | RUSC1 | RUN and SH3 domain containing 1 | 9,84E-01 | -0,27 |
| ENSG00000206538 | VGLL3 | vestigial like family member 3 | 6,44E-01 | -0,27 |
| ENSG00000165698 | SPACA9 | sperm acrosome associated 9 | 7,96E-01 | -0,28 |
| ENSG00000172878 | METAP1D | methionyl aminopeptidase type 1D, mitochondrial | 6,53E-01 | -0,28 |
| ENSG00000133243 | BTBD2 | BTB domain containing 2 | 1,00E+00 | -0,28 |
| ENSG00000008441 | NFIX | nuclear factor I X | 1,00E+00 | -0,28 |
| ENSG00000165886 | UBTD1 | ubiquitin domain containing 1 | 1,00E+00 | -0,28 |
| ENSG00000166352 | C11orf74 | chromosome 11 open reading frame 74 | 9,98E-01 | -0,29 |
| ENSG00000187091 | PLCD1 | phospholipase C delta 1 | 6,98E-01 | -0,29 |
| ENSG00000169288 | MRPL1 | mitochondrial ribosomal protein L1 | 1,00E+00 | -0,29 |
| ENSG00000101220 | C20orf27 | chromosome 20 open reading frame 27 | 8,54E-01 | -0,29 |
| ENSG00000072195 | SPEG | SPEG complex locus | 9,88E-01 | -0,30 |
| ENSG00000134330 | IAH1 | isoamyl acetate hydrolyzing esterase 1 (putative) | 8,93E-01 | -0,30 |
| ENSG00000214078 | CPNE1 | copine 1 | 9,98E-01 | -0,30 |
| ENSG00000146416 | AIG1 | androgen induced 1 | 1,00E+00 | -0,30 |
| ENSG00000115425 | PECR | peroxisomal trans-2-enoyl-CoA reductase | 9,84E-01 | -0,31 |
| ENSG00000183828 | NUDT14 | nudix hydrolase 14 | 9,39E-01 | -0,31 |
| ENSG00000160789 | LMNA | lamin A/C | 1,00E+00 | -0,31 |
| **gene_id** | **gene_name** | **gene_description** | **padj** | **log2FoldChange** |
| ENSG00000196628 | TCF4 | transcription factor 4 | 4,94E-01 | -0,31 |
| ENSG00000240184 | PCDHGC3 | protocadherin gamma subfamily C, 3 | 1,00E+00 | -0,31 |
| ENSG00000167535 | CACNB3 | calcium voltage-gated channel auxiliary subunit beta 3 | 8,76E-01 | -0,32 |
| ENSG00000108479 | GALK1 | galactokinase 1 | 6,60E-01 | -0,33 |
| ENSG00000140931 | CMTM3 | CKLF like MARVEL transmembrane domain containing 3 | 5,80E-01 | -0,33 |
| ENSG00000131089 | ARHGEF9 | Cdc42 guanine nucleotide exchange factor 9 | 9,34E-01 | -0,33 |
| ENSG00000091831 | ESR1 | estrogen receptor 1 | 1,00E+00 | -0,33 |
| ENSG00000151376 | ME3 | malic enzyme 3 | 1,00E+00 | -0,33 |
| ENSG00000070882 | OSBPL3 | oxysterol binding protein like 3 | 8,87E-01 | -0,34 |
| ENSG00000172059 | KLF11 | Kruppel like factor 11 | 1,00E+00 | -0,34 |
| ENSG00000187626 | ZKSCAN4 | zinc finger with KRAB and SCAN domains 4 | 7,28E-01 | -0,34 |
| ENSG00000075618 | FSCN1 | fascin actin-bundling protein 1 | 9,98E-01 | -0,35 |
| ENSG00000120837 | NFYB | nuclear transcription factor Y subunit beta | 9,18E-01 | -0,35 |
| ENSG00000008382 | MPND | MPN domain containing | 4,90E-01 | -0,35 |
| ENSG00000170917 | NUDT6 | nudix hydrolase 6 | 1,00E+00 | -0,36 |
| ENSG00000125772 | GPCPD1 | glycerophosphocholine phosphodiesterase 1 | 4,95E-01 | -0,36 |
| ENSG00000109458 | GAB1 | GRB2 associated binding protein 1 | 1,00E+00 | -0,36 |
| ENSG00000196950 | SLC39A10 | solute carrier family 39 member 10 | 8,22E-01 | -0,36 |
| ENSG00000122694 | GLIPR2 | GLI pathogenesis related 2 | 8,54E-01 | -0,37 |
| ENSG00000042317 | SPATA7 | spermatogenesis associated 7 | 9,84E-01 | -0,37 |
| ENSG00000076003 | MCM6 | minichromosome maintenance complex component 6 | 5,92E-01 | -0,37 |
| ENSG00000120913 | PDLIM2 | PDZ and LIM domain 2 | 1,00E+00 | -0,37 |
| ENSG00000149269 | PAK1 | p21 (RAC1) activated kinase 1 | 1,00E+00 | -0,37 |
| ENSG00000181467 | RAP2B | RAP2B, member of RAS oncogene family | 3,58E-01 | -0,38 |
| ENSG00000137502 | RAB30 | RAB30, member RAS oncogene family | 9,27E-01 | -0,39 |
| ENSG00000005059 | MCUB | mitochondrial calcium uniporter dominant negative beta subunit | 3,00E-01 | -0,39 |
| ENSG00000008256 | CYTH3 | cytohesin 3 | 4,64E-01 | -0,39 |
| ENSG00000111203 | ITFG2 | integrin alpha FG-GAP repeat containing 2 | 3,26E-01 | -0,40 |
| ENSG00000283041 | AC008038.1 | eukaryotic translation elongation factor 1 gamma ( | 2,58E-01 | -0,41 |
| ENSG00000139174 | PRICKLE1 | prickle planar cell polarity protein 1 | 6,00E-01 | -0,42 |
| ENSG00000205208 | C4orf46 | chromosome 4 open reading frame 46 | 9,40E-01 | -0,44 |
| ENSG00000077684 | JADE1 | jade family PHD finger 1 | 5,81E-01 | -0,45 |
| ENSG00000154122 | ANKH | ANKH inorganic pyrophosphate transport regulator | 7,53E-01 | -0,47 |
| ENSG00000269893 | SNHG8 | small nucleolar RNA host gene 8 | 9,84E-01 | -0,49 |
| ENSG00000125510 | OPRL1 | opioid related nociceptin receptor 1 | 1,00E+00 | -0,49 |
| ENSG00000151090 | THRB | thyroid hormone receptor beta | 1,42E-01 | -0,50 |
| ENSG00000090776 | EFNB1 | ephrin B1 | 6,71E-01 | -0,50 |
| ENSG00000242732 | RTL5 | retrotransposon Gag like 5 | 5,27E-01 | -0,50 |
| **gene_id** | **gene_name** | **gene_description** | **padj** | **log2FoldChange** |
| ENSG00000136114 | THSD1 | thrombospondin type 1 domain containing 1 | 7,96E-01 | -0,51 |
| ENSG00000188672 | RHCE | Rh blood group CcEe antigens | 9,84E-01 | -0,51 |
| ENSG00000189283 | FHIT | fragile histidine triad | 9,84E-01 | -0,52 |
| ENSG00000126603 | GLIS2 | GLIS family zinc finger 2 | 6,25E-01 | -0,52 |
| ENSG00000171962 | DRC3 | dynein regulatory complex subunit 3 | 1,00E+00 | -0,53 |
| ENSG00000141540 | TTYH2 | tweety family member 2 | 1,00E+00 | -0,53 |
| ENSG00000169439 | SDC2 | syndecan 2 | 3,53E-03 | -0,53 |
| ENSG00000030419 | IKZF2 | IKAROS family zinc finger 2 | 4,94E-01 | -0,55 |
| ENSG00000145604 | SKP2 | S-phase kinase associated protein 2 | 3,19E-01 | -0,55 |
| ENSG00000161513 | FDXR | ferredoxin reductase | 7,60E-01 | -0,55 |
| ENSG00000173947 | PIFO | primary cilia formation | 7,42E-01 | -0,55 |
| ENSG00000273329 | AC078846.1 | novel transcript | 6,07E-01 | -0,56 |
| ENSG00000011332 | DPF1 | double PHD fingers 1 | 9,84E-01 | -0,56 |
| ENSG00000156299 | TIAM1 | T cell lymphoma invasion and metastasis 1 | 8,54E-01 | -0,56 |
| ENSG00000149582 | TMEM25 | transmembrane protein 25 | 1,00E+00 | -0,57 |
| ENSG00000257337 | AC068888.1 | uncharacterized LOC283335 | 6,60E-01 | -0,57 |
| ENSG00000245213 | AC105285.1 | uncharacterized LOC101930370 | 5,07E-01 | -0,57 |
| ENSG00000007944 | MYLIP | myosin regulatory light chain interacting protein | 5,98E-01 | -0,58 |
| ENSG00000246273 | SBF2-AS1 | SBF2 antisense RNA 1 | 5,92E-01 | -0,58 |
| ENSG00000147642 | SYBU | syntabulin | 6,43E-01 | -0,58 |
| ENSG00000213866 | YBX1P10 | Y-box binding protein 1 pseudogene 10 | 5,62E-01 | -0,59 |
| ENSG00000105672 | ETV2 | ETS variant 2 | 8,22E-01 | -0,61 |
| ENSG00000185774 | KCNIP4 | potassium voltage-gated channel interacting protein 4 | 1,00E+00 | -0,61 |
| ENSG00000272455 | AL391244.3 | novel transcript | 7,60E-01 | -0,62 |
| ENSG00000101384 | JAG1 | jagged 1 | 1,00E+00 | -0,62 |
| ENSG00000205930 | C21orf62-AS1 | C21orf62 antisense RNA 1 | 9,21E-01 | -0,62 |
| ENSG00000198774 | RASSF9 | Ras association domain family member 9 | 5,33E-01 | -0,64 |
| ENSG00000176472 | ZNF575 | zinc finger protein 575 | 9,73E-01 | -0,66 |
| ENSG00000239523 | MYLK-AS1 | MYLK antisense RNA 1 | 9,84E-01 | -0,66 |
| ENSG00000167772 | ANGPTL4 | angiopoietin like 4 | 9,55E-01 | -0,66 |
| ENSG00000144730 | IL17RD | interleukin 17 receptor D | 2,14E-01 | -0,68 |
| ENSG00000070669 | ASNS | asparagine synthetase (glutamine-hydrolyzing) | 9,84E-01 | -0,68 |
| ENSG00000181350 | LRRC75A | leucine rich repeat containing 75A | 6,76E-01 | -0,70 |
| ENSG00000177990 | DPY19L2 | dpy-19 like 2 | 1,00E+00 | -0,70 |
| ENSG00000106484 | MEST | mesoderm specific transcript | 5,00E-01 | -0,71 |
| ENSG00000134917 | ADAMTS8 | ADAM metallopeptidase with thrombospondin type 1 motif 8 | 9,11E-01 | -0,71 |
| ENSG00000197971 | MBP | myelin basic protein | 8,22E-01 | -0,72 |
| ENSG00000136999 | NOV | nephroblastoma overexpressed | 3,72E-03 | -0,73 |
| **gene_id** | **gene_name** | **gene_description** | **padj** | **log2FoldChange** |
| ENSG00000176890 | TYMS | thymidylate synthetase | 9,56E-01 | -0,74 |
| ENSG00000216775 | AL109918.1 | uncharacterized LOC730101 | 4,64E-01 | -0,75 |
| ENSG00000166473 | PKD1L2 | polycystin 1 like 2 (gene/pseudogene) | 8,03E-01 | -0,75 |
| ENSG00000248869 | LINC02511 | long intergenic non-protein coding RNA 2511 | 8,54E-01 | -0,75 |
| ENSG00000105516 | DBP | D-box binding PAR bZIP transcription factor | 7,03E-01 | -0,75 |
| ENSG00000245105 | A2M-AS1 | A2M antisense RNA 1 | 9,94E-01 | -0,76 |
| ENSG00000259456 | ADNP-AS1 | ADNP antisense RNA 1 | 9,39E-01 | -0,76 |
| ENSG00000115884 | SDC1 | syndecan 1 | 5,38E-01 | -0,76 |
| ENSG00000173535 | TNFRSF10C | TNF receptor superfamily member 10c | 3,19E-01 | -0,78 |
| ENSG00000235513 | AL035681.1 | novel transcript, antisense to L3MBTL2 | 1,00E+00 | -0,78 |
| ENSG00000007237 | GAS7 | growth arrest specific 7 | 6,53E-01 | -0,79 |
| ENSG00000222047 | C10orf55 | chromosome 10 open reading frame 55 | 1,00E+00 | -0,80 |
| ENSG00000270049 | AC009061.2 | uncharacterized LOC101927837 | 8,71E-01 | -0,80 |
| ENSG00000176438 | SYNE3 | spectrin repeat containing nuclear envelope family member 3 | 6,53E-01 | -0,80 |
| ENSG00000164056 | SPRY1 | sprouty RTK signaling antagonist 1 | 7,08E-01 | -0,81 |
| ENSG00000197769 | MAP1LC3C | microtubule associated protein 1 light chain 3 gamma | 9,84E-01 | -0,81 |
| ENSG00000085831 | TTC39A | tetratricopeptide repeat domain 39A | 1,47E-01 | -0,82 |
| ENSG00000079102 | RUNX1T1 | RUNX1 translocation partner 1 | 4,84E-01 | -0,83 |
| ENSG00000138735 | PDE5A | phosphodiesterase 5A | 9,40E-01 | -0,84 |
| ENSG00000106789 | CORO2A | coronin 2A | 1,00E+00 | -0,84 |
| ENSG00000265972 | TXNIP | thioredoxin interacting protein | 1,80E-01 | -0,85 |
| ENSG00000130592 | LSP1 | lymphocyte specific protein 1 | 6,44E-01 | -0,86 |
| ENSG00000126950 | TMEM35A | transmembrane protein 35A | 4,43E-01 | -0,86 |
| ENSG00000235865 | GSN-AS1 | GSN antisense RNA 1 | 6,88E-01 | -0,90 |
| ENSG00000214439 | FAM185BP | family with sequence similarity 185 member B, pseudogene | 1,00E+00 | -0,92 |
| ENSG00000162981 | FAM84A | family with sequence similarity 84 member A | 1,80E-01 | -0,95 |
| ENSG00000164742 | ADCY1 | adenylate cyclase 1 | 7,37E-01 | -0,96 |
| ENSG00000106125 | MINDY4 | MINDY lysine 48 deubiquitinase 4 | 8,22E-01 | -1,01 |
| ENSG00000182575 | NXPH3 | neurexophilin 3 | 6,43E-01 | -1,01 |
| ENSG00000139209 | SLC38A4 | solute carrier family 38 member 4 | 5,05E-01 | -1,01 |
| ENSG00000260552 | AC023043.1 | uncharacterized LOC101927809 | 8,33E-01 | -1,02 |
| ENSG00000249244 | AC110373.1 | glycerol kinase 2 pseudogene | 8,71E-01 | -1,03 |
| ENSG00000134539 | KLRD1 | killer cell lectin like receptor D1 | 9,45E-01 | -1,04 |
| ENSG00000137463 | MGARP | mitochondria localized glutamic acid rich protein | 1,00E+00 | -1,04 |
| ENSG00000104783 | KCNN4 | potassium calcium-activated channel subfamily N member 4 | 2,23E-01 | -1,05 |
| ENSG00000231407 | AL354732.1 | novel transcript | 7,92E-01 | -1,05 |
| ENSG00000245468 | LINC02447 | long intergenic non-protein coding RNA 2447 | 9,60E-01 | -1,07 |
| ENSG00000204072 | ARMCX7P | armadillo repeat containing X-linked 7, pseudogene | 8,56E-01 | -1,08 |
| **gene_id** | **gene_name** | **gene_description** | **padj** | **log2FoldChange** |
| ENSG00000213397 | HAUS7 | HAUS augmin like complex subunit 7 | 7,60E-01 | -1,08 |
| ENSG00000218052 | ADAMTS7P4 | ADAMTS7 pseudogene 4 | 5,62E-01 | -1,10 |
| ENSG00000130783 | CCDC62 | coiled-coil domain containing 62 | 5,78E-01 | -1,11 |
| ENSG00000257496 | AC025031.1 | novel transcript | 9,27E-01 | -1,12 |
| ENSG00000246985 | SOCS2-AS1 | SOCS2 antisense RNA 1 | 4,95E-01 | -1,14 |
| ENSG00000167676 | PLIN4 | perilipin 4 | 1,00E+00 | -1,14 |
| ENSG00000135333 | EPHA7 | EPH receptor A7 | 9,48E-01 | -1,15 |
| ENSG00000050555 | LAMC3 | laminin subunit gamma 3 | 7,96E-01 | -1,16 |
| ENSG00000258315 | C17orf49 | chromosome 17 open reading frame 49 | 1,00E+00 | -1,19 |
| ENSG00000213261 | EEF1B2P6 | eukaryotic translation elongation factor 1 beta 2 pseudogene 6 | 7,45E-01 | -1,22 |
| ENSG00000173253 | DMRT2 | doublesex and mab-3 related transcription factor 2 | 1,00E+00 | -1,24 |
| ENSG00000101098 | RIMS4 | regulating synaptic membrane exocytosis 4 | 5,72E-01 | -1,24 |
| ENSG00000284649 | AC009093.8 | BTG3 associated nuclear protein (BANP) pseudogene | 9,98E-01 | -1,26 |
| ENSG00000103888 | CEMIP | cell migration inducing hyaluronidase 1 | 6,78E-01 | -1,28 |
| ENSG00000185133 | INPP5J | inositol polyphosphate-5-phosphatase J | 1,00E+00 | -1,28 |
| ENSG00000137561 | TTPA | alpha tocopherol transfer protein | 3,80E-01 | -1,28 |
| ENSG00000215795 | AL390728.2 | peroxisomal biogenesis factor 5 (PEX5) pseudogene | 1,00E+00 | -1,29 |
| ENSG00000270441 | AC135506.1 | laminin, beta 1 (LAMB1) pseudogene | 1,00E+00 | -1,31 |
| ENSG00000151692 | RNF144A | ring finger protein 144A | 9,84E-01 | -1,33 |
| ENSG00000283376 | AC011591.2 | novel pseudogene | 1,00E+00 | -1,36 |
| ENSG00000184730 | APOBR | apolipoprotein B receptor | 9,64E-01 | -1,36 |
| ENSG00000103184 | SEC14L5 | SEC14 like lipid binding 5 | 7,70E-01 | -1,37 |
| ENSG00000165269 | AQP7 | aquaporin 7 | 7,28E-01 | -1,38 |
| ENSG00000117425 | PTCH2 | patched 2 | 5,03E-01 | -1,38 |
| ENSG00000240053 | LY6G5B | lymphocyte antigen 6 family member G5B | 8,54E-01 | -1,38 |
| ENSG00000164920 | OSR2 | odd-skipped related transciption factor 2 | 2,27E-01 | -1,42 |
| ENSG00000279863 | AC069547.1 | TEC | 7,26E-01 | -1,42 |
| ENSG00000143105 | KCNA10 | potassium voltage-gated channel subfamily A member 10 | 7,60E-01 | -1,44 |
| ENSG00000123560 | PLP1 | proteolipid protein 1 | 9,86E-01 | -1,46 |
| ENSG00000146006 | LRRTM2 | leucine rich repeat transmembrane neuronal 2 | 1,00E+00 | -1,49 |
| ENSG00000126861 | OMG | oligodendrocyte myelin glycoprotein | 9,15E-01 | -1,49 |
| ENSG00000148488 | ST8SIA6 | ST8 alpha-N-acetyl-neuraminide alpha-2,8-sialyltransferase 6 | 5,07E-01 | -1,54 |
| ENSG00000275025 | AC002401.3 | novel transcript, antisense to PDK2 | 1,00E+00 | -1,54 |
| ENSG00000081479 | LRP2 | LDL receptor related protein 2 | 7,96E-01 | -1,54 |
| ENSG00000149564 | ESAM | endothelial cell adhesion molecule | 1,00E+00 | -1,58 |
| ENSG00000204666 | AC010624.1 | uncharacterized LOC400710 | 1,00E+00 | -1,60 |
| ENSG00000179083 | FAM133A | family with sequence similarity 133 member A | 1,00E+00 | -1,67 |
| ENSG00000146678 | IGFBP1 | insulin like growth factor binding protein 1 | 8,22E-01 | -1,67 |
| **gene_id** | **gene_name** | **gene_description** | **padj** | **log2FoldChange** |
| ENSG00000160791 | CCR5 | C-C motif chemokine receptor 5 (gene/pseudogene) | 5,80E-01 | -1,68 |
| ENSG00000094963 | FMO2 | flavin containing monooxygenase 2 | 9,37E-01 | -1,70 |
| ENSG00000224592 | AL139158.2 | novel transcript | 4,19E-01 | -1,73 |
| ENSG00000132832 | AL139352.1 | novel transcript | 1,00E+00 | -1,76 |
| ENSG00000200361 | RF00019 |  | 1,00E+00 | -1,83 |
| ENSG00000231609 | AC007098.1 | uncharacterized LOC100132215 | 1,00E+00 | -1,87 |
| ENSG00000215458 | AATBC | apoptosis associated transcript in bladder cancer | 9,90E-01 | -1,97 |
| ENSG00000279817 | AC073911.3 | novel transcript | 1,00E+00 | -1,99 |
| ENSG00000226240 | LINC00381 | long intergenic non-protein coding RNA 381 | 1,00E+00 | -2,05 |
| ENSG00000229018 | PMS2P7 | PMS1 homolog 2, mismatch repair system component pseudogene 7 | 1,00E+00 | -2,13 |
| ENSG00000090512 | FETUB | fetuin B | 1,00E+00 | -2,15 |
| ENSG00000169313 | P2RY12 | purinergic receptor P2Y12 | 8,60E-01 | -2,15 |
| ENSG00000256663 | AC112777.1 | ubiquitin-like with PHD and ring finger domains 1 (UHRF1) pseudogene | 6,66E-01 | -2,18 |
| ENSG00000251023 | AC114980.1 | novel transcript | 7,33E-01 | -2,21 |
| ENSG00000231628 | AL133406.2 | novel transcript | 1,00E+00 | -2,22 |
| ENSG00000210156 | MT-TK | mitochondrially encoded tRNA lysine | 1,00E+00 | -2,22 |
| ENSG00000214770 | AL161756.1 | novel transcript | 1,00E+00 | -2,23 |
| ENSG00000240929 | HIST2H2BB | histone cluster 2 H2B family member b (pseudogene) | 1,00E+00 | -2,26 |
| ENSG00000254433 | AP001001.1 | novel transcript | 1,00E+00 | -2,30 |
| ENSG00000262873 | AC127496.5 | novel transcript | 1,00E+00 | -2,31 |
| ENSG00000153086 | ACMSD | aminocarboxymuconate semialdehyde decarboxylase | 1,00E+00 | -2,32 |
| ENSG00000278383 | AL031673.1 | novel transcript, antisense to ZNF337 | 1,00E+00 | -2,34 |
| ENSG00000170323 | FABP4 | fatty acid binding protein 4 | 9,84E-01 | -2,34 |
| ENSG00000285873 | AL445253.1 | uncharacterized LOC105376845 | 1,00E+00 | -2,37 |
| ENSG00000260615 | RPL23AP97 | ribosomal protein L23a pseudogene 97 | 1,00E+00 | -2,39 |
| ENSG00000172971 | UNC93B3 | unc-93 homolog B3, pseudogene | 1,00E+00 | -2,43 |
| ENSG00000224448 | AC005020.1 | ADP-ribosylation factor 6 (ARF6) pseudogene | 1,00E+00 | -2,49 |
| ENSG00000257322 | AC138123.1 | novel transcript, antisense to novel protein | 1,00E+00 | -2,52 |
| ENSG00000128254 | C22orf24 | chromosome 22 open reading frame 24 | 1,00E+00 | -2,55 |
| ENSG00000279730 | SETD8P1 | SET domain containing 8 pseudogene 1 | 1,00E+00 | -2,58 |
| ENSG00000285714 | AL159158.1 | novel transcript | 1,00E+00 | -2,60 |
| ENSG00000273056 | AL354694.1 | novel transcript | 1,00E+00 | -2,61 |
| ENSG00000146221 | TCTE1 | t-complex-associated-testis-expressed 1 | 1,00E+00 | -2,66 |
| ENSG00000073067 | CYP2W1 | cytochrome P450 family 2 subfamily W member 1 | 4,35E-01 | -2,66 |
| ENSG00000165548 | TMEM63C | transmembrane protein 63C | 1,00E+00 | -2,67 |
| ENSG00000232586 | KIAA1614-AS1 | KIAA1614 antisense RNA 1 | 1,00E+00 | -2,67 |
| ENSG00000133863 | TEX15 | testis expressed 15, meiosis and synapsis associated | 1,00E+00 | -2,89 |
| ENSG00000278131 | AC116050.1 | otopetrin 1 (OTOP1) pseudogene | 1,00E+00 | -2,90 |
| **gene_id** | **gene_name** | **gene_description** | **padj** | **log2FoldChange** |
| ENSG00000188039 | NWD1 | NACHT and WD repeat domain containing 1 | 1,00E+00 | -2,98 |
| ENSG00000228839 | PIK3IP1-AS1 | PIK3IP1 antisense RNA 1 (head to head) | 1,00E+00 | -3,01 |
| ENSG00000249167 | SEMA6A-AS2 | SEMA6A antisense RNA 2 | 1,00E+00 | -3,18 |
| ENSG00000169442 | CD52 | CD52 molecule | 1,00E+00 | -3,20 |
|  |  |  |  |  |
