# Supplementary material for: Insight Into the Metabolic Adaptations of Electrically Pulse-Stimulated Human Myotubes Using Global Analysis of the Transcriptome and Proteome
Source: Front Physiol. 2022 Jul 6;13:928195. doi: 10.3389/fphys.2022.928195 (PMC9298736; doi:10.3389/fphys.2022.928195)
Supplement: Supplementary file 2 [file Table2.DOCX]

**Supplementary table 2: Differentially secreted proteins induced by 24 h of electrical pulse stimulation (EPS, 2ms, 10V, 0.1Hz)**

| **Protein IDs** | **Gene names** | **Protein names** | **p-value** | **Fold change** |
| --- | --- | --- | --- | --- |
| P31431 | SDC4 | Syndecan-4 | 1,95E-03 | 6,03 |
| P48307 | TFPI2 | Tissue factor pathway inhibitor 2 | 3,58E-05 | 5,86 |
| P98066 | TNFAIP6 | Tumor necrosis factor-inducible gene 6 protein | 3,19E-02 | 5,50 |
| P22105 | TNXB | Tenascin-X | 1,83E-02 | 4,01 |
| Q92626 | PXDN | Peroxidasin homolog | 6,76E-05 | 3,80 |
| P03956 | MMP1 | Interstitial collagenase | 1,40E-02 | 3,74 |
| P10145 | CXCL8 | Interleukin-8 | 5,72E-03 | 3,72 |
| P42830 | CXCL5 | C-X-C motif chemokine 5 | 4,34E-02 | 3,61 |
| P29279 | CTGF | Connective tissue growth factor | 1,36E-02 | 3,44 |
| P09341 | CXCL1 | Growth-regulated alpha protein;GRO-alpha(4-73);GRO-alpha(5-73);GRO-alpha(6-73) | 3,94E-02 | 3,17 |
| P19876 | CXCL3 | C-X-C motif chemokine 3;GRO-gamma(5-73) | 3,94E-02 | 3,17 |
| P19875 | CXCL2 | C-X-C motif C-X-C motif chemokine 2;GRO-beta(5-73) | 3,94E-02 | 3,17 |
| Q9BS26 | ERP44 | Endoplasmic reticulum resident protein 44 | 8,20E-03 | 3,14 |
| Q14767 | LTBP2 | Latent-transforming growth factor beta-binding protein 2 | 4,40E-06 | 2,88 |
| Q76M96 | CCDC80 | Coiled-coil domain-containing protein 80 | 1,38E-04 | 2,31 |
| Q05707 | COL14A1 | Collagen alpha-1(XIV) chain | 1,10E-02 | 2,30 |
| Q15582 | TGFBI | Transforming growth factor-beta-induced protein ig-h3 | 6,20E-04 | 2,30 |
| O95967 | EFEMP2 | EGF-containing fibulin-like extracellular matrix protein 2 | 1,40E-03 | 2,19 |
| O95633 | FSTL3 | Follistatin-related protein 3 | 2,02E-02 | 2,11 |
| P15531 | NME1 | Nucleoside diphosphate kinase A | 4,92E-02 | 2,07 |
| P10124 | SRGN | Serglycin | 4,27E-02 | 1,96 |
| Q13177 | PAK2 | Serine/threonine-protein kinase PAK 2;PAK-2p27;PAK-2p34 | 3,79E-02 | 1,95 |
| P02751 | FN1 | Fibronectin;Anastellin;Ugl-Y1;Ugl-Y2;Ugl-Y3 | 1,55E-05 | 1,93 |
| P98160 | HSPG2 | Basement membrane-specific heparan sulfate proteoglycan core protein;Endorepellin;LG3 peptide | 2,96E-03 | 1,88 |
| P08254 | MMP3 | Stromelysin-1 | 1,81E-02 | 1,87 |
| P31942 | HNRNPH3 | Heterogeneous nuclear ribonucleoprotein H3 | 3,74E-02 | 1,84 |
| P12956 | XRCC6 | X-ray repair cross-complementing protein 6 | 1,48E-03 | 1,81 |
| P26641 | EEF1G | Elongation factor 1-gamma | 2,37E-03 | 1,74 |
| Q9UNM6 | PSMD13 | 26S proteasome non-ATPase regulatory subunit 13 | 3,39E-02 | 1,72 |
| P10909 | CLU | Clusterin;Clusterin beta chain;Clusterin alpha chain | 2,89E-02 | 1,71 |
| Q12805 | EFEMP1 | EGF-containing fibulin-like extracellular matrix protein 1 | 1,02E-02 | 1,70 |
| Q68BL8 | OLFML2B | Olfactomedin-like protein 2B | 4,79E-02 | 1,67 |
| P07585 | DCN | Decorin | 3,46E-04 | 1,63 |
| P08572 | COL4A2 | Collagen alpha-2(IV) chain;Canstatin | 8,89E-03 | 1,62 |
| P80723 | BASP1 | Brain acid soluble protein 1 | 1,47E-02 | 1,61 |
| Q99715 | COL12A1 | Collagen alpha-1(XII) chain | 1,03E-03 | 1,60 |
| **Protein IDs** | **Gene names** | **Protein names** | **p-value** | **Fold change** |
| P12111 | COL6A3 | Collagen alpha-3(VI) chain | 4,72E-03 | 1,59 |
| Q7Z7G0 | ABI3BP | Target of Nesh-SH3 | 9,25E-03 | 1,59 |
| P26022 | PTX3 | Pentraxin-related protein PTX3 | 1,02E-02 | 1,58 |
| P02649 | APOE | Apolipoprotein E | 1,72E-02 | 1,58 |
| P30041 | PRDX6 | Peroxiredoxin-6 | 4,18E-03 | 1,56 |
| P37802 | TAGLN2 | Transgelin-2 | 6,73E-03 | 1,54 |
| Q53GG5 | PDLIM3 | PDZ and LIM domain protein 3 | 3,37E-02 | 1,53 |
| P35555 | FBN1 | Fibrillin-1 | 4,29E-04 | 1,51 |
| P28300 | LOX | Protein-lysine 6-oxidase | 1,22E-02 | 1,51 |
| Q8IX30 | SCUBE3 | Signal peptide, CUB and EGF-like domain-containing protein 3 | 1,07E-02 | 1,50 |
| Q15084 | PDIA6 | Protein disulfide-isomerase A6 | 5,46E-03 | 1,48 |
| P61158 | ACTR3 | Actin-related protein 3 | 6,39E-03 | 1,48 |
| P07355 | ANXA2 | Annexin A2 | 8,40E-03 | 1,46 |
| A6NMY6 | ANXA2P2 | Putative annexin A2-like protein | 8,40E-03 | 1,46 |
| Q14697 | GANAB | Neutral alpha-glucosidase AB | 1,02E-02 | 1,45 |
| P02452 | COL1A1 | Collagen alpha-1(I) chain | 1,35E-04 | 1,42 |
| P02461 | COL3A1 | Collagen alpha-1(III) chain | 5,27E-03 | 1,40 |
| Q15113 | PCOLCE | Procollagen C-endopeptidase enhancer 1 | 3,77E-02 | 1,39 |
| P11047 | LAMC1 | Laminin subunit gamma-1 | 3,72E-03 | 1,34 |
| P12277 | CKB | Creatine kinase B-type | 3,64E-02 | 1,28 |
| P35052 | GPC1 | Glypican-1;Secreted glypican-1 | 2,36E-02 | 1,26 |
| P07737 | PFN1 | Profilin-1 | 3,22E-02 | -1,13 |
| P63104 | YWHAZ | 14-3-3 protein zeta/delta | 7,55E-03 | -1,29 |
| P62258 | YWHAE | 14-3-3 protein epsilon | 5,37E-03 | -1,29 |
| P07858 | CTSB | Cathepsin B;Cathepsin B light chain;Cathepsin B heavy chain | 2,88E-02 | -1,31 |
| P07237 | P4HB | Protein disulfide-isomerase | 2,79E-02 | -1,34 |
| Q08629 | SPOCK1 | Testican-1 | 4,06E-03 | -1,34 |
| P23142 | FBLN1 | Fibulin-1 | 3,91E-02 | -1,41 |
| P22626 | HNRNPA2B1 | Heterogeneous nuclear ribonucleoproteins A2/B1 | 1,42E-02 | -1,41 |
| P14625 | HSP90B1 | Endoplasmin | 9,60E-03 | -1,43 |
| Q9Y490 | TLN1 | Talin-1 | 2,55E-02 | -1,45 |
| P68032 | ACTC1 | Actin, alpha cardiac muscle 1 | 6,97E-03 | -1,47 |
| P62736 | ACTA2 | Actin, aortic smooth muscle | 6,97E-03 | -1,47 |
| P63267 | ACTG2 | Actin, gamma-enteric smooth muscle | 6,97E-03 | -1,47 |
| P62140 | PPP1CB | Serine/threonine-protein phosphatase PP1-beta catalytic subunit | 2,91E-03 | -1,49 |
| Q15942 | ZYX | Zyxin | 4,81E-02 | -1,56 |
| P27816 | MAP4 | Microtubule-associated protein 4 | 2,39E-04 | -1,57 |
| Q9H488 | POFUT1 | GDP-fucose protein O-fucosyltransferase 1 | 9,31E-03 | -1,58 |
| **Protein IDs** | **Gene names** | **Protein names** | **p-value** | **Fold change** |
| Q71U36 | TUBA1A | Tubulin alpha-1A chain | 3,31E-03 | -1,58 |
| P68363 | TUBA1B | Tubulin alpha-1B chain | 3,31E-03 | -1,58 |
| Q6PEY2 | TUBA3E | Tubulin alpha-3E chain | 3,31E-03 | -1,58 |
| P68366 | TUBA4A | Tubulin alpha-4A chain | 3,31E-03 | -1,58 |
| Q01082 | SPTBN1 | Spectrin beta chain, non-erythrocytic 1 | 3,50E-02 | -1,60 |
| P06733 | ENO1 | Alpha-enolase | 3,25E-03 | -1,63 |
| P30085 | CMPK1 | UMP-CMP kinase | 2,27E-02 | -1,65 |
| P40261 | NNMT | Nicotinamide N-methyltransferase | 1,12E-02 | -1,65 |
| P48681 | NES | Nestin | 1,69E-03 | -1,68 |
| Q13200 | PSMD2 | 26S proteasome non-ATPase regulatory subunit 2 | 3,81E-02 | -1,68 |
| P67936 | TPM4 | Tropomyosin alpha-4 chain | 7,25E-03 | -1,69 |
| P62328 | TMSB4X | Thymosin beta-4 | 7,45E-04 | -1,71 |
| P13489 | RNH1 | Ribonuclease inhibitor | 2,36E-04 | -1,72 |
| P13667 | PDIA4 | Protein disulfide-isomerase A4 | 3,32E-03 | -1,72 |
| P00558 | PGK1 | Phosphoglycerate kinase 1 | 5,05E-03 | -1,73 |
| Q6NZI2 | PTRF | Polymerase I and transcript release factor | 1,71E-02 | -1,76 |
| P11142 | HSPA8 | Heat shock cognate 71 kDa protein | 1,53E-03 | -1,77 |
| Q06830 | PRDX1 | Peroxiredoxin-1 | 2,07E-03 | -1,78 |
| Q99538 | LGMN | Legumain | 4,09E-02 | -1,80 |
| P16949 | STMN1 | Stathmin | 8,62E-03 | -1,83 |
| P07951 | TPM2 | Tropomyosin beta chain | 3,01E-02 | -1,85 |
| P63261 | ACTG1 | Actin, cytoplasmic 2 | 1,33E-03 | -1,86 |
| Q15019 | SEPT2 | Septin-2 | 7,94E-04 | -1,87 |
| P08238 | HSP90AB1 | Heat shock protein HSP 90-beta | 1,97E-03 | -1,91 |
| P27695 | APEX1 | DNA-(apurinic or apyrimidinic site) lyase, mitochondrial | 1,09E-02 | -1,98 |
| Q86UP2 | KTN1 | Kinectin | 1,34E-02 | -2,00 |
| Q00610 | CLTC | Clathrin heavy chain 1 | 2,59E-02 | -2,08 |
| P09493 | TPM1 | Tropomyosin alpha-1 chain | 2,68E-02 | -2,13 |
| Q9BUF5 | TUBB6 | Tubulin beta-6 chain | 2,14E-03 | -2,15 |
| P04075 | ALDOA | Fructose-bisphosphate aldolase A | 2,51E-02 | -2,15 |
| P05121 | SERPINE1 | Plasminogen activator inhibitor 1 | 2,72E-02 | -2,16 |
| O00754 | MAN2B1 | Lysosomal alpha-mannosidase | 8,56E-03 | -2,17 |
| P07900 | HSP90AA1 | Heat shock protein HSP 90-alpha | 4,31E-04 | -2,18 |
| O14907 | TAX1BP3 | Tax1-binding protein 3 | 1,19E-02 | -2,28 |
| P56537 | EIF6 | Eukaryotic translation initiation factor 6 | 2,32E-04 | -2,29 |
| Q13162 | PRDX4 | Peroxiredoxin-4 | 2,78E-02 | -2,36 |
| Q96TA1 | FAM129B | Niban-like protein 1 | 4,53E-02 | -2,50 |
| P17096 | HMGA1 | High mobility group protein HMG-I/HMG-Y | 3,00E-02 | -2,55 |
| **Protein IDs** | **Gene names** | **Protein names** | **p-value** | **Fold change** |
| P09936 | UCHL1 | Ubiquitin carboxyl-terminal hydrolase isozyme L1 | 3,74E-02 | -2,65 |
| P30153 | PPP2R1A | Serine/threonine-protein phosphatase 2A 65 kDa regulatory subunit A alpha isoform | 2,17E-02 | -2,67 |
| P31946 | YWHAB | 14-3-3 protein beta/alpha | 2,47E-02 | -2,71 |
| Q14247 | CTTN | Src substrate cortactin | 7,03E-03 | -2,77 |
| Q8NC51 | SERBP1 | Plasminogen activator inhibitor 1 RNA-binding protein | 2,12E-02 | -2,87 |
| P63244 | GNB2L1 | Guanine nucleotide-binding protein subunit beta-2-like 1 | 1,90E-02 | -2,96 |
| P27348 | YWHAQ | 14-3-3 protein theta | 4,02E-02 | -3,00 |
| P00441 | SOD1 | Superoxide dismutase [Cu-Zn] | 6,74E-03 | -3,04 |
| Q9UKY7 | CDV3 | Protein CDV3 homolog | 3,50E-02 | -3,08 |
| P11766 | ADH5 | Alcohol dehydrogenase class-3 | 1,67E-02 | -3,18 |
| P09972 | ALDOC | Fructose-bisphosphate aldolase C | 5,15E-03 | -3,30 |
| P05155 | SERPING1 | Plasma protease C1 inhibitor | 3,77E-02 | -3,31 |
| O75368 | SH3BGRL | SH3 domain-binding glutamic acid-rich-like protein | 8,04E-03 | -3,40 |
| P63220 | RPS21 | 40S ribosomal protein S21 | 1,69E-03 | -3,42 |
| P05387 | RPLP2 | 60S acidic ribosomal protein P2 | 3,19E-02 | -3,45 |
| P09382 | LGALS1 | Galectin-1 | 9,35E-03 | -3,50 |
| P32119 | PRDX2 | Peroxiredoxin-2 | 2,80E-02 | -3,51 |
| P07437 | TUBB | Tubulin beta chain | 4,21E-02 | -3,65 |
| Q8NBS9 | TXNDC5 | Thioredoxin domain-containing protein 5 | 1,21E-02 | -3,65 |
| P60903 | S100A10 | Protein S100-A10 | 4,44E-02 | -3,69 |
| P07686 | HEXB | Beta-hexosaminidase subunit beta | 1,65E-02 | -3,71 |
| P29692 | EEF1D | Elongation factor 1-delta | 3,27E-03 | -3,78 |
| P22314 | UBA1 | Ubiquitin-like modifier-activating enzyme 1 | 1,82E-02 | -3,84 |
| P08865 | RPSA | 40S ribosomal protein SA | 5,22E-03 | -4,00 |
| P05388 | RPLP0 | 60S acidic ribosomal protein P0 | 1,08E-02 | -4,24 |
| Q8NHW5 | RPLP0P6 | 60S acidic ribosomal protein P0-like | 1,08E-02 | -4,24 |
| Q9NQC3 | RTN4 | Reticulon-4 | 1,41E-02 | -4,48 |
| O75083 | WDR1 | WD repeat-containing protein 1 | 1,32E-03 | -4,67 |
| Q9H299 | SH3BGRL3 | SH3 domain-binding glutamic acid-rich-like protein 3 | 1,81E-02 | -4,78 |
| P60033 | CD81 | CD81 antigen | 5,59E-03 | -5,16 |
| Q3ZCM7 | TUBB8 | Tubulin beta-8 chain | 5,49E-03 | -6,30 |
| Q01105 | SET | Protein SET | 2,33E-03 | -6,31 |
| P0DME0 | SETSIP | Protein SETSIP | 2,33E-03 | -6,31 |
| Q5VTE0 | EEF1A1P5 | Putative elongation factor 1-alpha-like 3 | 4,90E-03 | -12,80 |
| P68104 | EEF1A1 | Elongation factor 1-alpha 1 | 4,90E-03 | -12,80 |
| Q05639 | EEF1A2 | Elongation factor 1-alpha 2 | 4,90E-03 | -12,80 |
| P04406 | GAPDH | Glyceraldehyde-3-phosphate dehydrogenase | 8,27E-04 | -14,47 |
